# Statistical modeling of biomedical corpora: mining the Caenorhabditis Genetic Center Bibliography for genes related to life span

**DOI:** 10.1186/1471-2105-7-250

**Published:** 2006-05-08

**Authors:** DM Blei, K Franks, MI Jordan, IS Mian

**Affiliations:** 1 Computer Science Department, Princeton University, Princeton, New Jersey 08540 USA; 2 Life Sciences Division, Lawrence Berkeley National Laboratory, Berkeley, California 94720-8265, USA; 3 Department of Statistics, University of California Berkeley, Berkeley, California 94720, USA; 4 Department of EECS, University of California Berkeley, Berkeley, California 94720, USA

## Abstract

**Background:**

The statistical modeling of biomedical corpora could yield integrated, coarse-to-fine views of biological phenomena that complement discoveries made from analysis of molecular sequence and profiling data. Here, the potential of such modeling is demonstrated by examining the 5,225 free-text items in the *Caenorhabditis *Genetic Center (CGC) Bibliography using techniques from statistical information retrieval. Items in the CGC biomedical text corpus were modeled using the Latent Dirichlet Allocation (LDA) model. LDA is a hierarchical Bayesian model which represents a document as a random mixture over latent topics; each topic is characterized by a distribution over words.

**Results:**

An LDA model estimated from CGC items had better predictive performance than two standard models (unigram and mixture of unigrams) trained using the same data. To illustrate the practical utility of LDA models of biomedical corpora, a trained CGC LDA model was used for a retrospective study of nematode genes known to be associated with life span modification. Corpus-, document-, and word-level LDA parameters were combined with terms from the Gene Ontology to enhance the explanatory value of the CGC LDA model, and to suggest additional candidates for age-related genes. A novel, pairwise document similarity measure based on the posterior distribution on the topic simplex was formulated and used to search the CGC database for "homologs" of a "query" document discussing the life span-modifying *clk-2 *gene. Inspection of these document homologs enabled and facilitated the production of hypotheses about the function and role of *clk-2*.

**Conclusion:**

Like other graphical models for genetic, genomic and other types of biological data, LDA provides a method for extracting unanticipated insights and generating predictions amenable to subsequent experimental validation.

## Background

In the design, analysis and interpretation of experiments, biomedical and clinical researchers encounter the problem of evaluating and summarizing prior knowledge on the subject under investigation. Traditional solutions include examining articles in scientific journals, primarily via PubMed access to MEDLINE, and interrogating WWW-based sources such as Entrez Gene [1] and Online Mendelian Inheritance in Man (OMIM) [2]. Consider a scenario in which a researcher seeks enhanced knowledge about a protein implicated in aging. Typically, the steps involved in addressing this problem include interrogating structured data resources: searching protein sequence databases to identify homologs in other species, querying warehouses of genomic information to determine key non-coding regions and polymorphisms, examining collections of high-throughput molecular profiling data sets to ascertain genes with similar patterns of expression, probing ontologies such as the Gene Ontology (GO) [3] to uncover other genes with similar patterns of annotation, and so on. The entire procedure is accompanied by examination of the literature to determine, for example, classes of proteins mentioned in the same article as the putative gerontogene.

Advanced techniques and sophisticated tools for interacting with structured data are well known, widely available and include BLAST [4] for sequence databases, Ensembl [5] and the UCSC Browser [6] for genomes, GEO [7] for transcript profiles, and tools available from the GO that allow navigation of terms in the ontology. This is less true for the scientific literature. Given the time-consuming yet critical importance of synthesizing information in text corpora such as MEDLINE, the problem of making data interpretation a more systematic and automated endeavor is emerging as an important topic of research (see, for example, [8-10]). The development of strategies capable of providing a user the ability to assimilate and act upon information present in resources of structured and unstructured data remains an important goal.

The primary aim of biomedical text mining is the systematic analysis of document collections such as MEDLINE abstracts and full-text journal articles with the goal of generating useful and unanticipated scientific discoveries (for recent reviews of current methods and illustrative applications, see [11-13]). Examples of tasks addressed by text mining methods include identifying literature relevant to specific molecules, finding associations between genes and diseases, determining putative functions for proteins, and predicting regulatory networks.

A common approach to text mining is to treat the problem as one of natural language processing (NLP) [14]. NLP methods concentrate on the linguistic structure of documents and make explicit use of syntactic, relational, and ontological knowledge. In biology, such approaches [15] have been employed for information extraction: the task of ascertaining facts, relations, and entities in unstructured written language such as protein-protein interactions, protein subcellular location, and gene names. Tools based on these ideas include Textpresso [16] and Telemakus [17]. Elsewhere, NLP has been used in conjunction with LocusLink and GO to compare OMIM to MEDLINE [18].

Recently, nouns extracted from MEDLINE abstracts tagged with parts of speech were combined with knowledge from other sources, Principal Component Analysis, and means linkage clustering to find associations between genes and phenotypes [19]. In general, NLP can be effective in circumscribed domains where linguistic knowledge is available and the terminology evolves slowly, is consistent, largely unambiguous, and relatively simple. However, the paucity and incomplete nature of such information for biomedical corpora suggests that the full potential of text mining in biology remains unrealized.

An alternative to NLP is to frame the problem from the perspective of information retrieval (IR) [20]. Statistical IR methods explore large quantities of information and often involve capabilities for clustering, classifying, categorizing, summarizing, and detecting novel, similar and relevant objects. The most successful testaments to the real-world utility of IR techniques are Internet search engines. Thus, statistical IR models of biological document collections could reveal rich, complex, and previously unappreciated relationships. Such results would complement insights derived from analysis of molecular profiling, protein-protein interaction, gene knock-out, and similar types of data. With systematic deployment of IR tools, the interrogation of biomedical corpora could become as routine and indispensable a part of research as analyzing genomic and genetic data is today. The analogy is more than superficial and extends to the direct use of IR techniques such as singular value decomposition in bioinformatics (see for example [21]). Thus, the common mathematical foundations for algorithms that underpin IR and genome analysis make it possible to envision integrated procedures that combine primary biological data with biological corpora.

**Figure 1:**
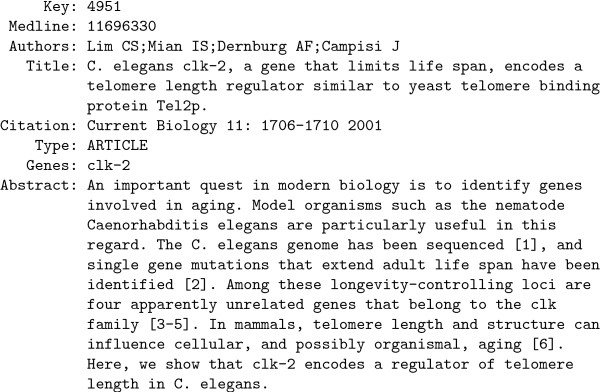
One of the 5,225 free-text items in the CGC Bibliography in its original form.

This work describes an application of statistical IR methodology to the analysis of a biomedical text corpus, the *Caenorhabditis *Genetic Center (CGC) Bibliography (Figure 1). The specific model at the heart of this study is the Latent Dirichlet Allocation (LDA) model [22], a hierarchical Bayesian model employed previously to analyze text corpora and to annotate images [23]. Recently, LDA has been used to extract and analyze the topics present in a document corpus consisting of articles published in the journal *Proceedings of the National Academy of Sciences *[24].

In general, IR methods assume that the order of words in a document can be neglected and view documents as "bags of words." The loss of information incurred by ignoring word order is offset by the ability to devise efficient computational algorithms that are viable for large corpora. Although there is no theoretical justification for casting a document in this manner, the practical benefits and utility of doing so are considerable. The LDA model considered here is a model for a corpus viewed as a collection of bags of words. It assumes that each word of each document is generated by one of several "topics"; each topic is associated with a different conditional distribution over a fixed vocabulary. The same set of topics is used to generate the entire set of documents in a collection but each document reflects these topics with different relative proportions. Thus, LDA is a mixture of mixtures model, *i.e*., the mixture components are shared across all documents but each document exhibits different mixture proportions. As a generative probabilistic model, the LDA can handle unseen or novel data, *i.e*., a document that was not one of the bag of words used to estimate the model.

The fundamental entities in LDA, random variables representing topics and words, are grouped together in such a way to form a corpus, *i.e*., a group of groups of words. The hierarchical nature of the model stems from the fact that documents are modeled as probability distributions across topics, and topics are modeled as probability distributions across words. A notable virtue of LDA is that a given topic can occur with high probability in multiple documents, and that a given word can occur with high probability in multiple topics. Topics are treated as latent variables, namely entities that are not present explicitly in the data (a set of sequences of words), but are presumed to be present implicitly and are to be inferred by statistical analysis.

To analyze the CGC Bibliography, each item in the corpus was recast as a bag of words and the resultant data set of documents was used to estimate the parameters of three different statistical IR models. The predictive performance of the LDA model was better than that of two simpler bag of words models, a unigram model and a mixture of unigrams model, trained on the same data set. The potential of LDA in assisting biological studies was illustrated by considering the phenomenon of nematode aging. In order to illuminate the hidden factors permeating a corpus and captured by the topics discovered by a trained CGC LDA model, LDA topics were labeled via an automated process that assigned words from the CGC vocabulary (corpus-based labels) and GO terms (ontology-based labels) to each topic. Examination of these labels indicated that the CGC topics captured meaningful and plausible facets of nematode biology. To investigate aging, topics whose corpus-based labels included many CGC words corresponding to the names of genes known to influence life span were identified. For the two topics with the greatest number of such CGC-based topic labels, novel candidates for age-related genes were equated with other CGC-based topic labels that corresponded to gene names (guilt-by-association). Finally, an LDA-based measure of pairwise document similarity was devised and used to address the problem of searching a database of documents to determine topic-space homologs of a query document. Inspection of the "document homologs" of the CGC item shown in Figure 1 resulted in enhanced understanding of the biology of the *clk-2 *gene.

This work highlights the potential and utility of LDA in organizing and exploiting one type of widely available information resource, a collection of documents in the form of free or unstructured text. However, researchers are faced with a plethora of resources including images and structured data such as molecular sequences, transcript profiles, disease information, and so on. Thus, there is a compelling need for techniques and systems able to condense, integrate and present large amounts of disparate data to a user. This paper concludes with a discussion of how the family of probabilistic graphical models, of which LDA is a specific example, provides a framework for integrating heterogeneous data and thus meets this challenge.

## Results

### LDA outperforms mixture of unigrams, unigram and random models

In order to compare different models of text, a data set of *C. elegans *related documents was created. In particular, each CGC Bibliography free-text item was transformed into a bag of words yielding a corpus of *M *= 5, 225 documents and a *V *= 28, 971 word vocabulary.

**Figure 2:**
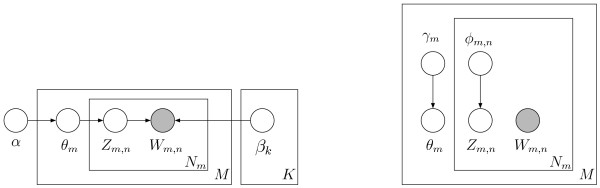
Graphical model representation of the LDA model (left) and the variational distribution used to approximate the posterior in LDA (right) [22]. LDA defines a distribution on a collection of documents in much the same manner that a profile hidden Markov model yields a distribution on a set of (biological) sequences [31]. The corpus depicted contains *M *documents and each is a sequence of *N *words. Open circles are parameters (*α*, *β*, *γ*, *φ*) or latent variables (*θ*, *z*). The shaded circle is the observed word variable (*w*) and boxes (plates) represent replicates. The Dirichlet parameter, *α*, and topic-word matrix, *β*, are corpus-level parameters sampled once in the process of generating a corpus. The topic proportions, *θ*, is a document-level variable sampled from αonce per document. The topic, *z*, is a word-level variable sampled from *θ *once for each word in a document. Formally, a *K*-topic LDA specifies a two-level probabilistic process that generates a document as follows, (i) a *K*-dimensional vector, *θ*, is chosen from the distribution *p*(*θ|α*), and (ii) words are sampled repeatedly from the document-specific mixture distribution, *p*(*w|θ*). Exact inference and parameter estimation involve calculating the posterior distribution on a document *p*(*θ*, **z**|**w**, *α, β*). This is intractable because the latent variables are coupled via the edge between *θ *and *z*. The posterior can be approximated by computing the variational Dirichlet parameter *γ *and the variational multinomial parameter *φ *for each word in the document. The subscripts *m*, *n*, and *k* on a parameter (β, γ, φ) or variable (*θ*, *z*, *w) *donate the *m *th document, *nth* word and kth topic respectively. Note that the Dirichlet variable *α *is a distinct component of the probability model and not merely an expression of uncertainty about a parameter. This differs from profile hidden Markov models where a mixture of Dirichlet distributions is used as a prior for amino acid/nucleotide probability distributions.

**Figure 3:**
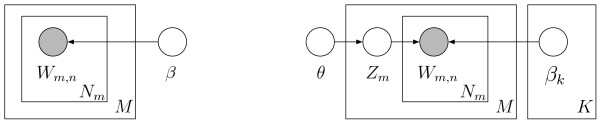
Graphical model representation of a mixture of unigrams model with *K *latent topics (right) and a unigram model (left). The corpus depicted contains *M *documents and each is a sequence of *N *words. Open circles represent latent variables (*z*)or parameters (*β*, *θ*). Each shaded circle is an observed word variable (*w*). Boxes (plates) represent replicates. The subscripts *m*, *n* abd *k *on a parameter (β, θ) or variable (*z*, *w*) donate the mth document, nth word and kth topic respectively.A mixture of unigrams generates all the words in a given document from exactly one topic, *z*. This differs from the LDA model where a single document can express multiple topics (Figure 2). Note that the naive Bayes model used to cluster transcript profiling data [41-43] has the same topology as the mixture of unigrams but the observed variables are continuous-valued expression measurements rather than discrete words.

**Figure 4:**
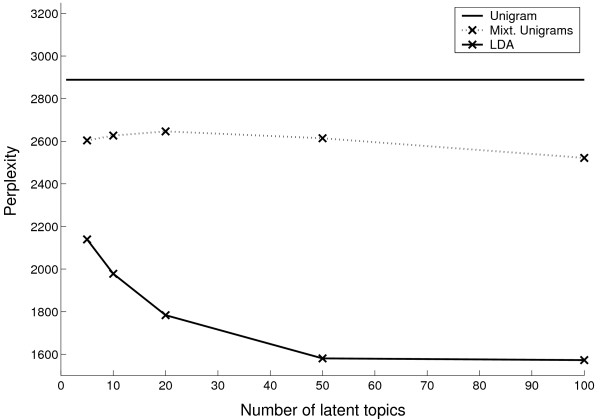
The perplexity of LDA, mixture of unigrams, and unigram models estimated and evaluated on the CGC corpus. The score of test documents is shown against the number of latent topics (the perplexity of the unigram is constant because this statistical model has no notion of latent topics).

The generalization performance of three statistical models was assessed: an LDA model (Figure 2), a mixture of unigrams model (right, Figure 3), and a "baseline" unigram model (left, Figure 3). A model was trained using 90% of the 5,225 documents in the CGC corpus and tested on the remaining 10%. LDA and mixture of unigrams models with *K *= 5, 10, 20, 50, and 100 latent topics were estimated; a single unigram model was estimated because such models harbor no notion of topic. The perplexity (inverse of the per-word likelihood) of the held-out test set of *J *= 525 documents (Equation 6) was computed for each trained model. Figure 4 shows the generalization performance of each model as a function of the number of latent topics. LDA has consistently smaller perplexity scores than the two extant models indicating better performance on unseen documents. Since the perplexity of 50- and 100-topic LDA's is low and similar, a latent space with 50 topics appears to provide a parsimonious description of the CGC corpus.

**Figure 5:**
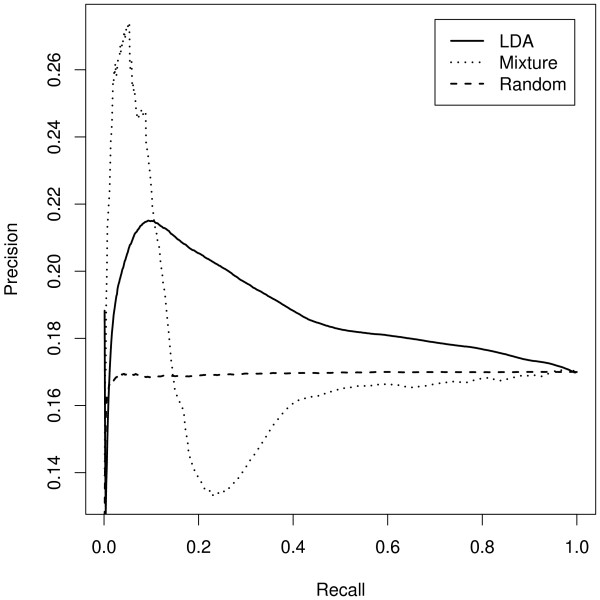
Precision/recall (PR) curves for three models of text (LDA, mixture of unigrams, random) and the task of retrieving a set of aging-related documents (842 CGC items that refer to one or more of the genes listed in Table 1). Precision is the fraction of documents in a list that are relevant (related to aging) whereas recall is the fraction of relevant documents in the list. For a desired level of recall, for example 70%, there is a corresponding precision. The graph shows average precision against average recall. Although each point is a mean of 842 pairs of precision and recall values, the standard error is negligible and so not depicted.

The ability of three specific models to retrieve a set of 842 aging-related documents in the collection of 5,225 CGC documents was assessed: a 50-topic LDA model estimated using all documents in the corpus, a 50-topic mixture of unigrams model estimated using all documents, and a model which ordered all documents randomly. For each model, an average precision/recall (PR) curve was constructed by computing the ranking of other documents given each aging-related document as a query, and the average F1 measure was computed. Figure 5 shows average PR curves for the three models. The average F1 measure (standard error) for the LDA model, the mixture of unigrams model and a random model is 0.30(2.86*e *– 06), 0.22(7.85*e *– 06), and 0.29(3.02*e *– 05) respectively. Although the F1 values for the LDA and random models are similar, the smaller standard error of the LDA model indicates its superiority to the random model. In addition, the average PR curves indicate that the LDA places more of the age-related documents higher up its rankings than the random model. Thus, of the three models investigated, LDA is best able to retrieve the set of related documents. Overfitting by the mixture of unigrams model results in a performance worse than the random model.

All subsequent discussion of an LDA model and/or a mixture of unigrams model pertain to a *K *= 50 topic model estimated using all *M *= 5, 225 CGC documents in the corpus.

### LDA latent topics embody concepts associated with nematode biology

A systematic strategy for clarifying the nature of the hidden factors permeating a corpus was devised and applied to a CGC LDA. Topic annotation (topic labeling) is defined as an automated process that creates a verbose (compact) description of an LDA topic. The method designed to annotate and label topics exploited the corpus-level parameter *β *(Figure 2). The *K *× *V *topic-word matrix *β *collates the multinomial distributions over the *V *words in the vocabulary that characterize the *K *topics. For a given LDA model of a particular corpus, the *kth *row specifies the topic-specific word distribution for topic *k *and an element, *β_*kv*_*, denotes the likelihood of the *v*th word given the *kth *topic. For each of the *K *= 50 topics in the CGC LDA model, the *V *= 28, 971 *β*_*kv *_values were ordered and used to generate a word rank versus topic-specific word probability plot. In every case, the 500 top-ranked words accounted for most of the probability mass. Thus, these 500 high probability CGC words were designated topic annotation words (the same word from the *V*-wordvocabulary could annotate multiple topics).

Two different approaches were used to create labels for each topic. Corpus-based topic labels are topic annotation words that are unique to a topic and represent descriptors applicable to only one topic. CGC-based topic labels were equated with topic annotation words that were not assigned to any of the other 49 topics, *i.e*., the 50 sets of CGC-based topic labels formed disjoint subsets of words from the CGC vocabulary. Ontology-based topic labels are the outcome of filtering topic annotation words using an external knowledge source and represent descriptors applicable to one or more topic. The ontology exploited here was the GO. The relationship between GO controlled vocabulary terms can be depicted as a directed acyclic graph (DAG). Each node corresponds to a term from one of three aspects, for example, "exodeoxyribonuclease," "mitochondrial derivative" and "ethylene mediated signaling pathway" are exemplars of GO terms from the "Molecular Function," "Cellular Component" and "Biological Function" aspects respectively. The structure of the DAG underpinning the GO vocabulary defines semantic relationships amongst terms so that, for example, the node for the GO term "intracellular" is a parent of the node for the more specific GO term "nucleus." Recall that the topic annotation words for topic *k *are the 500 words from the CGC vocabulary that best characterize the topic. These 500 words were mapped to nodes in the GO DAG. A node where a topic annotation word coincided with a GO term was designated an explicit node. GO-based topic labels were equated with the GO terms for both explicit nodes and the children and grandchildren of explicit nodes.

**Figure 6:**
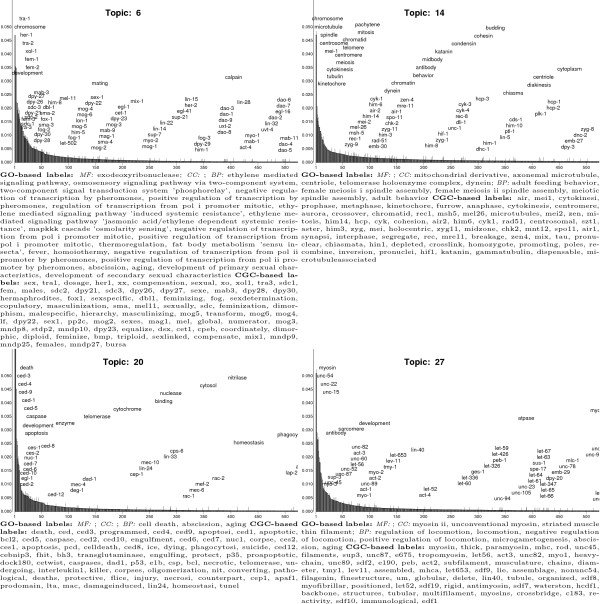
Results for four illustrative latent topics specified by a 50-topic CGC LDA model estimated from a corpus with 5,225 documents and a vocabulary of 28,971 words. Each panel shows results for a particular topic. The *y*-axis of the graph is topic-specific word probability (*β*_*kv*_)and words are arranged along the *x*-axis according to this likelihood. Only the 500 topic annotation words are plotted since the remaining words in the vocabulary have negligible probabilities. The words displayed explicitly are unigrams in the CGC vocabulary, including the names of *C. elegans *genes, and GO terms. The position of a word along the *x*-axis represents its rank; the staggering of words along the *y*-axis is not significant and is designed only to improve legibility. The graph legend lists two types of automatically-generated topic labels. CGC-based topic labels are a subset of the 50 × 500 topic annotation words that are unique to a topic and are words from the CGC vocabulary; these labels are ordered according to decreasing *β*_*kv *_values. GO-based topic labels are the parents and grandparents GO terms of GO terms that are also topic annotation words. Only GO terms that occur four or more times are given and are listed in decreasing frequency (MF: molecular function; CC: cellular component, BP: biological process). A CGC-based label is unique to a topic whereas a GO-based label can be applied to one or more topic.

Examination of the automatically generated CGC- and GO-based labels suggests that LDA topics capture meaningful and coherent facets of the molecular, cellular, and behavioral biology of *C. elegans*. Figure 6 shows results for four selected CGC topics (the results for all 50 topics are available in Additional file 1). The hidden factors permeating the CGC corpus include one pertaining to sexual reproduction (Topic 6), chromosome structure and function (Topic 14), cell death (Topic 20) and locomotion (Topic 27).

**Figure 7:**
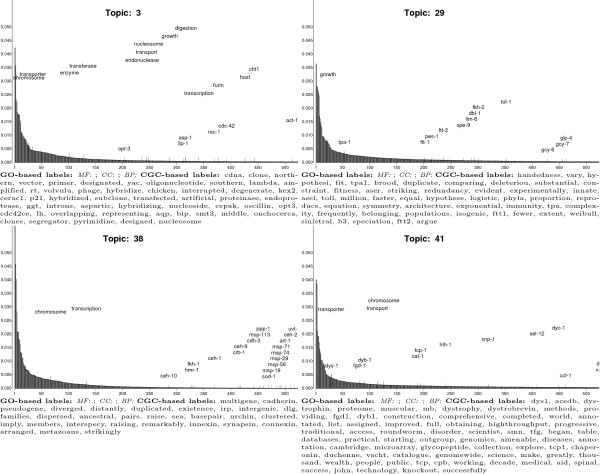
CGC LDA topics that have no GO-based topic labels and capture hidden factors in the CGC corpus that pertain to the practical aspects of investigating biological mechanisms and processes. Topics are represented in the same manner as in Figure 6.

Ontology-based topic labels derived from structured knowledge for domains other than molecular and cellular biology are needed to clarify the nature of some CGC hidden factors. Figure 7 shows topics that have CGG- but no GO-based labels. Inspection of the CGC-based labels for Topics 3, 29, 38, and 41 suggest the presence of hidden factors that are concerned with scientific protocols and procedures that are independent of any biological question, and that allude to evolution.

### Interrogation of LDA topics provides insights into genes influencing life span

A guilt-by-association approach was devised to identify genes that may be involved in a phenomenon of interest and the procedure illustrated using genes implicated in modifying life span. A "gene word" is a word in the 28,971 word CGC vocabulary that corresponds to the name of a *C. elegans *gene. CGC-based topic labels are the ≤ 500 words in the vocabulary that best characterize a topic. If a number of the topic labels are gene words and most of the genes are known to be associated with a specific phenomenon, then the other gene words can be equated with genes likely to be involved in the same phenomenon. One factor influencing the biological insights that can be derived from this approach is the human curation component of the process used to create the CGC Bibliography, *i.e*., the individual who defined the set of genes in the Genes record believed to be discussed in the Abstract record. In addition to limitations in the data used to estimate a statistical model of text, the LDA remains a model based on the simple bag of words representation of a document. While this LDA-based approach is not an automated method for formulating sophisticated and detailed hypotheses, it does highlight how a model that ignores syntax and semantics can organize information in a manner that provides a user the ability to exploit their background knowledge and enhance understanding of the subject in hand.

**Figure 8:**
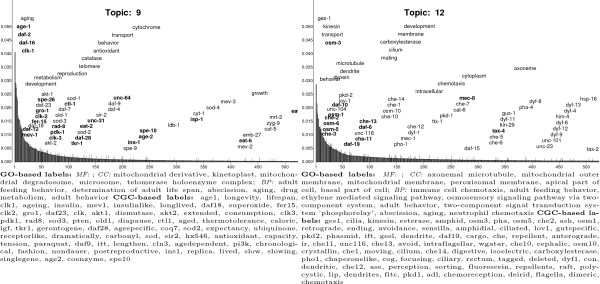
The two CGC LDA topics with the greatest numbers of aging-related gene words (CGC-based topic labels corresponding to the names of genes implicated in modifying life span). Each topic is represented the same manner as Figure 6. In the graph, words in bold are the gerontogenes listed in Table 1.

Table 1 lists the names of genes known to extend or shorten life span and designated aging-related gene words. Inspection of the two CGC LDA topics with the greatest number of CGC-based labels that are aging-related gene words suggests that *akt-1, akt-2 *and *ges-1 *may be associated with aging. Figure 8 shows these two topics, Topic 9 and Topic 12. In decreasing topic-specific word probability and with genes listed in Table 1 in bold, the Topic 9 CGC-based labels that are gene words are **age-1, clk-1, mev-1**, daf-18, **fer-15, clk-2, gro-1**, daf-23, akt-1, akt-2, **clk-3, pdk-1, rad-8**, sod-3, old-1, **ctl-1, tkr-1, daf-28**, sod-2, sir-2, daf-9, cln-3, ins-1, **age-2**, and **spe-10**. The genes depicted in a normal font have properties similar to known life span modifying genes such as dauer (daf) phenotypes (Table 1). For example, the Wormbase [25] annotations for *akt-1 *and *akt-2 *include "protein serine/threonine kinase" and "inhibition of both akt-1 and akt-2 leads to dauer-constitutive phenotype."

**Table 1:** *C. elegans *genes known to extend or shorten life span. The list is taken from the Genes database of SAGEKE http://www.sageke.org

Life span extension	
Hsp-6	heat shock 70 protein
age-1	inositol/phosphatidylinositol kinase; signal transduction
age-2	AGEing alteration
che-2	G-beta-repeats
che-3	microtubule motor, dynein ATPase; microtubule-based movement
che-11	abnormal CHEmotaxis
che-13	abnormal CHEmotaxis
clk-1	ubiquinone biosynthesis
clk- 2	CLocK (biological timing) abnormality
clk-3	CLocK (biological timing) abnormality
daf-2	transmembrane receptor protein tyrosine kinase; phosphorylation, hydrogen transport, signalling
daf-6	abnormal DAuer Formation
daf-10	abnormal DAuer Formation
daf-12	steroid hormone receptor; transcription regulation
daf-19	DNA binding transcription factor; transcription regulation
daf-28	abnormal DAuer Formation
eat-1	EATing: abnormal pharyngeal pumping
eat-2	EATing: abnormal pharyngeal pumping
eat-3	EATing: abnormal pharyngeal pumping
eat-6	Na^+^/K^+ ^ATPase *α *subunit; cation transport, metabolism
eat-13	EATing: abnormal pharyngeal pumping
glp-1	calcium ion binding; cell differentiation
gro-1	tRNA isopentenyltransferase; tRNA processing
ins-1	INSulin related
ins-18	insulin-like growth factor I like; hormone
isp-1	ubiquinol-cytochrome c reductase, Rieske iron-sulfur protein; electron transport
mec-8	nucleic acid binding; mechanosensory
mes-1	protein tyrosine kinase
osm-1	OSMotic avoidance abnormal
osm-3	kinesin
osm-5	aspartic-type endopeptidase; proteolysis and peptidolysis
osm-6	N-acetyllactosamine synthase
pdk-1	protein serine/threonine kinase
pgl-1	P GranuLe abnormality
rad-8	RADiation sensitivity abnormal/yeast RAD-related
sir-2.1	DNA binding; transcription regulation, chromatin silencing
spe-10	defective SPErmatogenesis
spe-26	MIPP repeats; defective SPErmatogenesis
tax-4	Cyclic-nucleotide-gated olfactory channel; potassium transport
tkr-1	G protein coupled receptor; signalling
unc-4	homeobox protein (otd subfamily); transcription regulation
unc-13	intracellular signaling cascade
unc-26	inositol/phosphatidylinositol phosphatase
unc-31	PH (pleckstrin homology) domain
unc-32	TJ6/proton pump
unc-64	syntaxin
unc-76	UNCoordinated

Shortened life span	

ctl-1	CaTaLase
daf-16	transcription factor
eat-7	EATing: abnormal pharyngeal pumping
fer-15	FERtilization defective (abnormal sperm)
mev-1	Succinate dehydrogenase cytochrome b chain; electron transport, tricarboxylic acid cycle

The mechanisms of action of putative gerontogenes suggested by different topics may not be identical. For Topic 12, the CGC-based labels that are gene words are ges-1, **osm-3, osm-5, che-2, osm-1**, lov-1, pkd-2, **daf-19, che-11**, unc-116, **che-13**, che-10, osm-10, che-1, che-14, pho-1, dyf-1, che-12, and pkd-1. *ges-1 *is a gut-specific carboxylesterase, a molecular function not ascribed by Wormbase to life span modifying genes. Since many topic labels are associated with the osm phenotype, osmoregulation may be a feature that differentiates Topic 12 aging-related genes from those of Topic 9.

### Exhibition of multiple latent topics by an LDA document reflects the complexity of issues discussed in documents

By virtue of its superior generalization performance and retrieval ability, an LDA model of the CGC corpus is a better statistical model of text than a mixture of unigrams model. A distinct advantage of LDA is that although both models are generative, an LDA document is the manifestation of many topics whereas a mixture of unigrams document is the product of only one topic. The subject matter of (biological) documents is rarely limited to a single area so the benefit of a CGC LDA is that words in a single document could come from, for example, a combination of Topic 6 (sexual reproduction) and Topic 14 (chromosome structure and function). The mixing of LDA topics in a CGC item was investigated by examining the document-specific, word-level parameter *φ *(Figure 2). The variational posterior topic probability *φ*_*n*_(*z*_*n*_*= k*) indicates the extent to which the *n*th word is associated with the *k*th topic. A value that is both large and significant is an indicator of the topic most likely to have generated the word.

**Figure 9:**
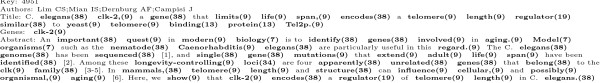
The LDA topics most associated with words in the CGC item shown in Figure 1. A word is identified with the topic *k *given in parenthesis when the document-specific, variational posterior topic probability exceeds a threshold, *φ*_*n*_(*z*_*n*_*= k*) > 0.9. As illustrated by "telomere (9)", identical words within a document are generated by the same topic. Note that only the Title, Genes and Abstract records were concatenated and processed to generate the bag-of-words document used to estimate the LDA.

The CGC item shown in Figure 1 is primarily a mixture of two topics. Figure 9 shows the topics most likely to have produced words in the document discussing the life span modifying *clk-2 *gene (Table 1). Of the assigned words, 34 have posterior probabilities peaked on the aging-related Topic 9 (Figure 8) and 23 on the general purpose Topic 38 (Figure 7). Three words are allocated to Topic 7, two to Topic 19, two to Topic 13, and one to Topic 34.

### Utility of LDA in formulating hypotheses: insights into clk-2 function

**Figure 10:**
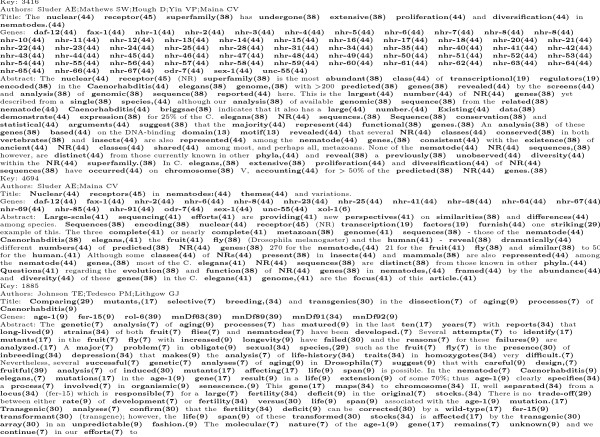
CGC homologs of the *clk-2 *item shown in Figure 1. When this *clk-2 *item is used as the query document, the three items shown have the largest topic-space pairwise similarity scores, (*q*, *t*). The documents are depicted in the same format as Figure 9. As illustrated by "elegans(38)" and "elegans(41)" in the first and second top-ranked documents, identical words may be attributed to different topics in different documents.

**Figure 11:**
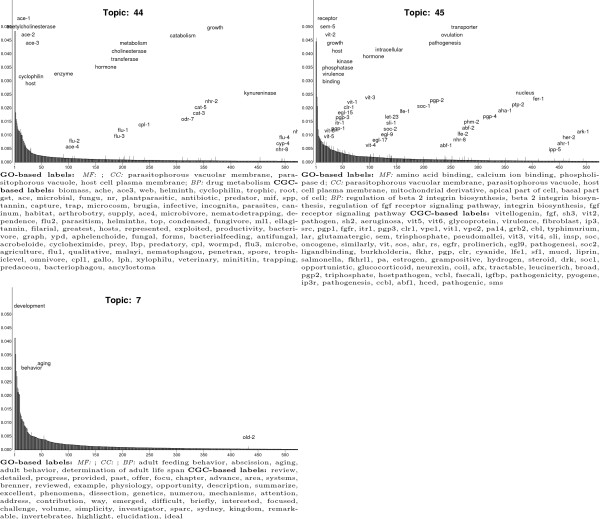
The CGC LDA topics most associated with words in the document homologs of the *clk-2 *query shown in Figure 10.

Searching a document database to identify homologs of a query document yields insights that can complement those obtained from sequence-, structure- and function-based analysis of genes and proteins. Prior studies of *clk-2 *revealed that it encodes a sequence homolog of Tel2, a protein required for normal telomere length regulation in yeast (reviewed in [26]). To enhance knowledge of how *clk-2 *might influence life span, homologs of a document discussing *clk-2 *(Figure 1) were identified by computing the topic-space pairwise similarity score between this query *q *and every CGC document *t *(Equations 2 and 3). Although a given gene may appear in the Genes record of many CGC items, the results described below are based on analysis of document homologs of the single CGC item shown in Figure 1. Figure 10 shows the three most related items, CGC documents with the three largest (*q*, *t*) values. Figure 11 shows the three topics most associated with them. The third best homolog is relatively uninformative: the text indicates a general review of aging mutants and Topic 7 labels are general words pertaining to life span.

LDA-based analysis leads to the hypothesis that *clk-2 *may have a role in coordinating signals between the outside and inside of cells. Since the top two topic-space document homologs discuss nuclear receptors, *clk-2 *may have a direct or indirect involvement in receptor biology. Topics 44 and 45 include the GO-derived labels "host cell plasma membrane," "regulation of fgf receptor signaling pathway" and "regulation of beta 2 integrin biosynthesis." Circumstantial evidence supports a possible role for clk-2 in signal transduction and tissue biology. FGF-2 regulates telomerase activity in human endothelial cells [27]. Integrins are cell surface receptors important in communication between the extracellular environment and the nucleus [28]. The suggestion that *clk-2 *may influence telomere length via a mechanism not involving direct physical association with telomeres is plausible since a recent genome-wide screen for *Saccharomyces cerevisiae *deletion mutants that affect telomere length identified genes with very diverse functions (overrepresented categories included DNA and RNA metabolism, chromatin modification, and vacuolar traffic) [29]. Recent experimental results support a connection between vacuolar protein-sorting genes and telomere length homeostasis [30].

## Discussion

This study demonstrates how a specific statistical IR model, an LDA model, can be employed to infer the hidden factors permeating a biomedical text corpus and exploited to synthesize and organize information about complex biological phenomena. The results indicate that despite being estimated from a simple bag of words representation of items in the CGC Bibliography, the intra-document statistical structure captured by an LDA model is sufficient for the model to be used to enhance understanding of *C. elegans *biology. For example, analysis of the corpus-, document- and word-level parameters of a trained LDA model enabled the exploration and creation of hypotheses about known and putative nematode aging-related genes.

The CGC corpus studied here had *M *= 5, 225 documents and a *V *= 28, 971 word vocabulary. Estimating a 50-topic LDA model from such training data took 3 hours on a Macintosh Powerbook G4. It should be straightforward to estimate a model for larger corpora such as MEDLINE where the number of documents is many orders of magnitude greater (*M *~ 10^7^) and the vocabulary size is only one order of magnitude larger (*V *~ 10^5^). In estimating an LDA, the computational bottleneck is the variational E-step, *i.e*., computing the posterior topic Dirichlet distribution for each document. Fortunately, this procedure can be parallelized because given a model, the posterior for each document can be assessed independently. Thus, it is feasible for the techniques described in this study to be applied to other corpora and to address questions other than life span modification.

From an applications perspective, it is possible to envisage a scenario involving the creation of a library of LDA models where each constituent model was estimated from a user- and/or computationally-defined corpus of documents focused on a specific area such as "aging," "cancer," "yeast biology," "response to stress," "antibiotics," "HIV," "Parkinson's disease," "kinases," and so on. A query document would be compared to each subject-specific LDA in the collection as opposed to a single model as described here. This approach seeks to mirror a common strategy in sequence analysis whereby a query sequence is rated against a library of hidden Markov models (HMMs) [31] estimated for domains of interest, for example, the Pfam database of protein families [32]. Since seaching a database of probabilistic models of proteins in order to identify "remote" sequence homologs is known to be effective, an analogous approach could prove useful in retrieving distant document homologs.

The ideas discussed in this work can be extended and improved in a variety of ways. Currently, the number of latent topics in an LDA model *K *is a user-defined parameter but recent research has examined the task of choosing *K *[33]. In particular, if the LDA is augmented with a non-parametric Bayesian prior known as a hierarchical Dirichlet process, both topic probabilities and the number of topics can be estimated from data. Under the hierarchical Dirichlet process prior, the number of topics grows as data are added to a collection [44]. The significance and practical importance of this feature is that when modeling scientific documents, the nature and size of the corpus is evolving constantly meaning that discovering topics is an ongoing task. Further studies are required to devise rigorous methods for determining a set of words best able to characterize a topic: the current approach is somewhat arbitary in that corpus-based topic labels were defined as the 500 words in CGC vocabulary with the highest values for the likelihood of the word given the topic.

In the LDA model, topics represent entities that are presumed to permeate a corpus and these latent variables are to be inferred by statistical analysis. These implicit concepts are assumed to be equally related to one another, *i.e*., the topics are not organized in any way and form a "flat structure." It seems reasonble to believe that, for example, topics embodying the concepts of "DNA repair" and "chromosome structure and function" should be more related to each other than to a topic focused on "locomotion." Thus, a model in which topics were themselves arranged as a hierarchy could prove useful and inspection of the relationship(s) between topics might be informative. The current LDA model represents a model for a particular instantiation of a corpus. It might be interesting to formulate dynamic models of corpora which sought to capture how a collection such as the CGC or MEDLINE changes over time. In addition to theoretical studies of their properties and behavior, such models might be useful not only for biomedical researchers and clinicians, but also policy makers, historians, and sociologists interested in the evolution of biology and its disciplines.

Statistical IR models are applicable not only to "traditional" document collections, but also to genome-scale biological data. In one example of such an LDA model, "documents" could correspond to genes, "topics" to regulatory networks and "words" to motifs. Since LDA does not require mutually exclusive clustering, a given gene can participate in several networks and a given motif can appear in several genes. Merging this type of analysis with that described here would result in evidential support for the functional behavior and role of a gene being derived both from primary biological data and from corpus-based analysis. This capacity to fuse support from disparate sources has been illustrated using a variant of LDA known as "correspondence LDA" and in the context of automated image annotation [23].

## Conclusion

LDA is a special case of the family of probabilistic graphical models. This family includes a wide variety of other models that have proved useful in biology such as HMMs, phylogenetic trees, and pedigrees [34]. The graphical model formalism allows such graphical components to be combined into heterogenous, large-scale statistical models that integrate evidence from multiple sources. Doing so would yield a system for facilitating the formulation of ideas that could be interrogated and verified experimentally.

## Methods

### CGC document corpus

The CGC Bibliography (October 2002 release) was downloaded [35] and included abstracts from the published literature, "worm meetings," and the "Worm Breeder's Gazette." Each CGC item is a series of defined records (Key, Medline, Authors, Title, Citation, Type, Genes, Abstract) and associated free-text (see Figure 1 for an exemplar). In an item, the Genes record associated with an Abstract record was added by a curator and so reflects the personnel interest(s) and background of the individual, *i.e*., the list of genes discussed in the abstract was not derived in a systematic, automated manner and according to a fixed scheme and/or philosophy. For each item, the Genes, Title, and Abstract records were concatenated. The resultant text was tokenized by partitioning on the basis of white spaces and punctuation. Variants of the same word were stemmed to produce a single word by removing the suffixes s, ss, ies and sses. A word was discarded if it was in a set of 619 generic stop words that included ii, iii, iv, iv, a, a., yourselves, z, and zero. The ensuing text was not tagged, *i.e*., words were not annotated in terms of syntax (parts of speech) or semantics (pre-defined class such as "gene name").

A CGC document is the bag of words (vector space representation) obtained after tokenizing, stemming, and removing suffixes from a CGC item. The CGC vocabulary is the non-redundant set of discrete objects produced after application of the preceeding processing steps to all items in the corpus. Words in this vocabulary are unigrams because phrases such as "DNA repair" are considered to be two objects rather than one. The final CGC corpus had *M *= 5, 225 documents and a vocabulary of *V *= 28, 971 words. The shortest and longest documents had *N *= 9 and *N *= 261 words respectively. It should be noted that words originating from the Genes record were not marked in any way so neither the vocabulary constructed for the corpus nor the bag of words representation of a particular document contained explicit knowledge of, or information about the curation effort.

### Latent Dirichlet Allocation (LDA) model

A detailed description of the LDA model and attendant algorithms can be found elsewhere [22,36]. Figure 2 gives a graphical model representation of LDA. Consider a corpus of *M *documents, {**w**_1_, ..., **w**_*M*_}, based on a vocabulary of words indexed by [1, ..., *V*]. A document is a sequence of *N *words, **w **= [*w*_1_, ..., *w*_*N*_]. Each word in a document is represented by a *V*-dimensional unit-basis vector in which only one component is equal to one, *i.e*., a word that is the *v*th word in the vocabulary is described by a vector where *w*^*v *^= 1 and *w*^*u *^= 0 for all *u ≠ v*.

LDA generates a document according to the following process,

1. Choose a point *θ *from a Dirichlet distribution parameterized by *α*: *θ *~ Dirichlet (*α*).

2. For each word *w*_*n *_in turn,

(a) Choose a topic *z*_*n *_from a multinomial distribution parameterized by *θ*: *z*_*n *_~ Multinomial(*θ*).

(b) Choose a word *w*_*n *_from the multinomial distribution associated with the selected topic and parameterized by : *w*_*n *_~ Multinomial ().

The parameters of a *K*-iopic LDA are the Dirichlet parameter, *α*, and the topic-word matrix, *β*. *α *is a *K*-dimensional Dirichlet parameter that determines the distribution over topic proportions, *α *= [*α*_1_, ..., *α*_*K*_] where *α*_*k*_*> *0. *β *is a *K *× *V *matrix that determines the likelihood of the *v*th word in the vocabulary given the *k*th topic, *β*_*kv*_*= p*(*w*^*v *^= 1 | *z*^*k *^= 1). The topic-specific word distribution β_k_ is the *k*th row of *β*. The document-specific topic proportions *θ *lie in the (*K *- 1)-dimensional simplex, *θ *= [*θ*_1_, ..., *θ*_*k*_] where *θ*_*k *_= *p*(*z*_*^k^*_= *1 *| *θ*), *θ*_*k*_*> *0, and .

The likelihood of an LDA document is obtained by marginalizing over the latent variables,

The probability of a corpus is the product of the marginal probabilites of single documents.

#### Inference and parameter estimation

Estimating the parameters of an LDA from data and calculating the probability of a document both involve inference or computing the posterior distribution of latent variables given a document,

The denominator of this expression, the likelihood of a document, cannot be computed exactly because of the coupling between *θ *and **z **(left, Figure 2). Instead, approximate inference methods are required for the LDA model. Such methods include the convexity-based variational approach used here [22], Markov chain Monte Carlo sampling [37] and expectation propagation [38].

Variational inference approximates the posterior by finding a lower bound on the likelihood of a document [22]. The family of graphical models obtained by uncoupling *θ *and **z **(right, Figure 2) is characterized by the following variational distribution,

where the Dirichlet parameter *γ *and the multinomial parameters {*φ*_1_, ..., *φ*_*N*_} are the free variational parameters for document **w**. For the *n*th word, *φ*_*n*_(*z*_*n*_* = k*) is the (variational) posterior topic probability for the *kth *topic. Optimal document-specific values can be found by minimizing the Kullback-Leibler (KL) divergence between the variational distribution and the true posterior,

The variational Dirichlet parameter γ* (**w**) provides a representation of the document in the topic simplex. The variational multinomial parameters φ*(**w**) = {, ..., } approximate the true, but intractable distributions *p*(*z*_*n*_|**w**).

Estimating an LDA from data involves finding the Dirichlet parameter α* and topic-word matrix β* which maximize the log marginal likelihood of a corpus. A variational Expectation-Maximization procedure results in parameter estimates that are a (possibly local) maximum of a lower bound of the log marginal likelihood. This alternates between maximizing a lower bound with respect to the variational parameters for each document, and maximizing the lower bound with respect to the model parameters.

### An LDA-based measure of pairwise document similarity

The transformation of bags of words into bags of topics by LDA provides a means to address the task of searching a corpus to retrieve similar and/or relevant items. Word-space representations of documents (high-dimensional, variable length, vectors of discrete-valued features) are converted into topic-space representations (low-dimensional, fixed length, vectors of real-valued features). In particular, the variational posterior Dirichlet parameter γ* (**w**) indicates the degree to which each of the *K *topics is referenced by document **w**.

A new measure of pairwise document similarity was formulated using the Dirichlet probability distribution specified by the document-level LDA parameter *γ *(Figure 2). Recall that the variational posterior Dirichlet distribution for document *d *is Dirichlet(*γ*_*d*_) where  and  denotes the extent to which document *d *refers to the *k*th topic. A random variable *θ *drawn from Dirichlet(*γ*_*d*_) has the probability density

where , ,  > 1, and Γ(·) is the Gamma function. Given two Dirichlet densities, the KL divergence is given by

The similarity between two documents *i *and *j *can be quantified by computing the KL divergence between their corresponding Dirichlet distributions as follows

where *γ*_*i *_and *γ*_*j *_are the parameters for the two documents. ψ (·) is the digamma function and arises when taking expectations of log *θ*.

Let (*q*, *t*) be the topic-space pairwise similarity score between a query document *q *and a target document *t*. Here, this score is defined as the symmetrized KL divergence between the variational posterior Dirichlet distributions,

where the component KL terms are computed using Equation 2.

An alternative definition of the topic-space pairwise similarity score is the Jensen-Shannon divergence

where *γ*_*x *_= (*γ*_*q *_+ *γ*_*t*_)/2 so that *γ*_*x^k^*_is the average degree to which documents *q *and *t *refer to topic *k*.

Given a database of *D *documents, *t*_1_, ..., *t*_*D*_, the task of retrieving documents related to a query *q *was addressed by computing the score between *q *and each document in the collection, (*q*, *t*_1_), ..., (*q*, *t*_*D*_) (*cf*. searching a protein or nucleic acid sequence database to find homologous sequences). For simplicity, a "homolog" of a document *q *is a CGC document *t*_*d *_that has a high topic-space pairwise similarity score (*q*, *t*_*d*_).

### Mixture of unigrams model and unigram model

Figure 3 shows graphical model representations of two existing models of text. Like the graphical model representation of the LDA model (left, Figure 2), the open circles represent parameters for the mixture of unigrams model (mixing weights *θ*; word distribution *β*_*k*_)and unigram model (word distribution *β*).

The unigram model contains no latent variables and each word in every document is assumed to have been drawn from the same multinomial distribution. Denoting this word distribution as *β*, the likelihood of a unigram document is

The mixture of unigrams model [39] assumes that each document is generated by first choosing one of *K *topics, and then drawing words independently, conditioned on that topic. As in LDA, the *K *multinomial distributions represent an underlying semantic structure in the corpus but a mixture of unigrams document is a manifestation of only one of these topics. Denoting the mixing weights as *θ *and the word distributions as *β*, the likelihood of a mixture of unigrams document is

The LDA model builds upon the mixture of unigrams in that documents are able to manifest multiple topics. Overall, the estimated LDA word distributions are better reflections of the underlying topics in a corpus, particularly in light of heterogenous documents.

### Assessment of statistical models of text

#### Perplexity: generalization performance

The performance of a statistical model on unseen data was evaluated by computing the perplexity of a test set of *J *documents not used to estimate the model,

where *N*_*j *_is the number of words in test document **w**_*j*_. The perplexity is equivalent to the inverse of the geometric mean per-word likelihood and a lower score indicates better generalization performance.

The three different bag of words models described above were assessed by determining their respective perplexity on the same set of test documents. The likelihood of a test document *p*(**w**_*j*_) was computed using Equation 1 for an LDA model (left, Figure 2), Equation 4 for a unigram model (left, Figure 3), and Equation 5 for a mixture of unigrams model (right, Figure 3). A model was trained using 90% of the CGC corpus (4,700 documents) and the remaining 10% (*J *= 525) used to compute perplexity. A single unigram model was estimated and evaluated whereas LDA and mixture of unigram models with varying numbers of latent topics were estimated and evaluated (*K *= 5,10, 20, 50,100).

#### Precision/recall (PR) curves and F1 measure: retrieval capability

The ability of a statistical model to retrieve a set of related documents was evaluated using a language modeling approach [20,40]. Let *N*_*D *_be the number of "relevant" documents in a collection of *D *documents, documents that are related according to some criterion. Let *N*_*P *_be the number of these relevant documents that are present in a list of *P *of these documents (*P *≤ *D*). Precision, , is the fraction of documents in the list that are relevant and is defined as *N*_*P*_/*P*. Recall, , is the fraction of relevant documents in the list and is defined as *N*_*P*_/*N*_*D *_( = 1 when the list of documents and collection of documents are identical, *N*_*P *_= *N*_*D*_). The *F*1 measure is the harmonic mean of these two statistics,

Given a ranking of the documents in the collection, *D *lists of documents are produced by selecting the top P-ranked documents where *P *= 1, ... ,*D*. For the *d*th list, the precision, _*d*_, recall, _*d*_, and F1 measure, *F*1_*d *_are computed. The average of the *D *Fl measures for the collection, *F*_1_, ..., *F*_*D*_, is calculated.

Three different models were assessed by determining their respective retrieval capability on the same set of relevant documents present in the collection of *D *= 5, 225 CGC documents (an LDA model, a mixture of unigrams model, and a model which ranks documents randomly). The common subject matter of the relevant documents was genes implicated in extending or shortening the life span of *C. elegans*. This set of *N*_*D *_= 842 aging-related documents was created by identifying CGC items in which the Genes record (Figure 1) refers to one or more of the genes listed in Table 1 that are known to modify life span.

A *K *= 50 topic LDA model was trained using all *D *CGC documents. Given this model, the symmetrized KL divergence between an aging-related document posterior (*γ*_*q*_) and every CGC document posterior (*γ*_*t*_) was computed using Equations 2 and 3. These *D *topic-space pairwise similarity scores, (*q*, *t*_1_), ..., (*q*, *t*_*D*_), were used to rank the CGC documents from most (highest score) to least similar. This process was repeated for each relevant document in turn, *q*_1_, ..., , resulting in *N*_*D *_rankings of the CGC documents. Let *P*_*i *_be the *P *top-ranked CGC documents for query *q*_*i*_. The number of known aging-related documents in this list was determined and used to calculate precision and recall. The average precision (average recall) is the mean of the precision (recall) value computed for each list *P*_1_,..., . A series of such average precision and average recall values were computed by varying the number of top-ranked documents used to create lists, *P *= 1, ..., *D*. A PR curve was constructed from these *D *pairs of average precision and average recall values. The average F1 measure is the mean of the F1 measure computed for each of the *N*_*D *_relevant documents.

A *K *= 50 topic mixture of unigrams model was trained using all *D *CGC documents. Given this model, the symmetrized KL divergence between an aging-related document **w**_*q *_and a CGC document **w**_*t *_was computed using

In the mixture model, the divergence is between posteriors of the single latent topic rather than the vector of latent topic proportions as is the case with LDA. The component KL term between documents **w**_*i *_and **w**_*j *_is calculated using

where *p*(*z*|**w**_*i*_) and *p*(*z*|**w**_*j*_)are the posterior probabilities of class *z *given documents **w**_*i *_and **w**_*j *_respectively. The mixture model pairwise similarity scores, (**w**_*q*_, ),..., (**w**_*q*_, ) were used to rank the CGC documents from most (highest score) to least similar and the procedure repeated for each of the *N*_*D *_relevant documents. The resultant *N*_*D *_rankings were used to construct a PR curve and calculate average F1 as described above for the LDA model.

A random model was created by randomly ordering the *D *CGC documents and repeating this process *N*_*D *_times. These *N*_*D *_"rankings" of the *D *documents in the collection were used to construct a PR curve and calculate averate F1 as described above for the LDA model.

## Authors' contributions

DMB, MIJ, and ISM conceived and designed the study, analyzed the results, and wrote the manuscript. DMB formulated algorithms, wrote the software, and performed the experiments. KF wrote the software needed for the Gene Ontology-based analysis of LDA topics. All authors approved the manuscript.

## Supplementary Material

Additional File 1 Results for each of the LDA topics specified by a 50-topic model estimated from a corpus of 5,225 documents and a 28,971 word vocabulary. Click here for file
