# Supplementary material for: Statistical modeling of biomedical corpora: mining the Caenorhabditis Genetic Center Bibliography for genes related to life span
Source: BMC Bioinformatics. 2006 May 8;7:250. doi: 10.1186/1471-2105-7-250 (PMC1533868; doi:10.1186/1471-2105-7-250)
Supplement: Additional File 1 — Results for each of the LDA topics specified by a 50-topic model estimated from a corpus of 5,225 documents and a 28,971 word vocabulary. [file 1471-2105-7-250-S1.pdf]

Statistical modeling of biomedical corpora: mining the Caenorhabditis Genetic Center Bibliography for genes related to life span

D.M. Blei*[1], K.M. Franks[2], M.I. Jordan*[1,3] and I.S. Mian*[2]

[1]Computer Science Department, Princeton University, Princeton, New Jersey 08540 USA
[2]Life Sciences Division, Lawrence Berkeley National Laboratory, Berkeley, California 94720-8265, USA
[3]Department of Statistics, University of California Berkeley, Berkeley, California 94720, USA

Email: D.M. Blei*- blei@cs.princeton.edu; M.I. Jordan*- jordan@cs.berkeley.edu; I.S. Mian - smian@lbl.gov;

*Corresponding author

Additional Files

Additional File 1: `bmc_lda_supplement.pdf`

Results for each of the 50 latent topics specified by a 50-topic LDA model estimated from a corpus of 5,225 documents and a 28,971 word vocabulary. Each panel shows results for a particular topic. The y-axis of the graph is topic-specific word probability (β_{kv}) and words are arranged along the x-axis according to this likelihood. Only the 500 topic annotation words are plotted since the remaining words in the vocabulary have negligible probabilities. The words displayed explicitly are unigrams in the CGC vocabulary and include the names of *C. elegans* genes and GO terms. The position of a word along the x-axis represents its rank; the staggering of words along the y-axis is not significant and is designed only to improve legibility. Gene names in bold denote genes implicated in modifying life span and listed in Table 1 of the manuscript. The graph legend lists two types of automatically-generated topic labels. CGC-based topic labels are a subset of the 50×500 topic annotation words that are unique to a topic and are words from the CGC vocabulary; these labels are ordered according to decreasing β_{kv} values. GO-based topic labels are the children and grandchildren GO terms of GO terms that are also topic annotation words. Only GO terms that occur four or more times are given and are listed in decreasing frequency (MF: molecular function; CC: cellular component, BP: biological process). A CGC-based label is unique to a topic whereas a GO-based label can be applied to one or more topic.

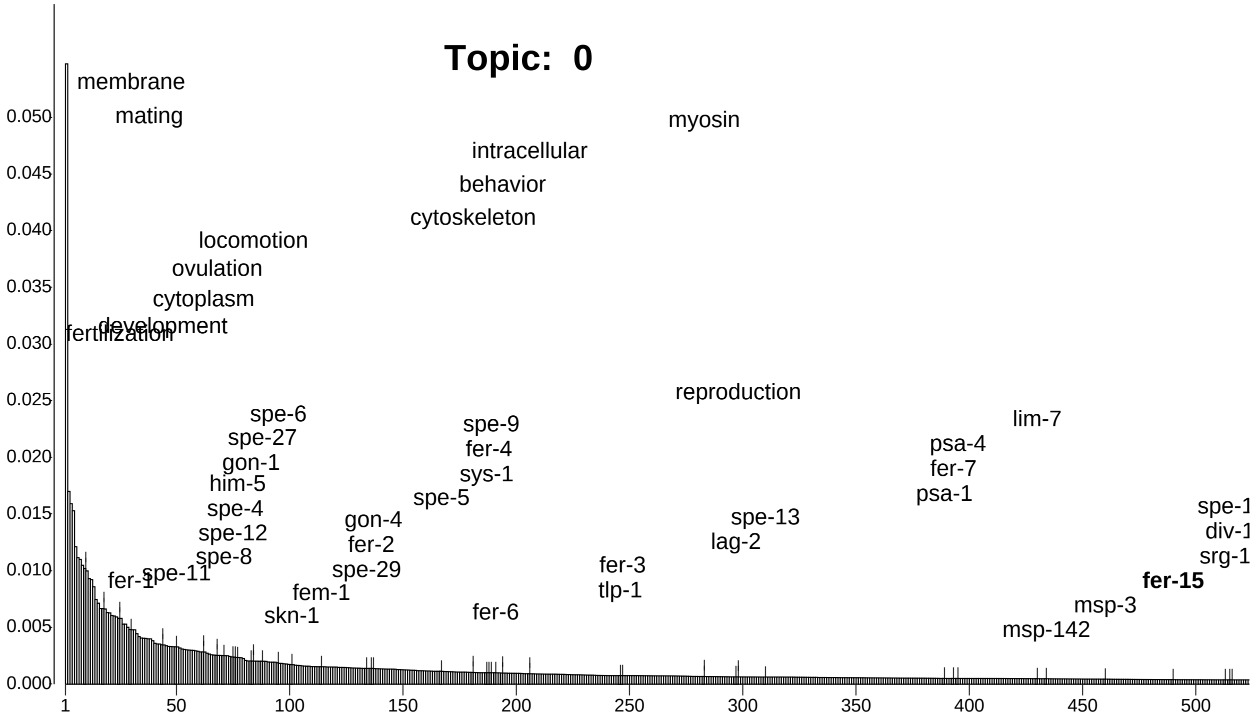

GO-based labels: *MF:* ; *CC:* mitochondrial outer membrane, mitochondrial membrane, peroxisomal membrane, cytoplasmic chromosome, intermediate filament cytoskeleton, apical part of cell, basal part of cell; *BP:* hexose mediated signaling, adult feeding behavior, ethylene mediated signaling pathway, osmosensory signaling pathway via two-component system, abscission, aging, carbohydrate mediated signaling, regulation of locomotion, adult behavior, two-component signal transduction system 'phosphorelay' **CGC-based labels:** msp, spermatozoa, spermatid, pseudopod, motility, skn, fer1, crawling, flow, spermiogenesi, fertilized, spermatheca, spe11, spe, spermatocyte, amoeboid, gonadogenesi, competition, ovulation, spe8, spe12, spe4, proximal, gon1, spe27, crawl, spe6, membranou, spermspecific, spermathecal, fertilize, motile, leading, cytoskeletal, spe29, pseudopodial, mfs, fer2, gon4, mos, bzip, fer, monensin, mo, spe5, metalloprotease, seminal, sys1, fertilizationdefective, fer4, attached, fer6, foci, delayed, sessile, triethanolamine, spherical, edge, unfertilized, appearance, selffertilization, fluid, spermatozoan, competitiveness, spermatozoon, fer3, tlp1, rearward, precedence, filopodia, div, sealing

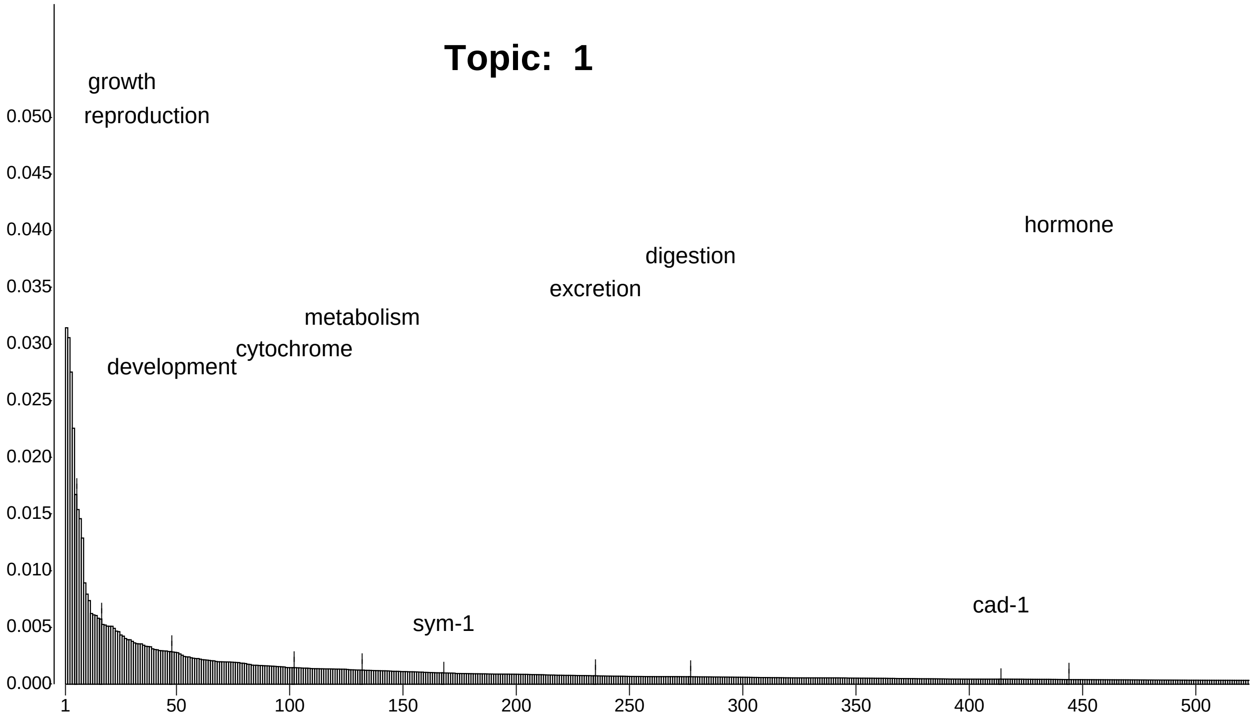

GO-based labels: *MF:* ; *CC:* ; *BP:* abscission, aging, drug metabolism **CGC-based labels:** axenic, media, liver, aceti, chemically, turbatrix, supplement, nutritional, dougherty, axenically, supplemented, proteinaceou, glycogen, glycerol, monoxenic, heme, chick, glucose, folic, nutrition, particulate, mitop, aminopterin, nigon, aqueou, redivivus, aphelenchu, radioactive, anaerobic, liter, rh, methionine, nitrogen, incubated, preliminary, fluorodeoxyuridine, sym1, cultures, culturing, mineral, hemin, haemin, suspension, utilization, incorporation, sialic, easily, reared, pure, supported, degenerative, cbmm, haem, synchrony, histidine, digested, excretion, concluded, plate, evaluated, lg

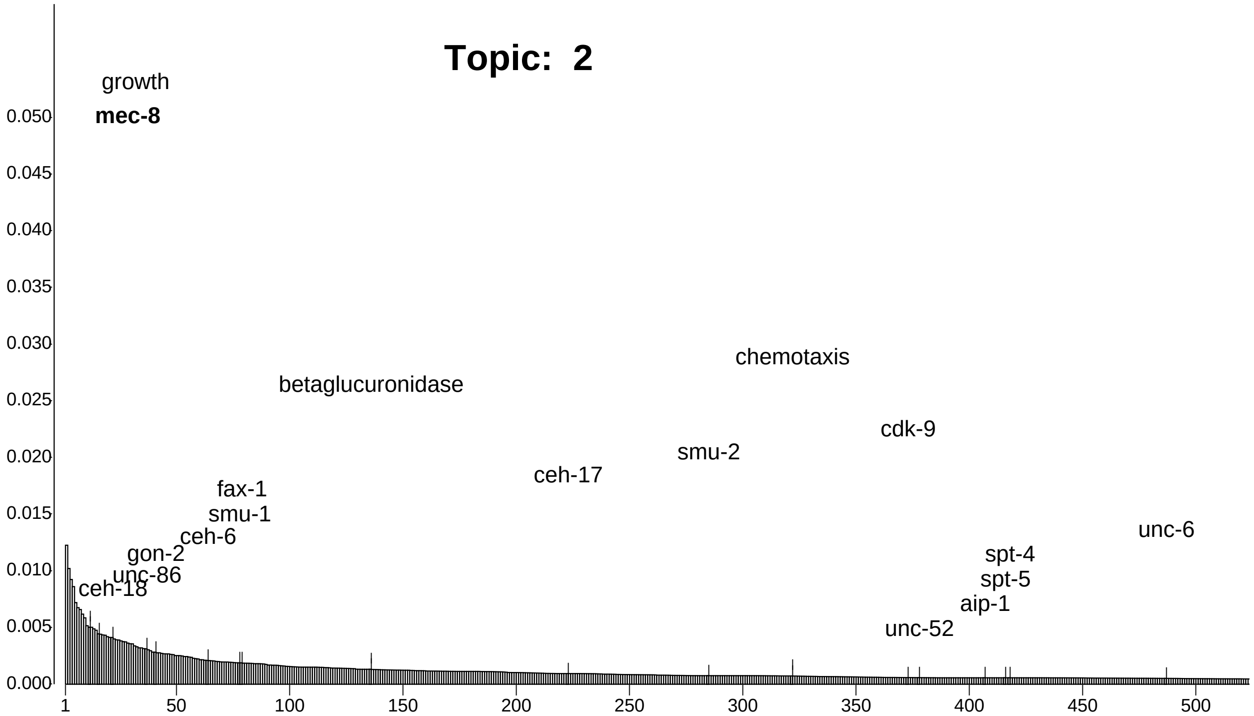

GO-based labels: *MF:* exodeoxyribonuclease; *CC:* ; *BP:* **CGC-based labels:** training, memory, image, longterm, humic, conditioning, retention, ceh18, pyrene, bioconcentration, hs, gon2, cold, measuring, three-dimensional, cr, dhp, gravity, doc, ceh6, thesauru, imaging, recorded, dissolved, smu1, fax1, machine, video, tefb, automatic, improve, precision, filtrate, fourdimensional, algorithm, pirouette, cecrmp, cooling, spt, simultaneously, optical, coefficient, robot, crmp, piwi, grd, betaglucuronidase, concept, dimension, nonneural, robust, statistical, spring, experiments, tube, gsc, hh, wrt, nom, ham, bap, bcf, retrieval, drift, digital, record, local, em, timelapse, sand, nonlinear, scanning, created, discrete, session, pit1, aceperl, ceh17, airap, vocabulary, benzo, simulated, nonspecific, intensity, wholemount, document, back, foraging, multiphoton, biologically, ground

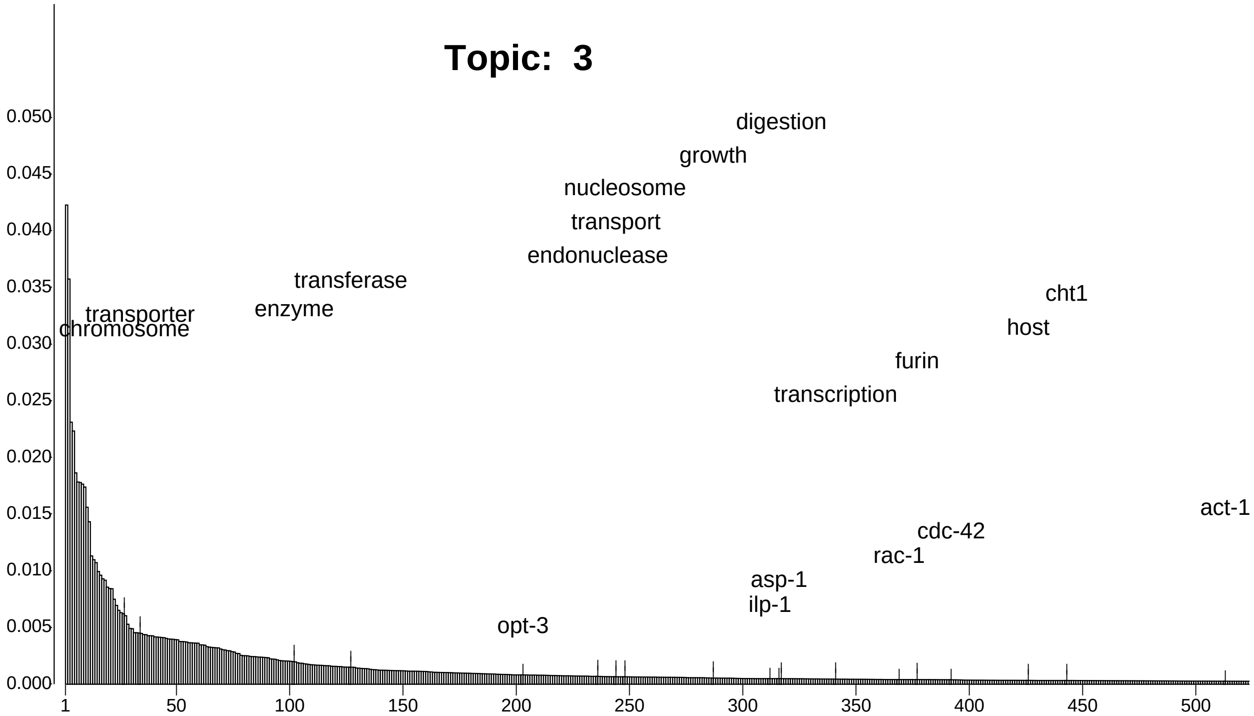

GO-based labels: *MF:* ; *CC:* ; *BP:* **CGC-based labels:** cdna, clone, northern, vector, primer, designated, yac, oligonucleotide, southern, lambda, amplified, rt, volvulu, phage, hybridize, chicken, interrupted, degenerate, kex2, cerac1, p21, hybridized, subclone, transfected, artificial, proteinase, endoprotease, ggt, introns, aspartic, hybridizing, nucleoside, cepak, oscillin, opt3, cdc42ce, lh, overlapping, representing, aqp, bip, smt3, middle, onchocerca, clones, segregator, pyrimidine, designed, nucleosome

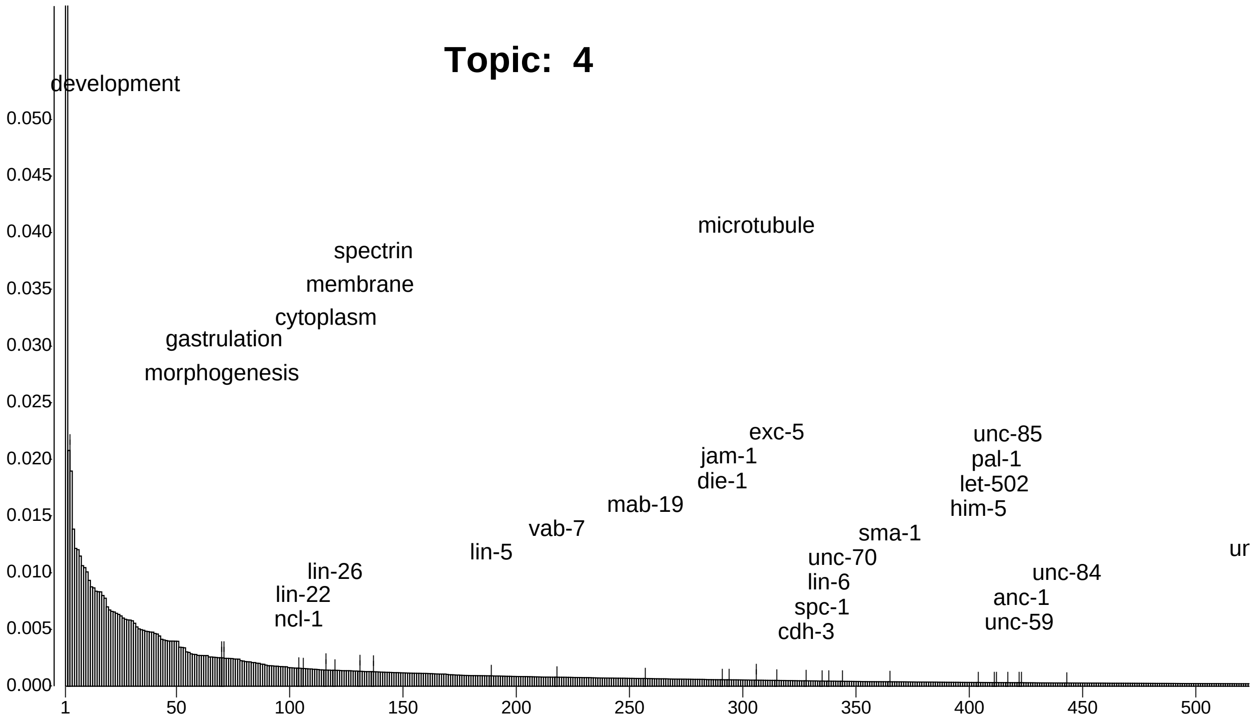

GO-based labels: *MF:* ; *CC:* mitochondrial outer membrane, cytoplasmic chromosome, mitochondrial membrane, peroxisomal membrane; *BP:* ethylene mediated signaling pathway, osmosensory signaling pathway via two-component system, abscission, aging, two-component signal transduction system 'phosphorelay'
CGC-based labels: hypodermal, seam, excretory, epidermi, apical, adheren, epithelia, junctions, morphogenetic, differentiate, autonomously, epithelium, hypodermis, primordium, syncytium, integrity, sulston, serial, lin5, canal, ectodermal, hyp7, speculate, dev, vab7, horvitz, autonomou, boundary, duct, surrounding, acquire

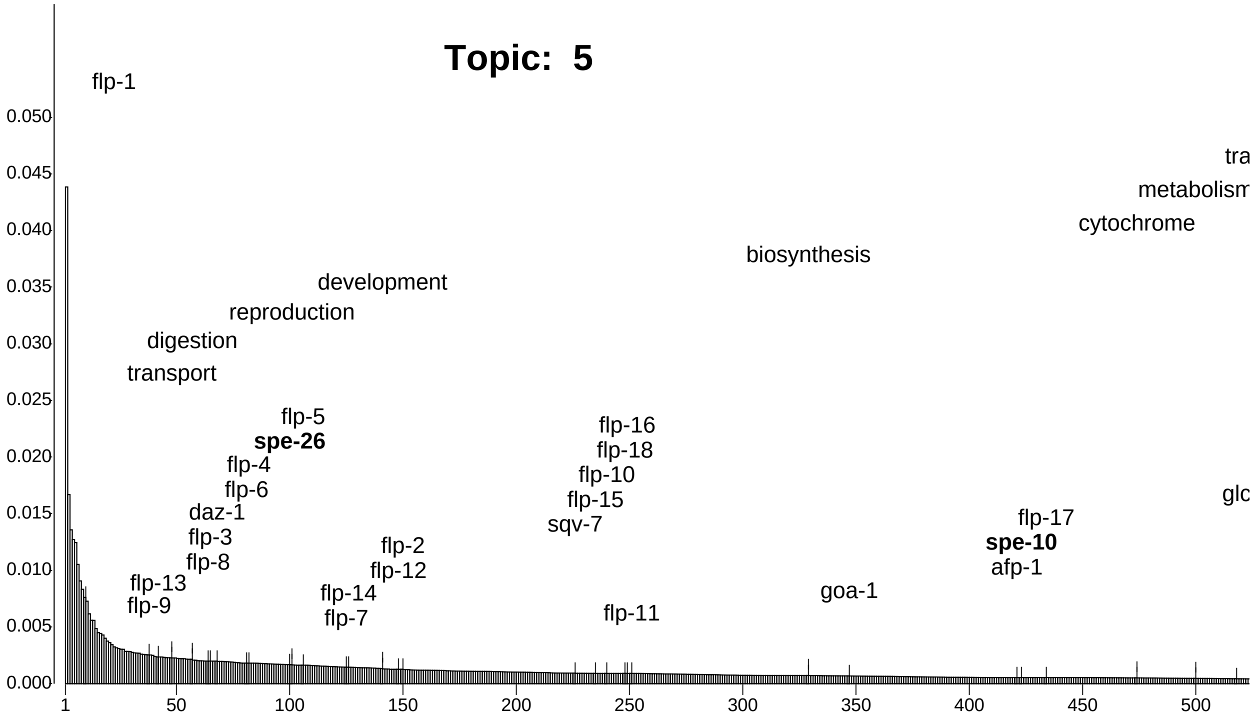

GO-based labels: *MF:* ; *CC:* ; *BP:* regulation of cholesterol absorption, cholesterol absorption, abscission, aging **CGC-based labels:** farp, flp1, twodimensional, heparan, spot, glycosaminoglycan, fmrfamiderelated, fmrfamidelike, phe, spectrometry, immunoreactivity, flp9, flp13, chondroitin, arg, nh2, digestion, met, flp3, flp8, fb, strip, daz1, resolution, fmrfamide, flp4, flp6, microvilli, udp, glycosphingolipid, af2, flp5, bodymembranou, mated, flp14, flp7, af1, hedgehog, immunoreactive, antiserum, flp12, af8, desorption, ksaymrfamide, flp2, gerp95, achieved, conformational, silver, fmrf, daz, gasphase, timeofflight, edman, hplc, subjected, peptides, ala, moiety, successful, accurate, sqv7, disaccharide, amide, flp10, flp11, flp15, flp16, flp18, kheylrfamide, dehydroergosterol, pf1, pf3, mh, svpgvlrfamide, spermatogonial, carbonylated, sdpnflrfamide, spectrometer, phosphorylcholine, matrixassisted

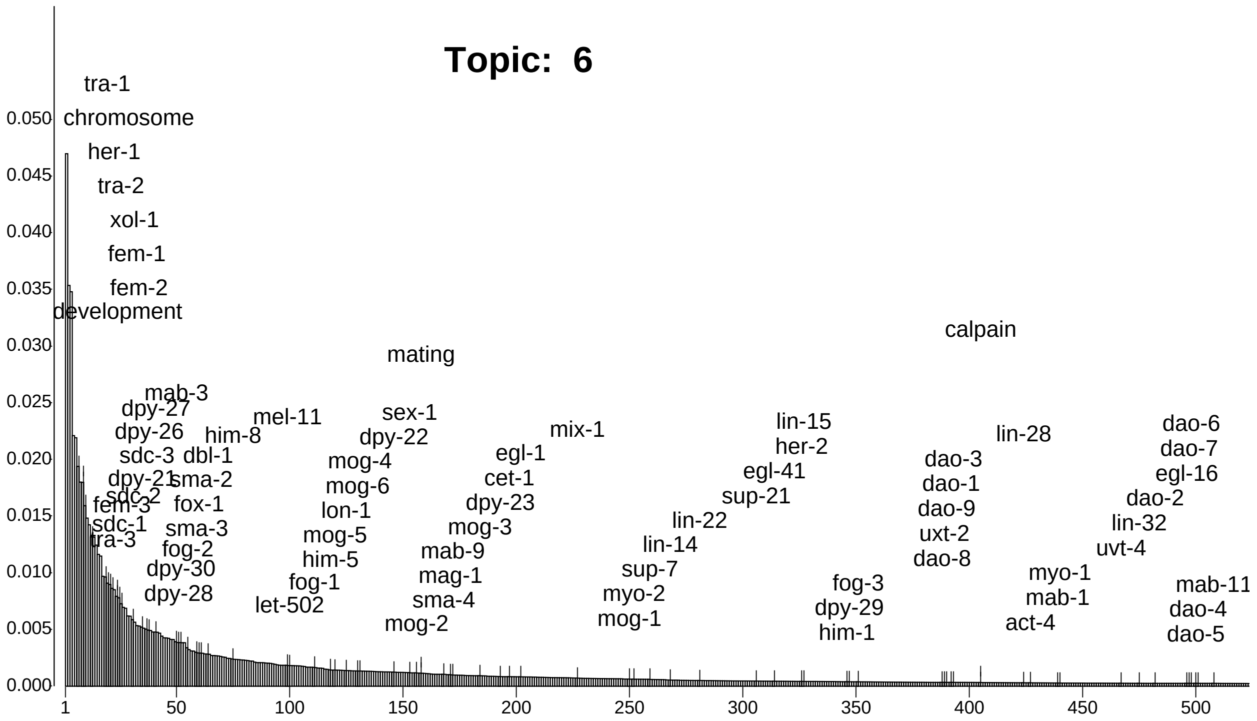

GO-based labels: *MF:* exodeoxyribonuclease; *CC:* ; *BP:* ethylene mediated signaling pathway, osmosensory signaling pathway via two-component system, two-component signal transduction system 'phosphorelay', negative regulation of transcription by pheromones, positive regulation of transcription by pheromones, regulation of transcription from pol i promoter mitotic, ethylene mediated signaling pathway 'induced systemic resistance', ethylene mediated signaling pathway 'jasmonic acid/ethylene dependent systemic resistance', mapkkk cascade 'osmolarity sensing', negative regulation of transcription from pol i promoter mitotic, positive regulation of transcription from pol i promoter mitotic, thermoregulation, fat body metabolism 'sensu insecta', fever, homoiothermy, negative regulation of transcription from pol ii promoter by pheromones, positive regulation of transcription from pol ii promoter by pheromones, abscission, aging, development of primary sexual characteristics, development of secondary sexual characteristics **CGC-based labels:** sex, tra1, dosage, her1, xx, compensation, sexual, xo, xol1, tra3, sdc1, fem, males, sdc2, dpy21, sdc3, dpy26, dpy27, sexe, mab3, dpy28, dpy30, hermaphrodites, fox1, sexspecific, dbl1, feminizing, fog, sexdetermination, copulatory, masculinization, sma, mel11, sexually, sdc, feminization, dimorphism, malespecific, hierarchy, masculinizing, mog5, transform, mog6, mog4, lf, dpy22, sex1, pp2c, mog2, sexes, mag1, mel, global, numerator, mog3, mndp8, stdp2, mndp10, dpy23, equalize, dsx, cet1, cpeb, coordinately, dimorphic, diploid, feminize, bmp, triploid, sexlinked, compensate, mix1, mndp9, mndp25, females, mndp27, bursa

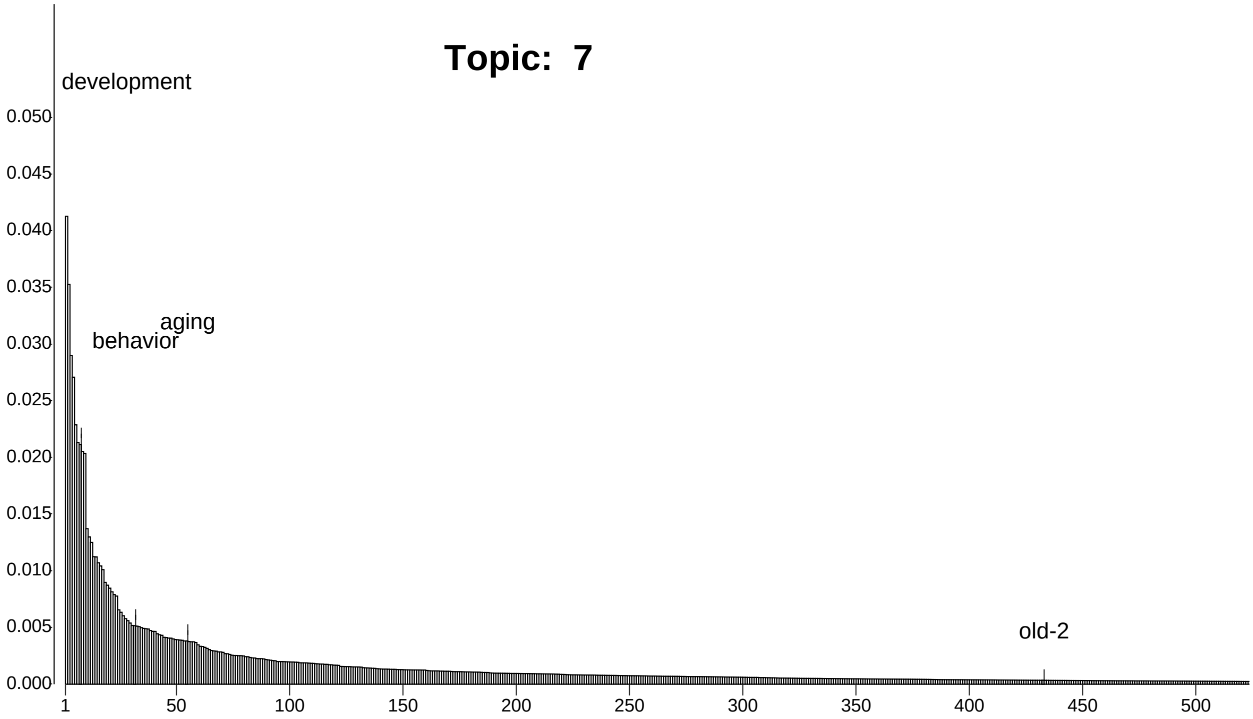

GO-based labels: *MF:* ; *CC:* ; *BP:* adult feeding behavior, abscission, aging, adult behavior, determination of adult life span **CGC-based labels:** review, detailed, progress, provided, past, offer, focu, chapter, advance, area, systems, brenner, reviewed, example, physiology, opportunity, description, summarize, excellent, phenomena, dissection, genetics, numerou, mechanisms, attention, address, contribution, way, emerged, difficult, briefly, interested, focused, challenge, volume, simplicity, investigator, sparc, sydney, kingdom, remarkable, invertebrates, highlight, elucidation, ideal

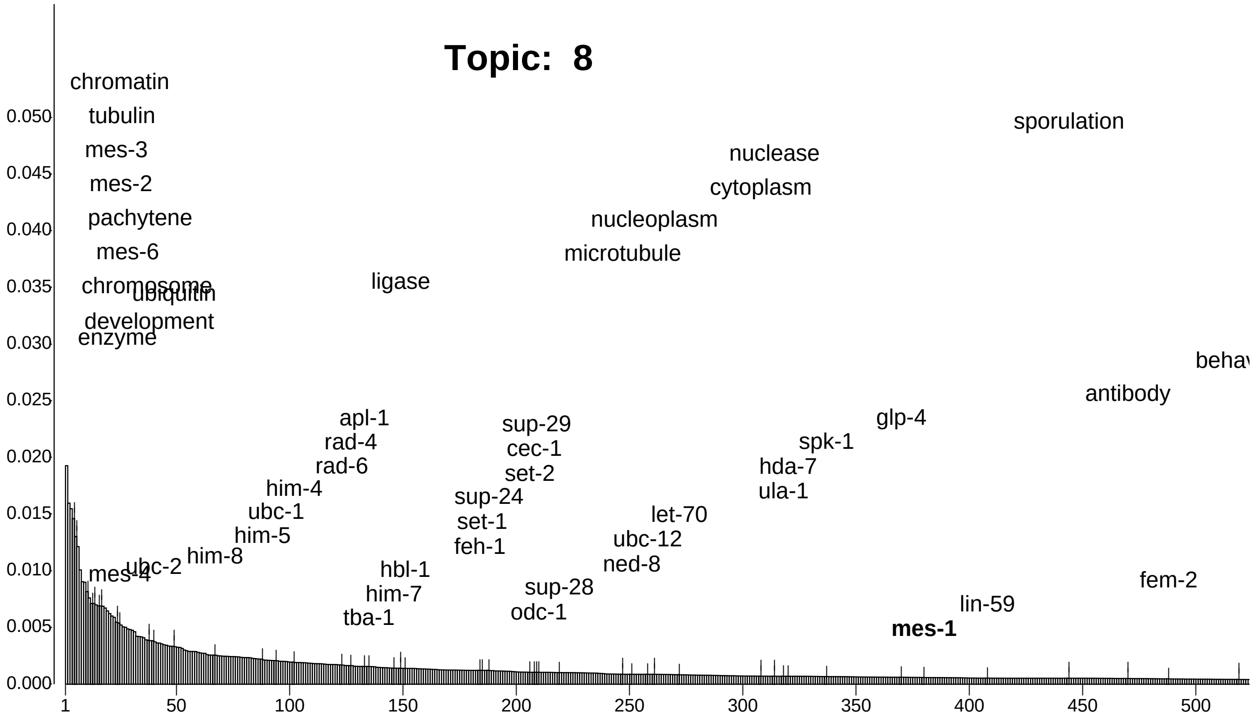

GO-based labels: *MF:* ; *CC:* axonemal microtubule; *BP:* abscission, aging, polyubiquitination, spermidine biosynthesis **CGC-based labels:** mes, sc, mes3, mes2, mes6, synaptonemal, mes4, sr, ubc, ubc2, polycomb, topoisomerase, tdna, emap, isotype, karyotype, ferm, scs, trp, drr, ubiquitinlike, knob, ubc1, him4, ned, ubiquitinconjugating, mttrna, colchicine, ubc4, univalent, anticodon, apl1, tba1, tertiary, him7, ligase, hbl1, trnapro, mbz, polyamine, condensation, betaamyloid, susceptible, odc, cca, feh1, lon, tu, set1, conjugating, ubc5, sup24, notion, ornithine, cesf2, odc1, cec1, set2, conjugation, ceatl1, sup29, dimethyl, proteasome, wt, mnt6, tyr, sup28, mebendazole, modifying, senescent, incidence, plaque, e2, ned8

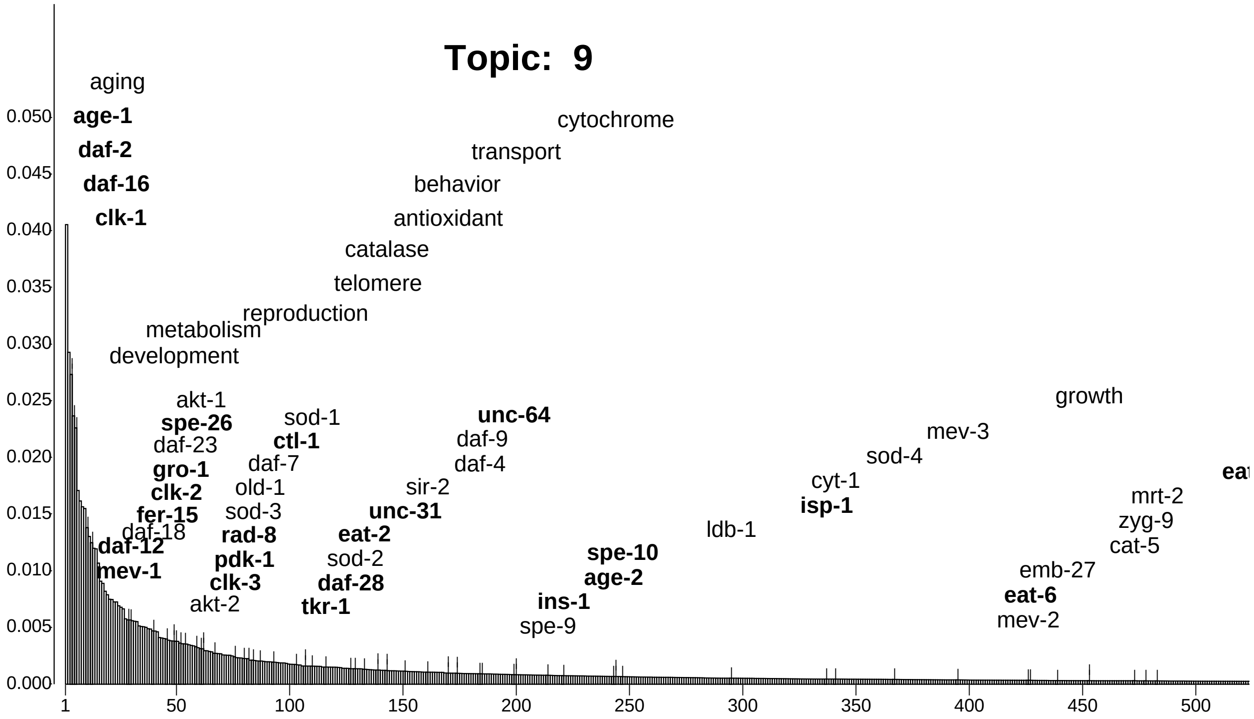

GO-based labels: *MF:* ; *CC:* mitochondrial derivative, kinetoplast, mitochondrial degradosome, microsome, telomerase holoenzyme complex; *BP:* adult feeding behavior, determination of adult life span, abscission, aging, drug metabolism, adult behavior **CGC-based labels:** age1, longevity, lifespan, clk1, ageing, insulin, mev1, insulinlike, longlived, daf18, superoxide, fer15, clk2, gro1, daf23, clk, akt1, dismutase, akt2, extended, consumption, clk3, pdk1, rad8, sod3, pten, old1, diapause, ctl1, aged, thermotolerance, caloric, igf, tkr1, gerontogene, daf28, agespecific, coq7, sod2, expectancy, ubiquinone, receptorlike, dramatically, carbonyl, sod, sir2, hx546, antioxidant, capacity, tension, paraquat, daf9, itt, lengthen, cln3, agedependent, pi3k, chronological, fashion, nondauer, postreproductive, ins1, replica, lived, slow, slowing, singlegene, age2, coenzyme, spe10

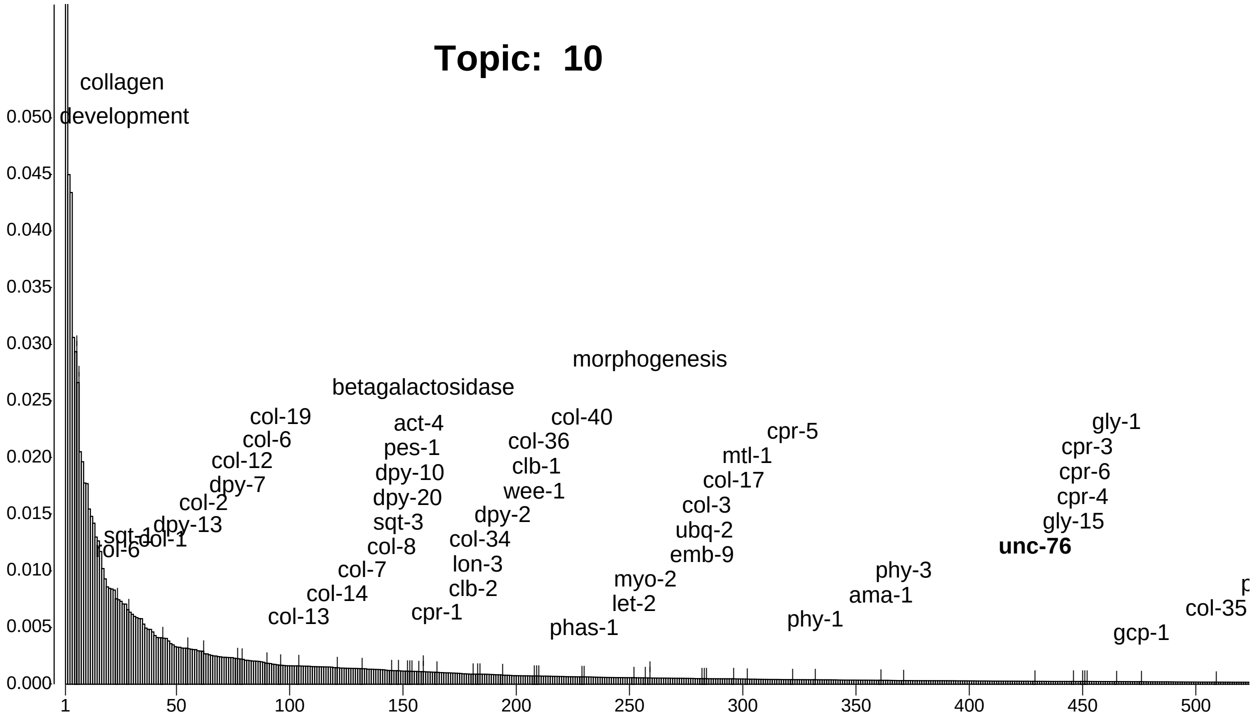

GO-based labels: *MF:* ; *CC:* ; *BP:* abscission, aging, thermoregulation, fat body metabolism 'sensu insecta' **CGC-based labels:** larval, collagen, l1, stages, molt, col1, l4, l2, col2, col12, col6, adults, col13, differentially, alpha2, col14, pkc1b, collagens, col7, correlate, col8, collagenou, shift, cpr1, adulthood, exoskeleton, moult, phy, clb2, lon3, col34, postdauer, survive, start, abundantly, wee1, clb1, col36, patterns, steadystate, twofold, l2d, phas1, col40, sqt, col, microinjection, hsp90, morphologically

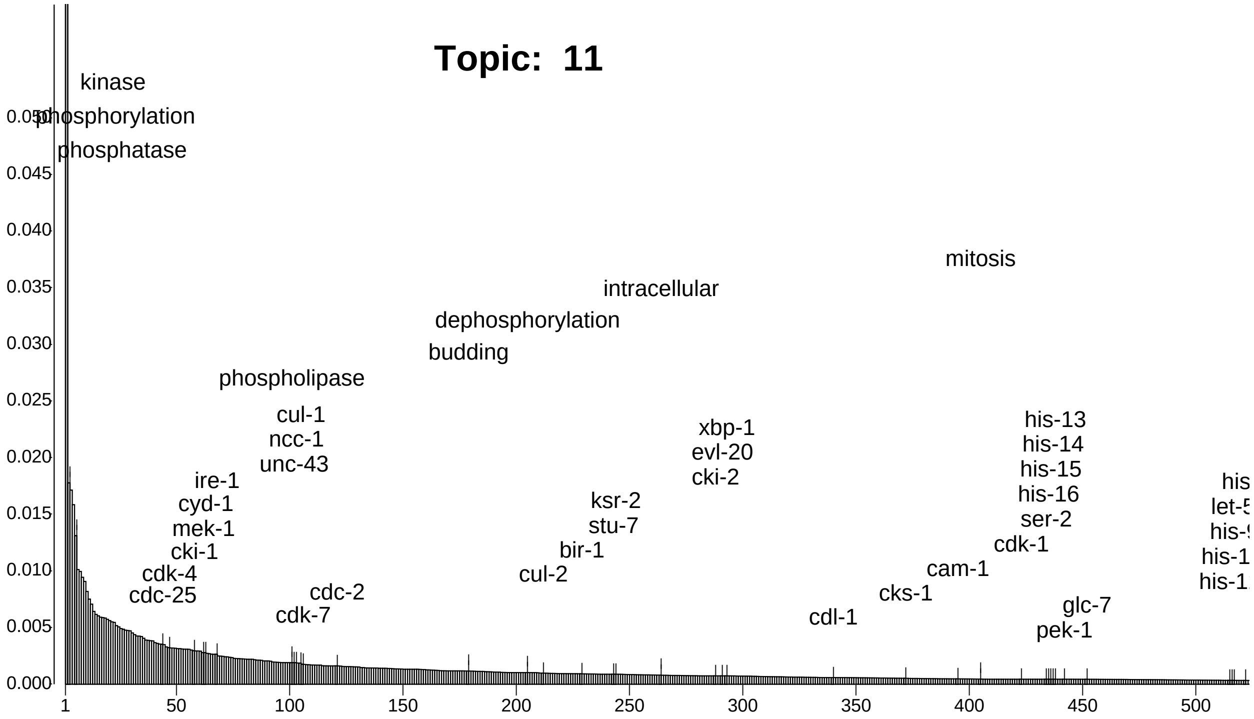

GO-based labels: *MF:* cytosolic phospholipase a2, secreted phospholipase a2, phospholipase d, phospholipase a1, phospholipase a2; *CC:* ; *BP:* protein amino acid dephosphorylation **CGC-based labels:** phosphorylation, akt, progression, cam, threonine, jnk, phosphorylated, ksr, cdc25, cdk4, phc, cki1, cdk, camkii, mek1, cyd1, hcf, ire1, g1, acclimatization, kinases, phosphorylate, pkb, calmodulindependent, thr, kringle, bir, ctd, icp, birp, guanylate, phospholipase, ncc1, cul1, cdk7, cak, hbp, cyclindependent, cdc2, p38, hfem, cehcf, plm, incenp, c2, fn2, upr, cki, pld, plc1, dynamical, rhobinding, adaptive, pvn, ck2, polo, perk, cetfiie, piak, tumour, ror, unfolded, abolished, hand, expanded, csk1, polyq, cip, cak1, plk, plc2, xbp, kip, dephosphorylation, cho, lumbar, vp16, cul2, cdc, elimination, autophosphorylation, bir1, iap, ksr2, hairpinbinding, stu7, ser5, gin, mpf, nld, replace, plc

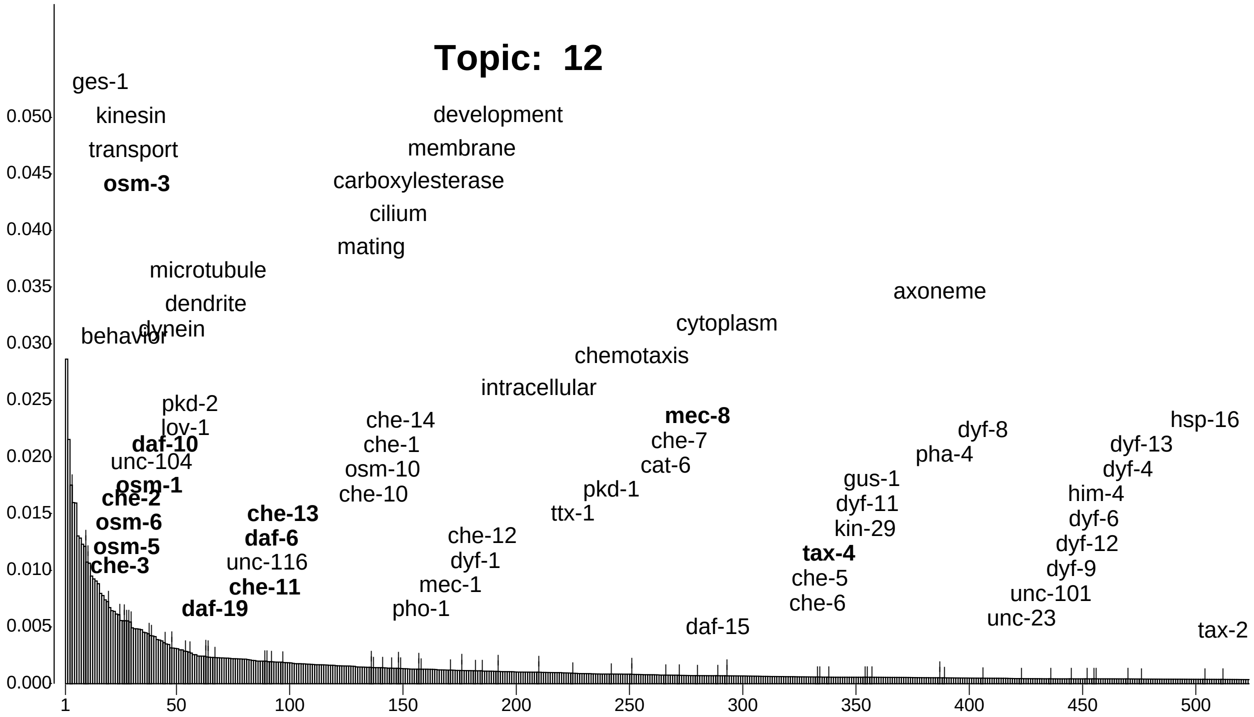

GO-based labels: *MF:* ; *CC:* axonemal microtubule, mitochondrial outer membrane, mitochondrial membrane, peroxisomal membrane, apical part of cell, basal part of cell; *BP:* immune cell chemotaxis, adult feeding behavior, ethylene mediated signaling pathway, osmosensory signaling pathway via two-component system, adult behavior, two-component signal transduction system 'phosphorelay', abscission, aging, neutrophil chemotaxis **CGC-based labels:** ges1, cilia, kinesin, esterase, amphid, osm3, pha, osm5, che2, ash, osm1, retrograde, ending, avoidance, sensilla, amphidial, ciliated, lov1, gutspecific, pkd2, phasmid, ift, gesl, dendrite, daf19, cargo, che, repellent, anterograde, ir, che11, unc116, che13, avoid, intraflagellar, wgatar, che10, cephalic, osm10, crystallin, che1, moving, cilium, che14, digestive, isoelectric, carboxylesterase, pho1, chaperonelike, cog, focusing, ciliary, rectum, tagged, deleted, dyf1, con, dendritic, che12, ase, perception, sorting, fluorescein, repellents, raft, polycystic, lip, dendrites, fitc, pkd1, adl, chemoreception, deirid, flagella, dimeric, chemotaxis

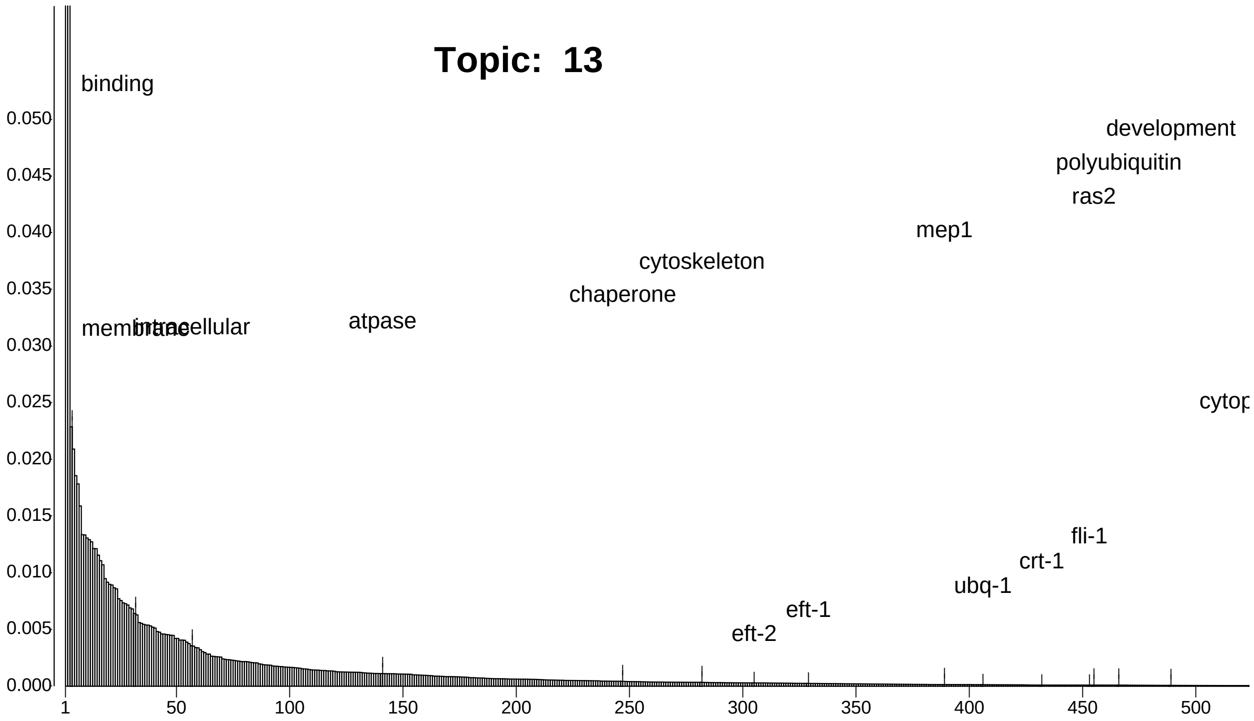

GO-based labels: *MF:* amino acid binding, calcium ion binding; *CC:* mitochondrial outer membrane, mitochondrial membrane, peroxisomal membrane, apical part of cell, basal part of cell; *BP:* **CGC-based labels:** motif, terminu, domains, hydrophobic, carboxyterminal, ef, aminoterminal, truncated, carboxyl, aminoacid, shared, pdz, acidic, proline, monomer, zipper, motifs, coiledcoil, atypical, terminus, forming, consisting, conventional, ao13, tm, interestingly, enriched, samdc, linker, presumed

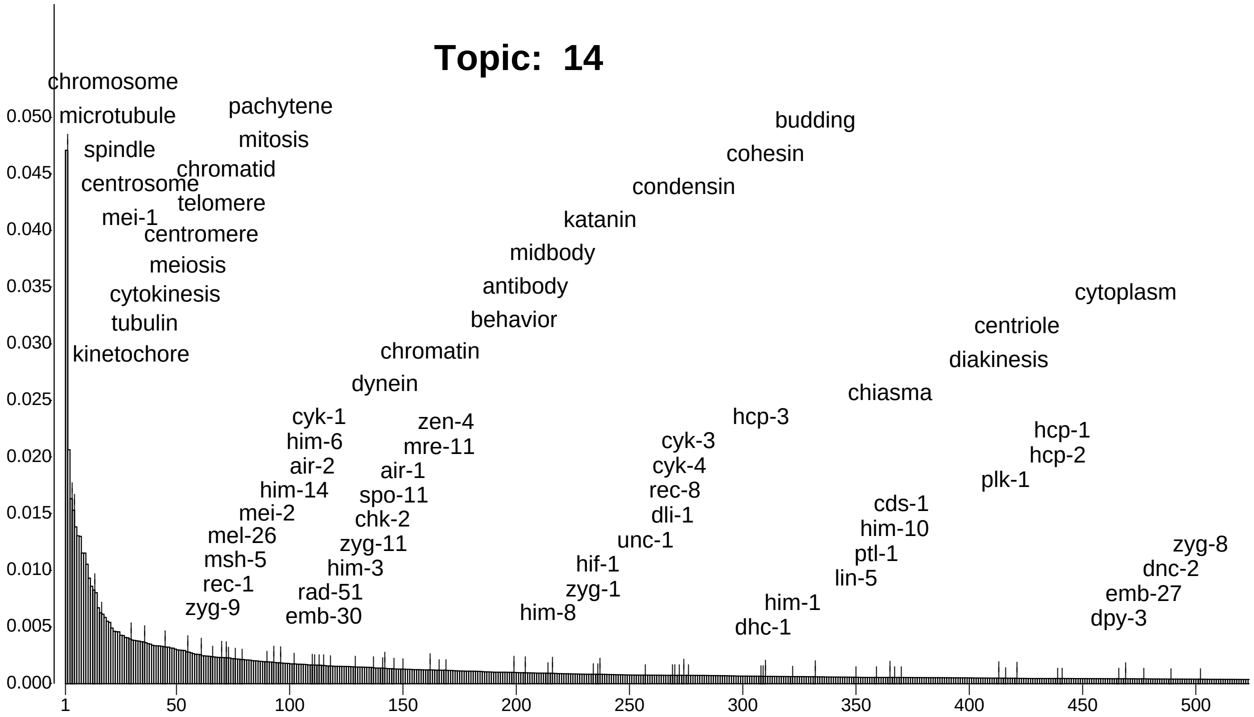

GO-based labels: *MF:* ; *CC:* mitochondrial derivative, axonemal microtubule, centriole, telomerase holoenzyme complex, dynein; *BP:* adult feeding behavior, female meiosis i spindle assembly, female meiosis ii spindle assembly, meiotic spindle assembly, adult behavior **CGC-based labels:** air, mei1, cytokinesi, prophase, metaphase, kinetochore, furrow, anaphase, cytokinesis, centromere, aurora, crossover, chromatid, rec1, msh5, mel26, microtubules, mei2, zen, mitosis, him14, hcp, cyk, cohesion, air2, him6, cyk1, rad51, centrosomal, szt1, aster, him3, zyg, mei, holocentric, zyg11, midzone, chk2, mnt12, spo11, air1, synapsi, interphase, segregate, rec, mre11, breakage, zen4, mix, tau, pronuclear, chiasmata, hin1, depleted, crosslink, homozygote, promoting, poles, recombine, inversion, pronuclei, hif1, katanin, gammatubulin, dispensable, microtubuleassociated

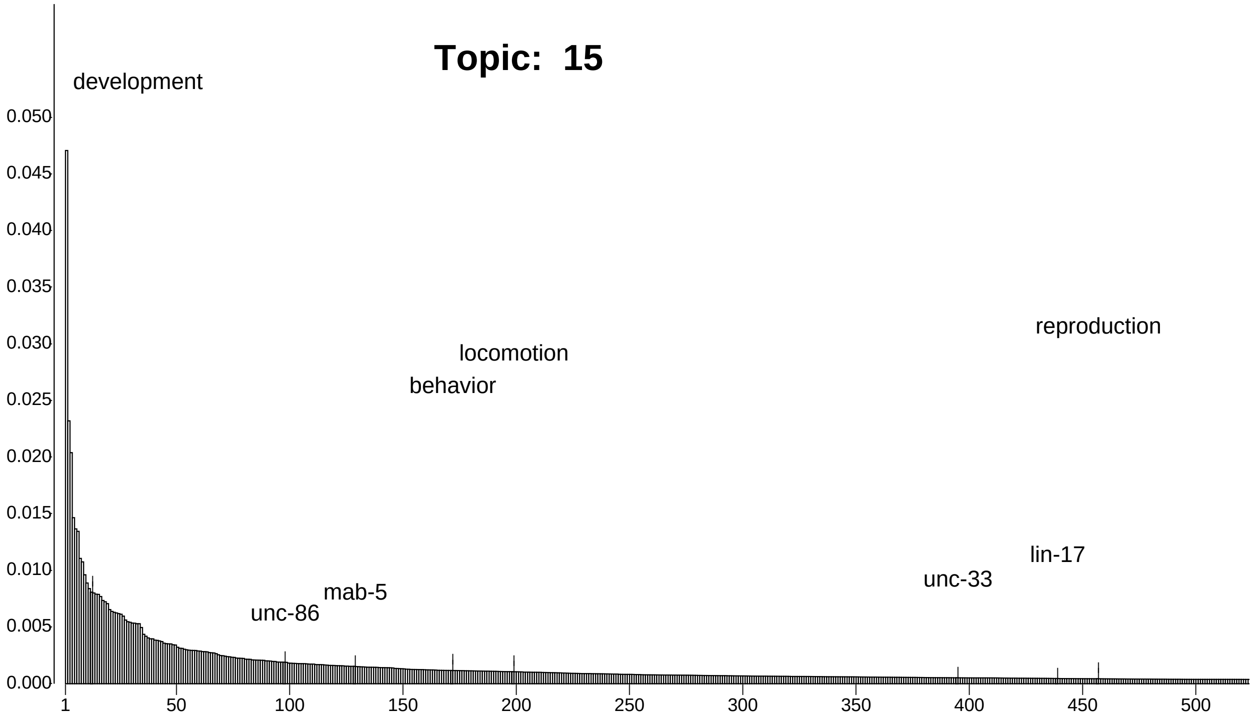

GO-based labels: *MF:* ; *CC:* ; *BP:* adult feeding behavior, abscission, aging, adult behavior, regulation of locomotion **CGC-based labels:** ab, dom, abp, founder, aba, sediment, reproducible, p2, aquatic, microbeam, invariance, symmetrical, anatomical, tracking, influenced, cellautonomou, typical, chosen, assessing, interpretation, nongonadal, born, p1, refractory, considered, earthworm, cdcl2, reproducibility, quality, bilaterally, dictated, summary, extra, clay, nanu, regulative, morgan, determinate, observing, replaced, traced, strictly, petri

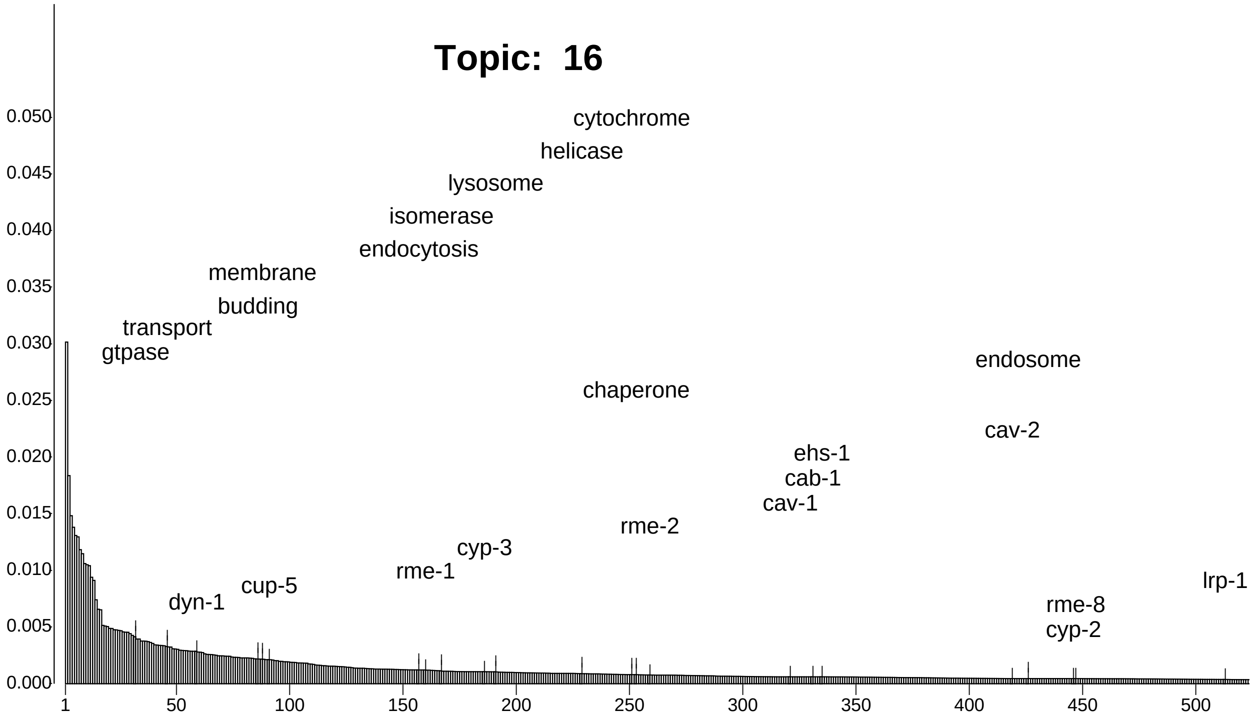

GO-based labels: *MF:* ; *CC:* mitochondrial outer membrane, mitochondrial membrane, peroxisomal membrane; *BP:* **CGC-based labels:** alignment, pombe, endocytosi, arabidopsi, recycling, rme, endocytic, orthologou, thaliana, trafficking, globin, schizosaccharomyce, gef, dyn1, dynamin, homo, rab, cyp, bag, caveolin, www, prokaryotic, tree, pfam, cup5, man, mmp, ra, gdp, diversification, http, assignment, sapien, phylogenetically, matche, cav, chemoreceptor, hidden, searche, frequent, searching, survey, gain, tpi, endocytosis, sponge, rme1, fission, unicellular, prokaryote, hmm, tm4sf, auxilin, rap1a, fatp, glo, lcfa, edu, cyp3, uptake, lysosome, hsc70, classification, ytubulin, pleckstrin, distant, rhoa, adaptin, cerhogdi, fkbp, vgs, p450, lmp, cerhoa, eh, receptormediated, eps15, ubiquitou, orthology, orthologs, markov, proteincoding, analyze, criteria

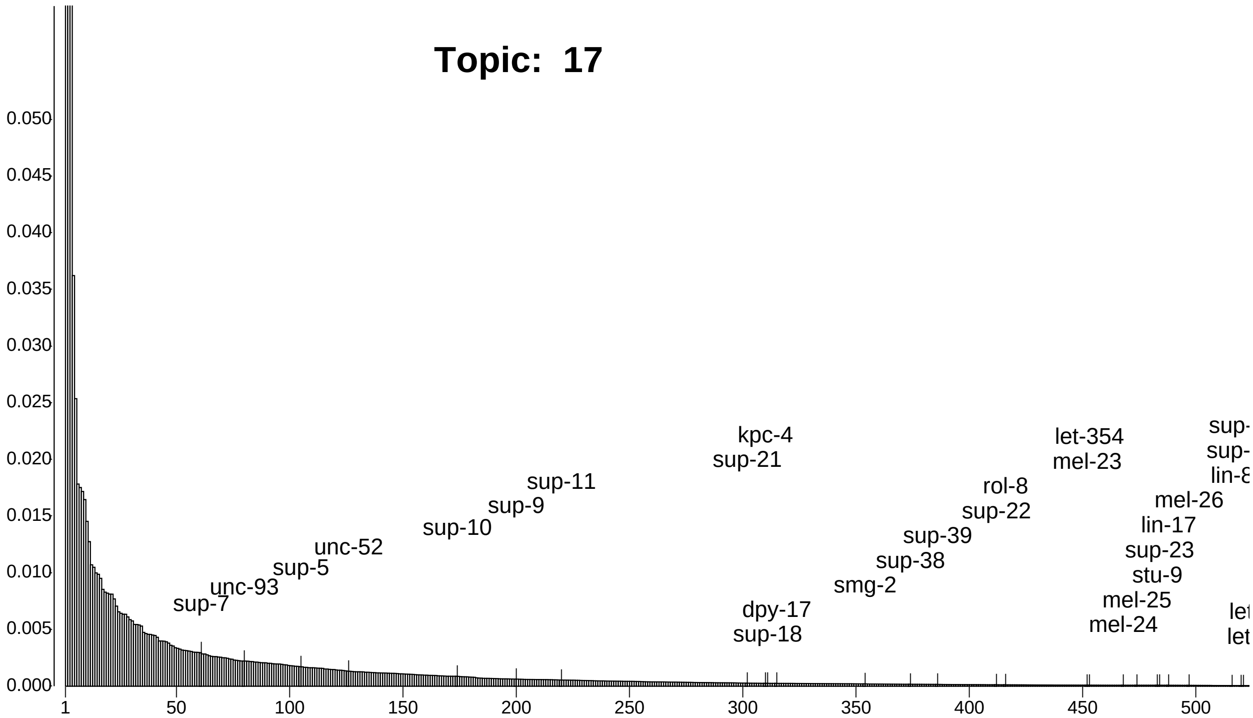

GO-based labels: *MF:* ; *CC:* ; *BP:* **CGC-based labels:** dominant, suppress, mutations, severe, sup7, unc93, alter, dumpy, phenotypes, sup5, alleles, hypomorphic, extragenic, semidominant, penetrant, sup10, independently, restore, allelic, causing, remaining, allelespecific, disrupted, sup9, impaired, penetrance, conditional, mild, severity, products, sup11, pleiotropic, displayed, incompletely, incomplete, st5, nonessential

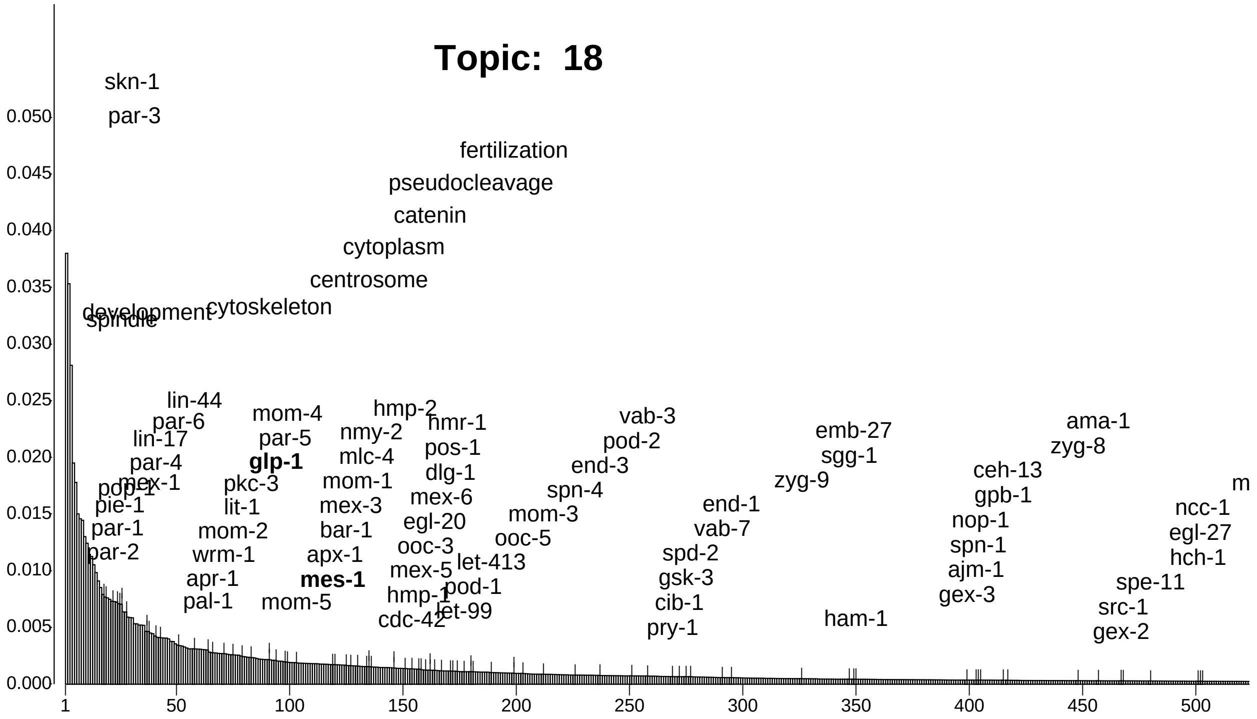

GO-based labels: *MF:* exodeoxyribonuclease, endodeoxyribonuclease, endoribonuclease, 5'-3' exonuclease, purine-specific oxidized base lesion dna n-glycosylase, pyrimidine-specific oxidized base lesion dna n-glycosylase, pyrimidine dimer dna n-glycosylase, flap endonuclease; *CC:* intermediate filament cytoskeleton, centriole, cytoplasmic chromosome; *BP:* abscission, aging, cell wall modification during abscission, determination of adult life span, adipocyte differentiation, endothelial cell differentiation, regulation of adipocyte differentiation, regulation of endothelial cell differentiation, regulation of locomotion, ethylene mediated signaling pathway, osmosensory signaling pathway via two-component system, locomotion, two-component signal transduction system 'phosphorelay', flocculation 'sensu saccharomyces' **CGC-based labels:** polarity, par, par3, par2, par1, pie1, pop1, establishment, par4, par6, pop, asymmetrically, lin44, apr1, axe, wrm1, mom2, blastomeres, lit1, unequal, mex, establish, par5, mom4, mom5, polarization, betacatenin, onecell, rotation, establishing, cortex, segregated, mom1, mlc4, nmy2, partitioning, hmp2, cdc42, hmp1, mex5, ooc3, tcf, catenin, mex6, wrm, hmg, generating, dlg1, hmr1, bar, let99, pal, ql, pseudocleavage, pod1, responding, let413, twocell, apc, hmp, the, ooc5, lineagespecific, lit, mom3, cleavages, orient, periphery, polarize, gex, spn4, asymmetrical, end3, of, ooc, ajm

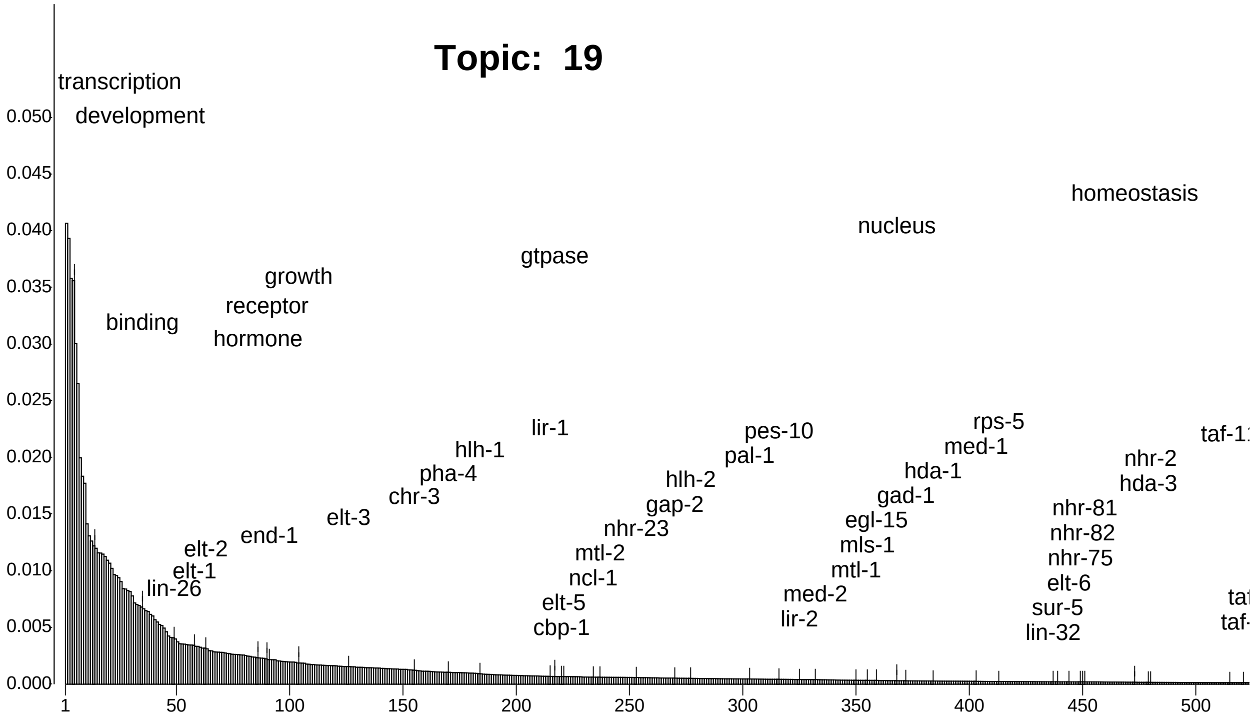

GO-based labels: *MF:* amino acid binding, calcium ion binding; *CC:* ; *BP:* ethylene mediated signaling pathway, osmosensory signaling pathway via two-component system, two-component signal transduction system 'phosphorelay', negative regulation of transcription by pheromones, positive regulation of transcription by pheromones, regulation of transcription from pol i promoter mitotic, ethylene mediated signaling pathway 'induced systemic resistance', ethylene mediated signaling pathway 'jasmonic acid/ethylene dependent systemic resistance', mapkkk cascade 'osmolarity sensing', negative regulation of transcription from pol i promoter mitotic, positive regulation of transcription from pol i promoter mitotic, negative regulation of transcription from pol ii promoter by pheromones, positive regulation of transcription from pol ii promoter by pheromones, negative regulation of transcription mitotic, regulation of transcription by pheromones, regulation of transcription mitotic, positive regulation of transcription mitotic, abscission, aging, negative regulation of transcription from pol ii promoter mitotic, negative regulation of transcription from pol iii promoter mitotic, positive regulation of transcription from pol ii promoter mitotic, positive regulation of transcription from pol iii promoter mitotic, activation of mapk 'osmolarity sensing', activation of mapkk 'osmolarity sensing', activation of mapkkk 'osmolarity sensing' **CGC-based labels:** gata, finger, elt1, elt2, factors, elt, activator, end1, repressor, cbp, elt3, hnf, mesoderm, forkhead, chr3, fork, zincfinger, p300, driven, deacetylase, heterodimer, transcriptionally, reductionoffunction, perform, cbp1, elt5, integrate, dn, taf, molecularly, ectoderm, inappropriate

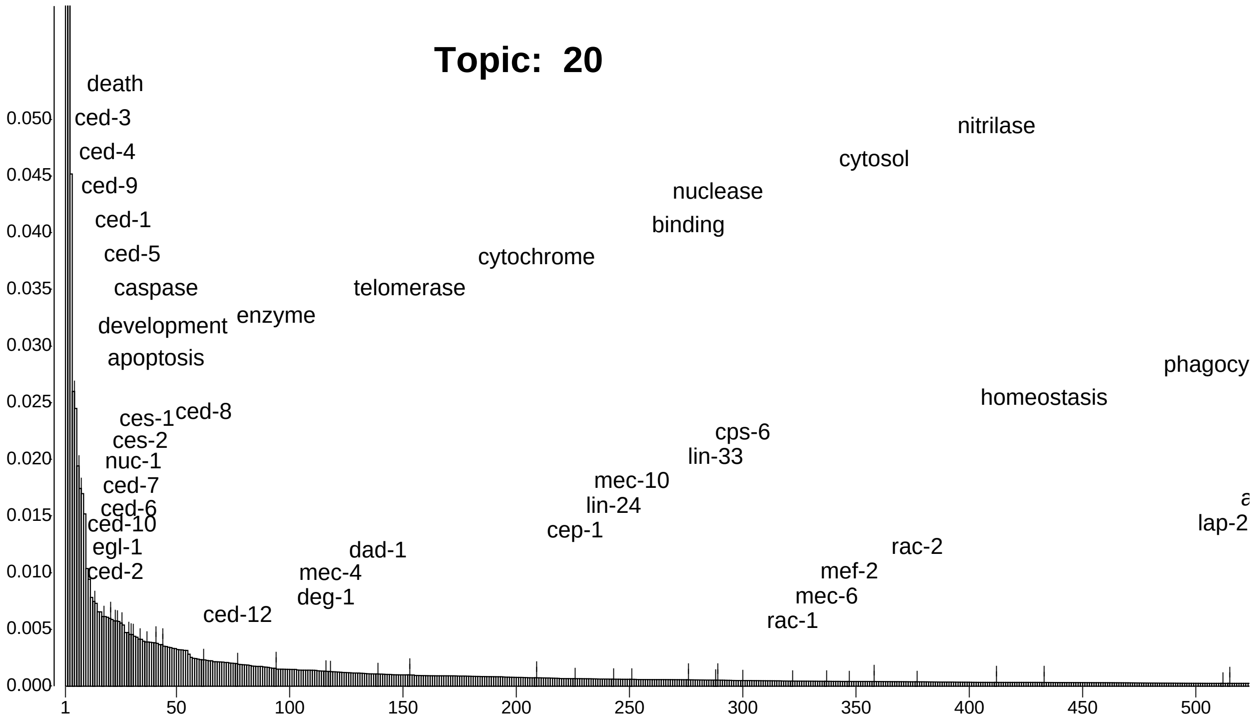

GO-based labels: *MF:* ; *CC:* ; *BP:* cell death, abscission, aging **CGC-based labels:** death, ced, ced3, programmed, ced4, ced9, apoptosi, ced1, apoptotic, bcl2, ced5, caspase, ced2, ced10, engulfment, ced6, ced7, nuc1, corpse, ces2, ces1, apoptosis, pcd, celldeath, ced8, ice, dying, phagocytosi, suicide, ced12, cebnip3, fhit, bh3, transglutaminase, engulfing, protect, p35, proapoptotic, dock180, cetwist, caspases, dad1, p53, e1b, csp, bcl, necrotic, telomerase, undergoing, interleukin1, killer, corpses, oligomerization, nit, converting, pathological, deaths, protective, flice, injury, necrosi, counterpart, cep1, apaf1, prodomain, lta, mac, damageinduced, lin24, homeostasi, tunel

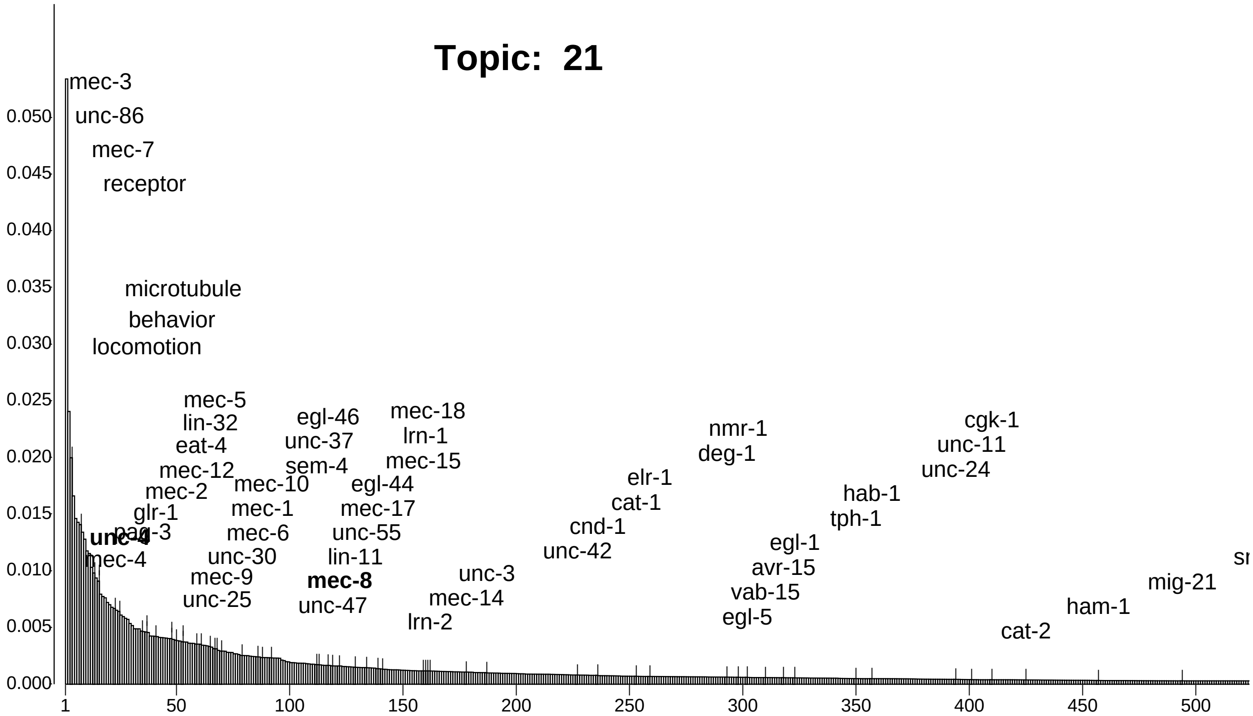

GO-based labels: *MF:* exodeoxyribonuclease; *CC:* axonemal microtubule; *BP:* ethylene mediated signaling pathway, osmosensory signaling pathway via two-component system, two-component signal transduction system 'phosphorelay', negative regulation of transcription by pheromones, positive regulation of transcription by pheromones, regulation of transcription from pol i promoter mitotic, ethylene mediated signaling pathway 'induced systemic resistance', ethylene mediated signaling pathway 'jasmonic acid/ethylene dependent systemic resistance', mapkkk cascade 'osmolarity sensing', negative regulation of transcription from pol i promoter mitotic, positive regulation of transcription from pol i promoter mitotic, negative regulation of transcription from pol ii promoter by pheromones, positive regulation of transcription from pol ii promoter by pheromones, regulation of transcription by pheromones, regulation of transcription mitotic, negative regulation of transcription mitotic, positive regulation of transcription mitotic, adult feeding behavior, aspartate transport, glutamate transport, negative regulation of transcription from pol ii promoter mitotic, negative regulation of transcription from pol iii promoter mitotic, positive regulation of transcription from pol ii promoter mitotic, positive regulation of transcription from pol iii promoter mitotic, activation of mapk 'osmolarity sensing', activation of mapkk 'osmolarity sensing', activation of mapkkk 'osmolarity sensing', regulation of locomotion, adult behavior, thermoregulation, fat body metabolism 'sensu insecta', acidic amino acid transport, drug metabolism **CGC-based labels:** touch, mec3, mec7, mechanosensory, circuit, tap, pag3, glr1, mec2, gabaergic, mec12, eat4, lin32, mec5, mec9, connectivity, withdrawal, backward, dd, glr, protofilament, locomotory, mechanosensation, reflex, unc37, vd, egl46, vb, alm, unc55, circuitry, stimulu, mec17, mns, egl44, bdu, nonassociative, gentle, excitatory, mec15, mec18, lrn1, lrn2, nose, eg1, interstimulu, cgk, mec14, pvd, antagonistic, lineal, touchinsensitive, avd, alphatubulin, homeoprotein, electrical, pvm, mechanoreceptor, habituated, cnd1, dishabituation, chalfie, neurogenic

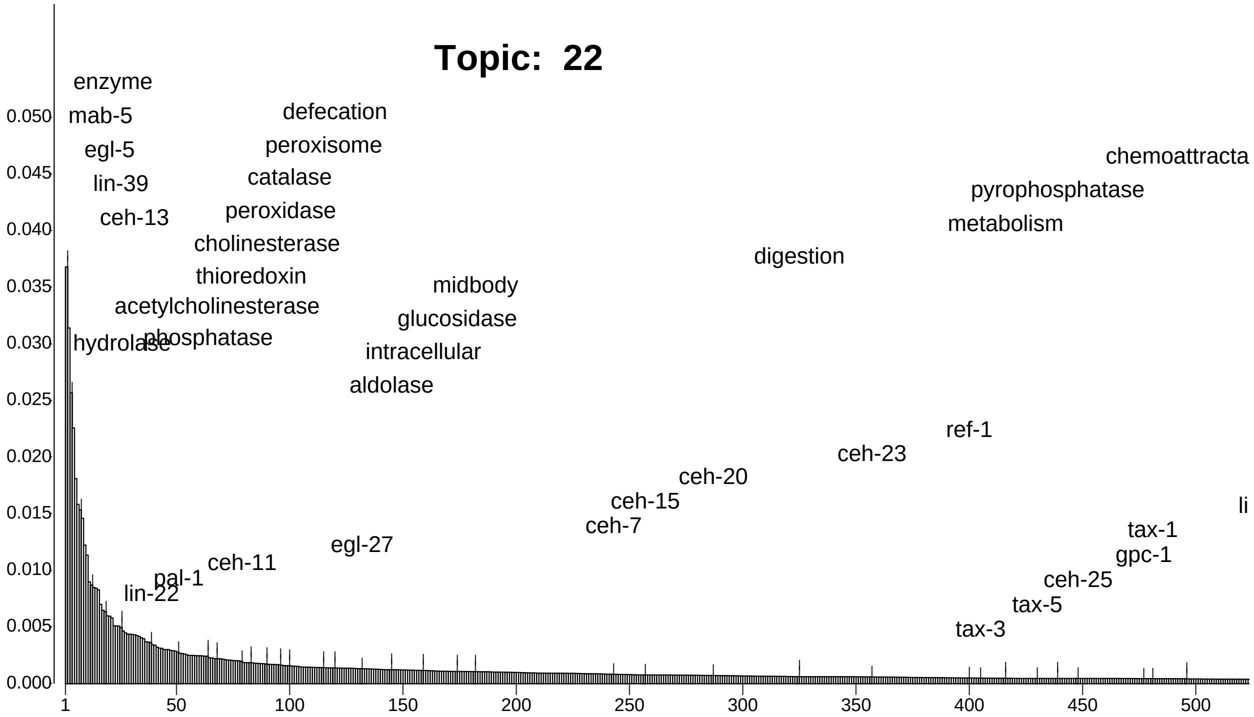

GO-based labels: *MF:* phospholipase d; *CC:* apical part of cell, basal part of cell; *BP:* ethylene mediated signaling pathway, osmosensory signaling pathway via two-component system, two-component signal transduction system 'phosphorelay' **CGC-based labels:** coa, dehydrogenase, nacl, ceh13, phosphate, hom, hydrolase, da, cathepsin, aminopeptidase, acetyltransferase, acetyl, wave, thiolase, chat, buffer, ceh11, nudix, thioredoxin, tryptophan, scpx, adh, antennapedia, enzymes, enzymatic, peroxisome, adp, substrates, minute, co2, regional, ivd, v6, aldolase, tr, km, capping, carrier, pts2, glucosidase, se, selenocysteine, adapt, tetraphosphate, isozyme, oxidation, activities, ce2, isovaleryl, vial, selenoprotein, ketoacyl, posteriorspecific, triphosphatase, countercurrent, ic50, angstrom, hairy, ceh7, diadenosine, decrement, nad, starved

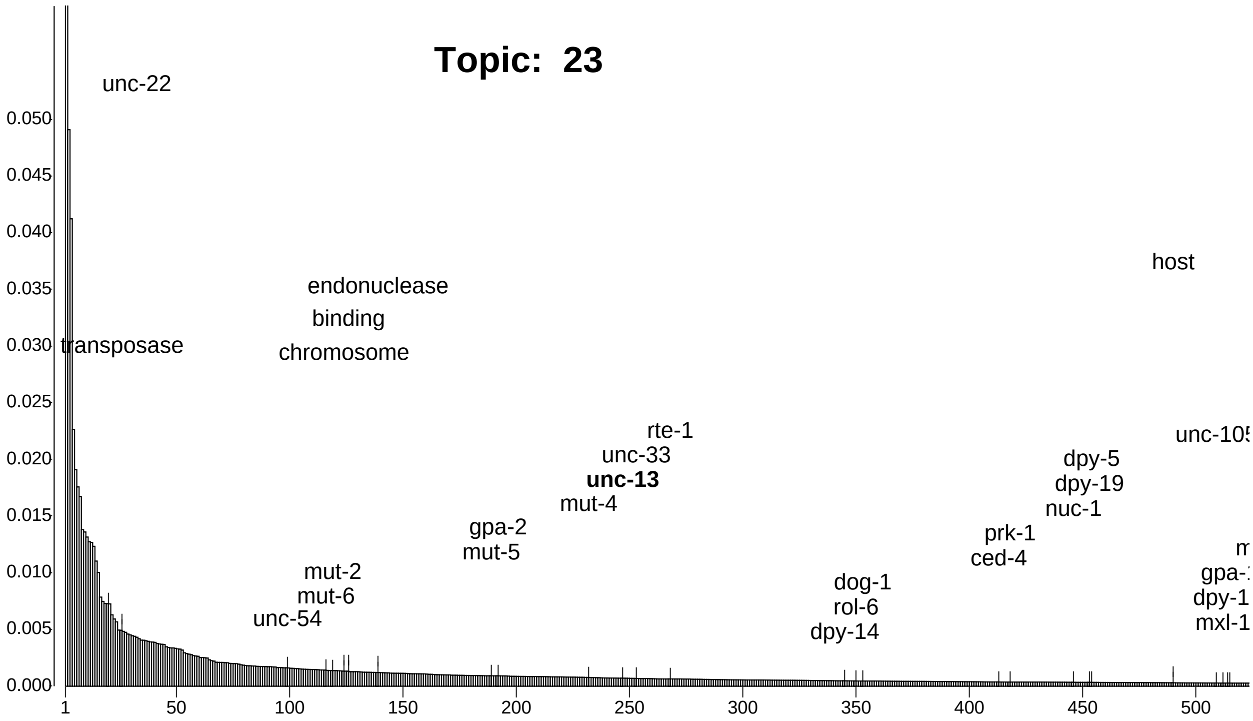

GO-based labels: *MF:* amino acid binding, calcium ion binding; *CC:* ; *BP:* hexose mediated signaling, carbohydrate mediated signaling **CGC-based labels:** tc1, transposon, transposable, excision, transposition, repetitive, tc3, inverted, transposase, elements, extrachromosomal, replication, mutator, tc4, ltr, mariner, tc5, footprint, tcb1, retrotransposon, circular, mut6, tagging, unstable, tca, tc2, groove, imprecise, flanked, tc1a, ta, tc6, jump, fish, interspersed, mut5, tc7, tr679, hot, hin, tcl, mobile, preferred, transpose, mut4, foreign, horizontal

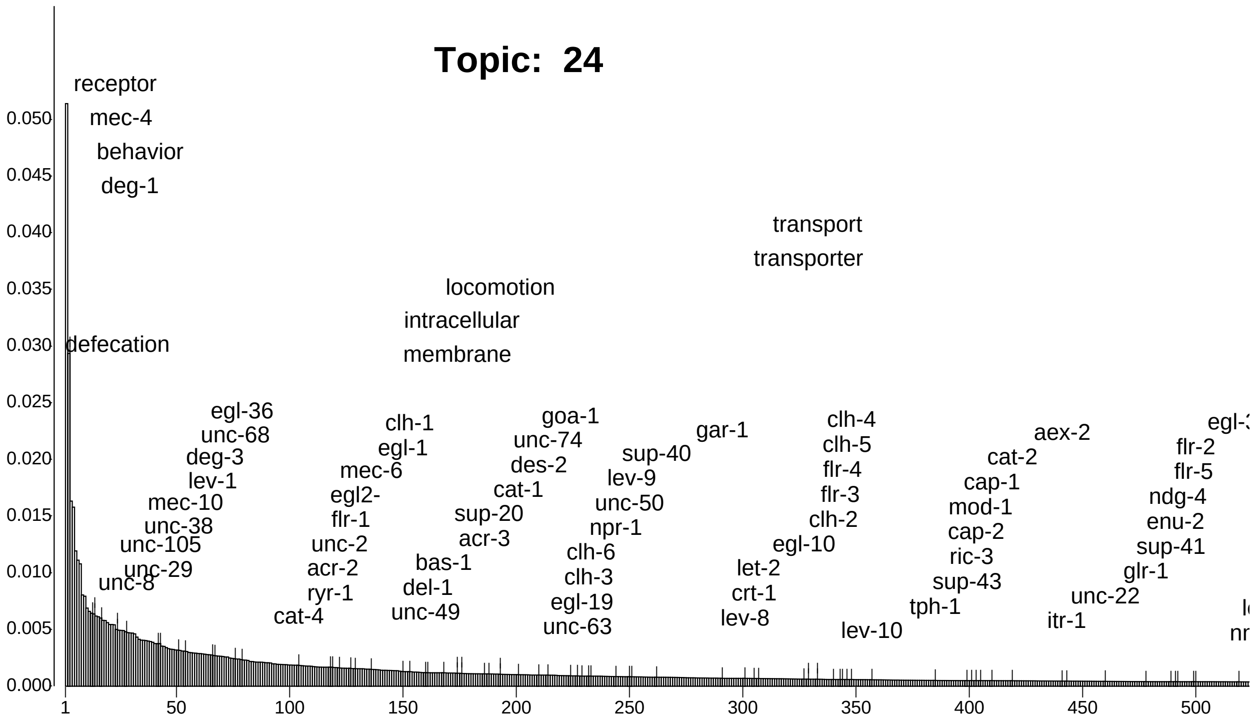

GO-based labels: *MF:* ; *CC:* mitochondrial outer membrane, mitochondrial derivative, mitochondrial membrane, peroxisomal membrane, apical part of cell, basal part of cell, microsome; *BP:* regulation of locomotion, drug transport, eye pigment precursor transport, 2-keto-3-deoxygluconate transport, adult feeding behavior, negative regulation of locomotion, positive regulation of locomotion, adult behavior, locomotion
CGC-based labels: serotonin, nicotinic, ht, degenerin, channels, nachr, unc8, levamisole, agonist, potassium, deg, enac, unc105, dopamine, rhythm, pharmacological, cation, deg3, gar, muscarinic, gated, antagonist, egl36, subunits, ryanodine, acr, cic, clh, cat4, modulate, clc, gating, ryr1, acr2, flr1, egl2, swelling, amiloridesensitive, nicotine, social, slo, clh1, electrophysiological, del1, nonalpha, conductance, alpha7, ce21, fluoxetine, bas1, mechanically, acr3, voltage, sup20, ionotropic, coexpressed, insensitive, proteinlinked, excitability, des2, imipramine, cp, pharmacologically, voltagedependent, circadian, modulation, permeability, nmda, mod, reuptake, clh3, clh6, machr, excitation, npr1, unc50, lev9

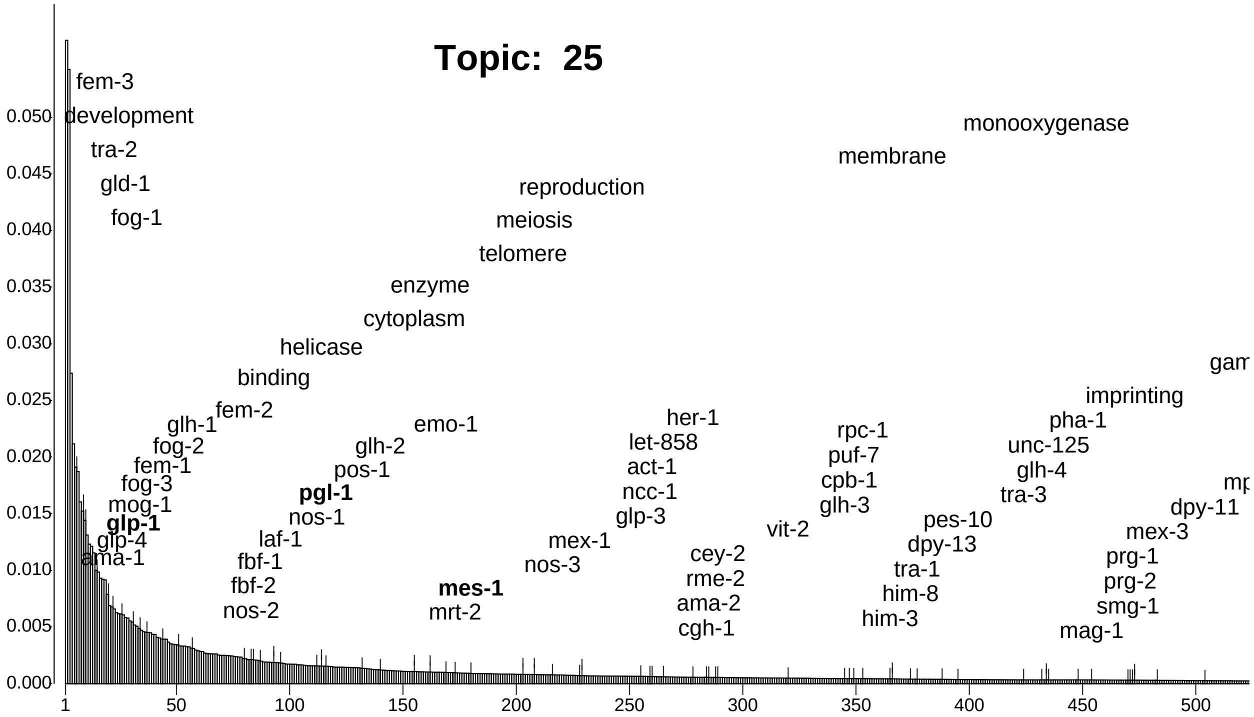

GO-based labels: *MF:* amino acid binding, calcium ion binding, exodeoxyribonuclease; *CC:* cytoplasmic chromosome, telomerase holoenzyme complex; *BP:* regulation of calcium in er, female meiosis i spindle assembly, female meiosis ii spindle assembly, abscission, aging, er to golgi transport, intra-golgi transport, meiotic spindle assembly, microgametogenesis **CGC-based labels:** germ, gld1, ama1, glp4, pie, gld, fog3, oogenesis, tge, glh1, germlinespecific, mog, uq, fbf, nos2, fbf2, fbf1, gli, laf1, gametogenesi, nos1, dmq, granules, pgl1, alphaamanitin, nano, mx, pgc, amanitin, glh2, posttranscriptionally, star, dre, pumilio, emo1, mrt2, rpo, m118, qki, nos, deah, drf, pgl, nos3, progenitor, drp, mdf4, tges, puf, spermoocyte, b12, homocysteine, achieve

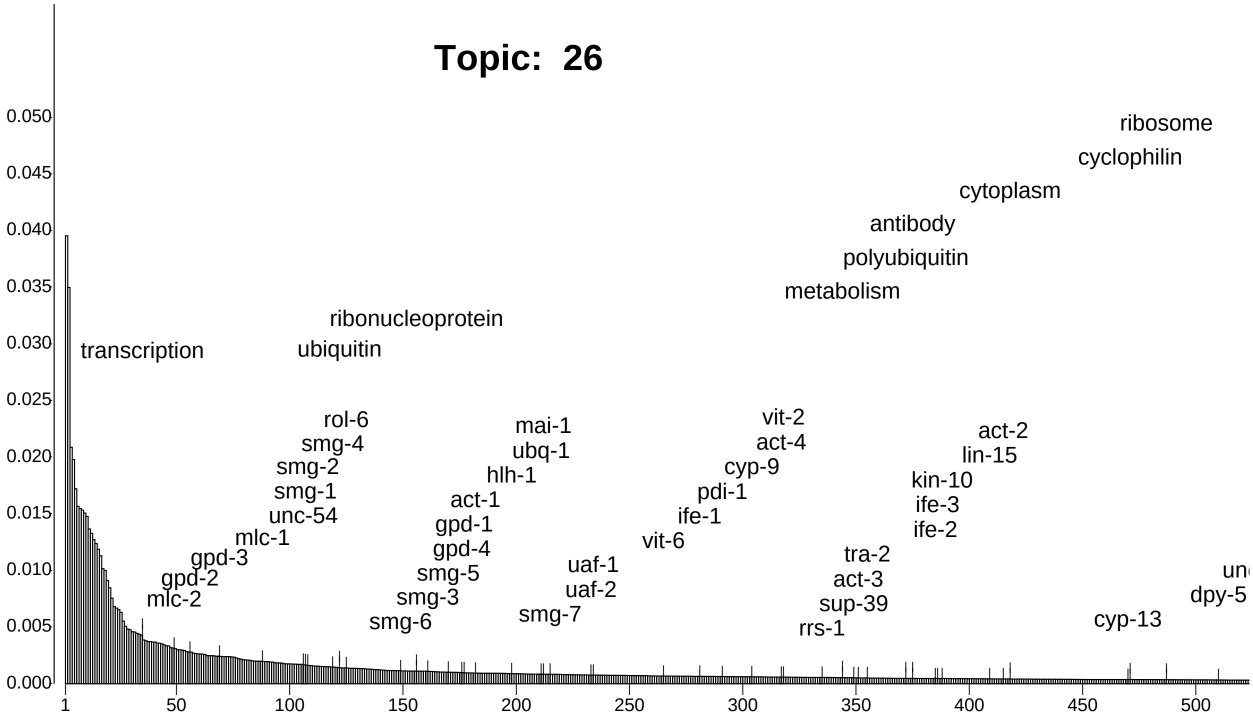

GO-based labels: *MF:* phosphatidylinositol 3-kinase class i; *CC:* ; *BP:* **CGC-based labels:** leader, spliced, splice, transsplicing, sl1, transspliced, sl2, premrna, sl, operon, smg, message, alternatively, polycistronic, tran, mlc2, acceptor, mrnas, gpd2, donor, cap, snrnp, ag, gpd3, transsplice, u2af, polyadenylation, ife, surveillance, mlc1, stop, snrna, eif4e, smg2, smg4, nmd, transcripts, smg6, hairpin, operons, ubia, trimethylguanosine, uracil, smg3, premature, decay, remove, smg5, tbp, gpd4, gpd1, adar, nfi, monocistronic, ubq1, mai1, stemloop, sm, aauaaa, cetlf, smg7, myotactin, nucleotides, uaf1, uaf2, transcribe, oma, pkc1a, cstf, u1, acquired

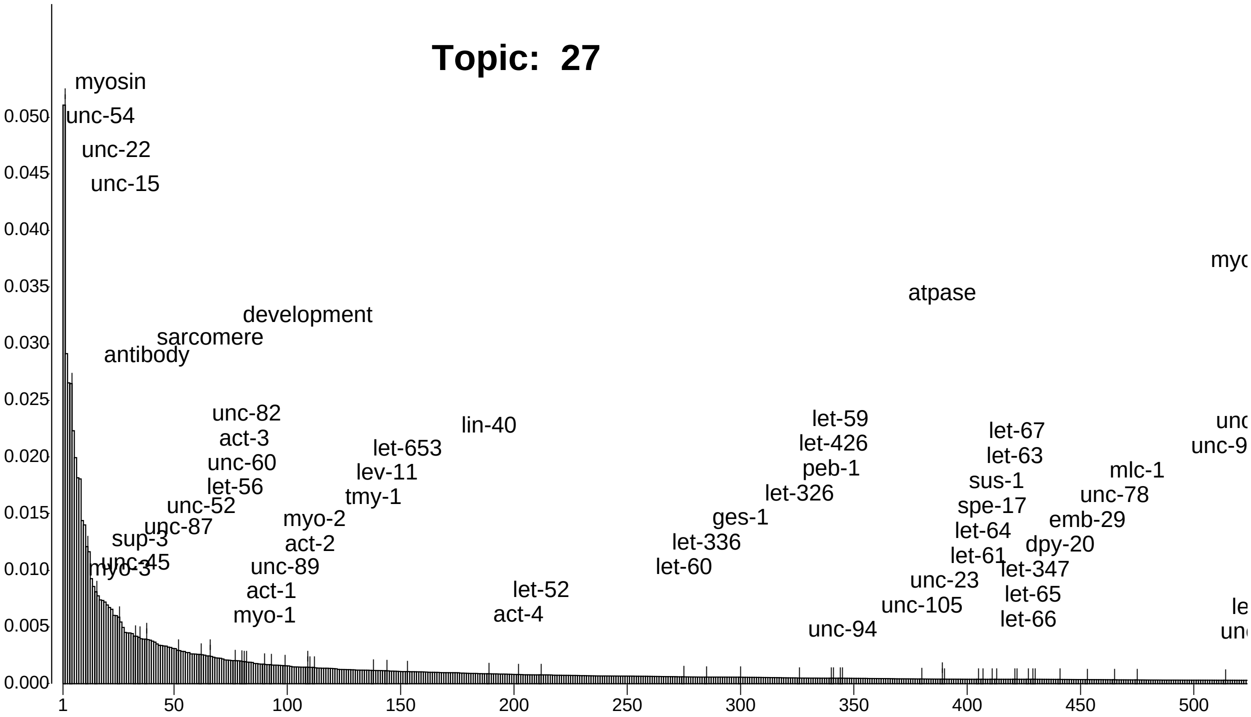

GO-based labels: *MF:* ; *CC:* myosin ii, unconventional myosin, striated muscle thin filament; *BP:* regulation of locomotion, locomotion, negative regulation of locomotion, positive regulation of locomotion, microgametogenesis, abscission, aging **CGC-based labels:** myosin, thick, paramyosin, mhc, rod, unc45, filaments, sup3, unc87, e675, tropomyosin, let56, act3, unc82, myo1, heavychain, unc89, sdf2, e190, peb, act2, subfilament, musculature, chains, diameter, tmy1, lev11, assembled, mhca, let653, sdf9, lie, assemblage, nonunc54, filagenin, finestructure, um, globular, delete, lin40, tubule, organized, sdf8, myofibrillar, positioned, let52, sdf19, rigid, antimyosin, sdf7, waterston, hcdf1, backbone, structures, tubular, multifilament, myosins, crossbridge, c183, reactivity, sdf10, immunological, edf1

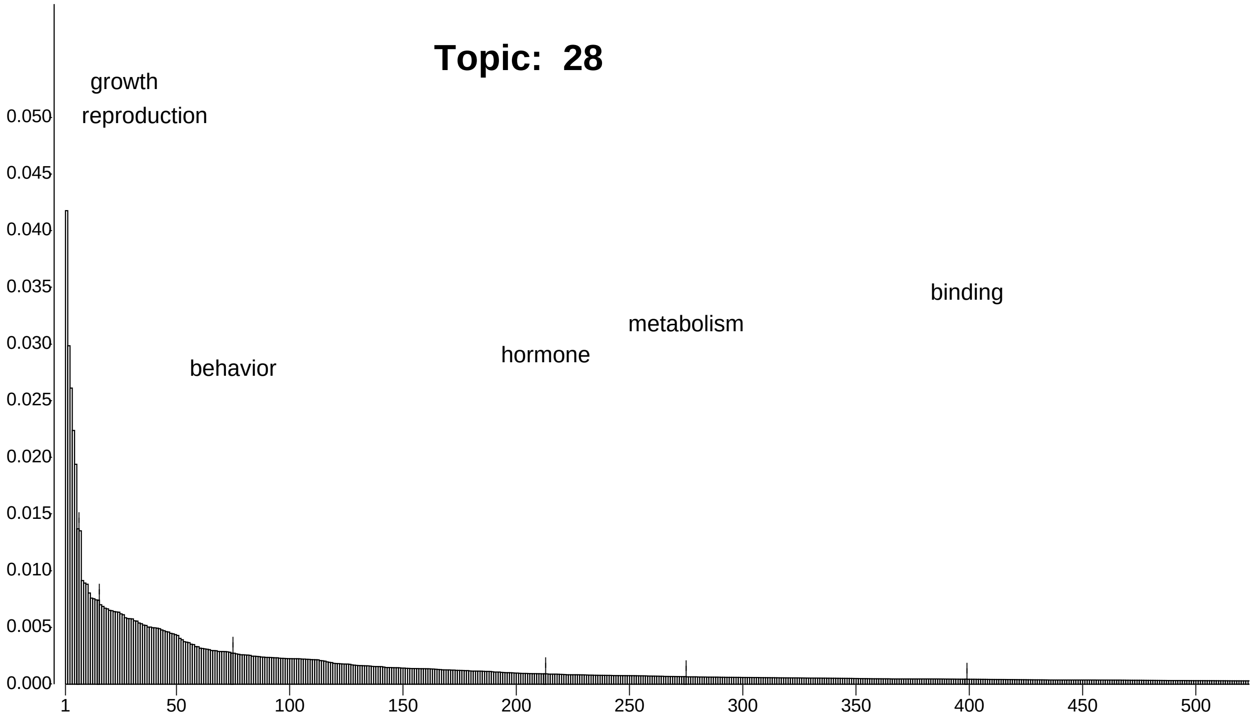

GO-based labels: *MF:* ; *CC:* ; *BP:* adult feeding behavior, adult behavior **CGC-based labels:** decreased, nematicidal, mushroom, ethanol, bacillu, treated, maximal, dauerlarvae, microgram, reversible, compost, threshold, thuringiensi, agaricu, pigment, microm, marked, mycelial, bisporu, pdk, paralysi, recover, decreasing, concentrations, prevented, mug, prolonged, lipofuscin, dmso, temperatures, fluorescen, cultivated, nms, centrophenoxine, amidantel, reached, sulfhydryl, fed, efficacy, nematicide, account, recovering

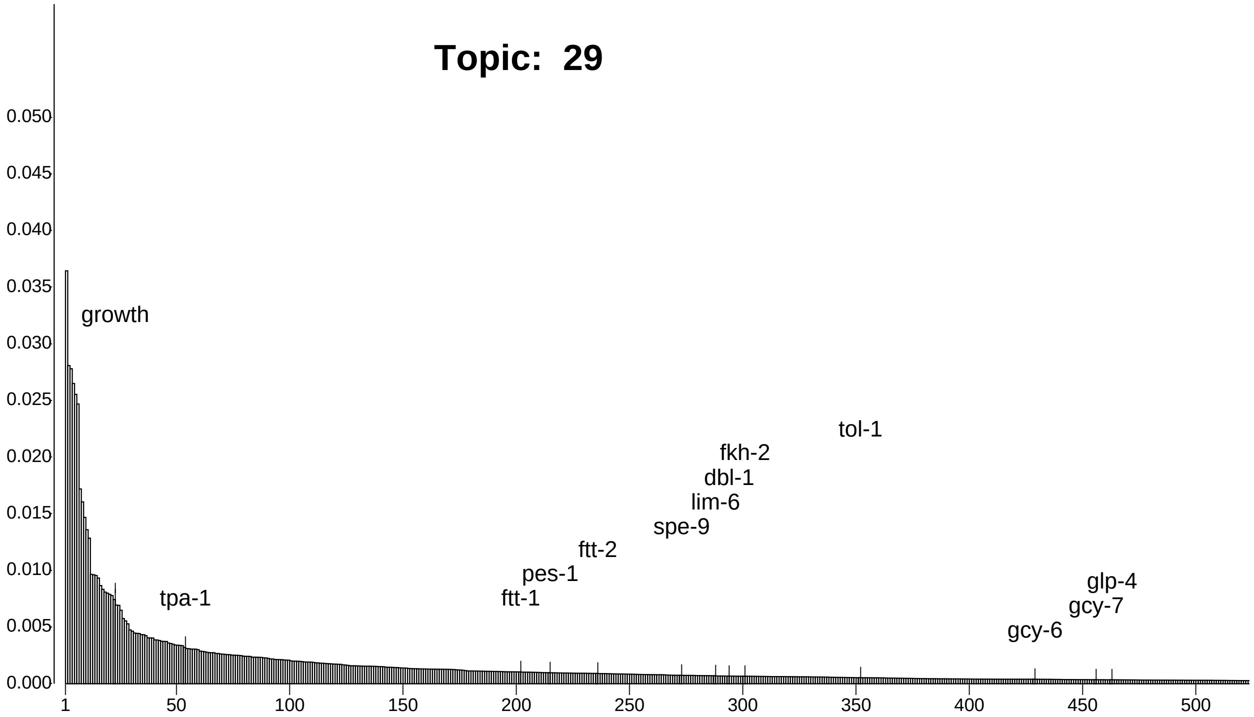

GO-based labels: *MF:* ; *CC:* ; *BP:* **CGC-based labels:** handedness, vary, hypothesi, fit, tpa1, brood, duplicate, comparing, deleteriou, substantial, constraint, fitness, aser, striking, redundancy, evident, experimentally, innate, asel, toll, million, faster, equal, hypothese, logistic, phyla, proportion, reproduce, equation, symmetry, architecture, exponential, immunity, tpa, complexity, frequently, belonging, populations, isogenic, ftt1, fewer, extent, weibull, sinistral, b3, speciation, ftt2, argue

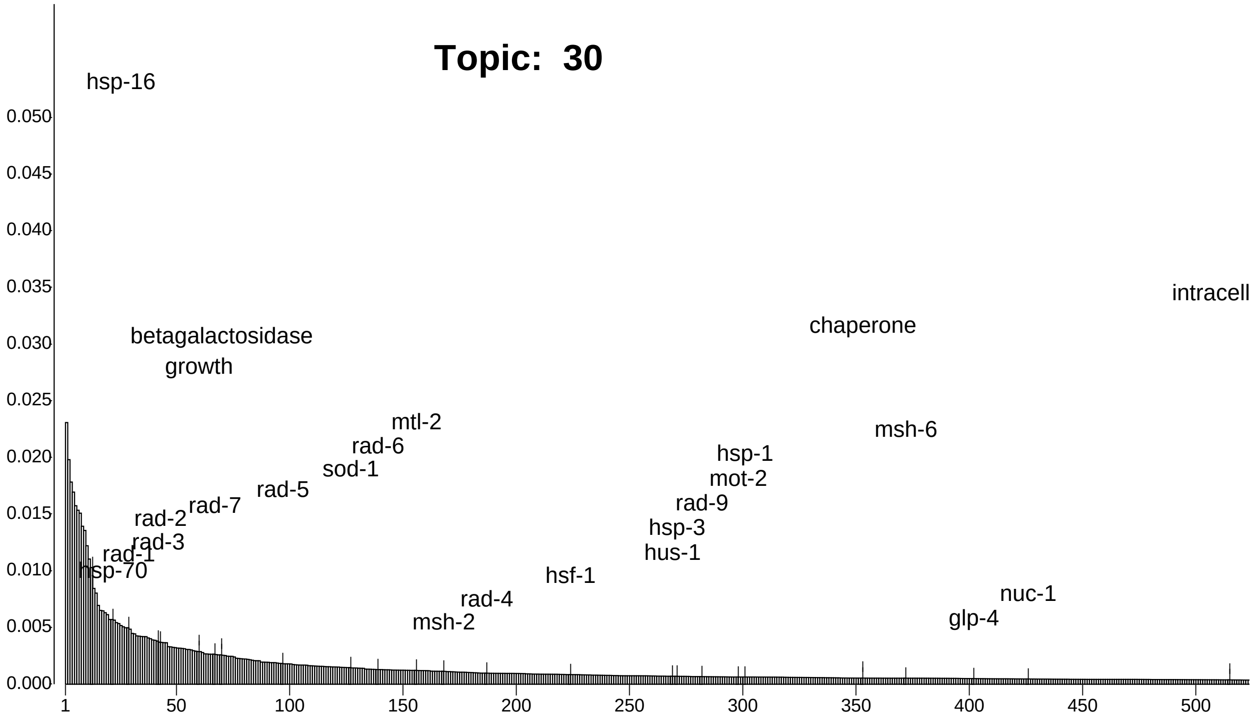

GO-based labels: *MF:* ; *CC:* mitochondrial derivative; *BP:* **CGC-based labels:** hsp16, heatshock, hsp70, rad1, cadmium, rad3, rad2, rad, inducible, hsp, microwave, rad7, surfactant, stressinducible, rad5, tolerance, exogenou, radiationsensitive, hypersensitive, endpoint, cd2, deposit, acute, stressor, sublethal, degreesc, fudr, fungicide, assessment, histochemical, heating, msh2, toxicological, pc72, enhancement, hsbp1, mancozeb, abeta, pluronic, magnetic, pb, zn, slightly, monitor, engineered, introduced, hypersensitivity, ed50, hsf1, ozonation, levels, irradiated, assess, monitoring, top3alpha, emf, nonimmobilizer, hsp70f

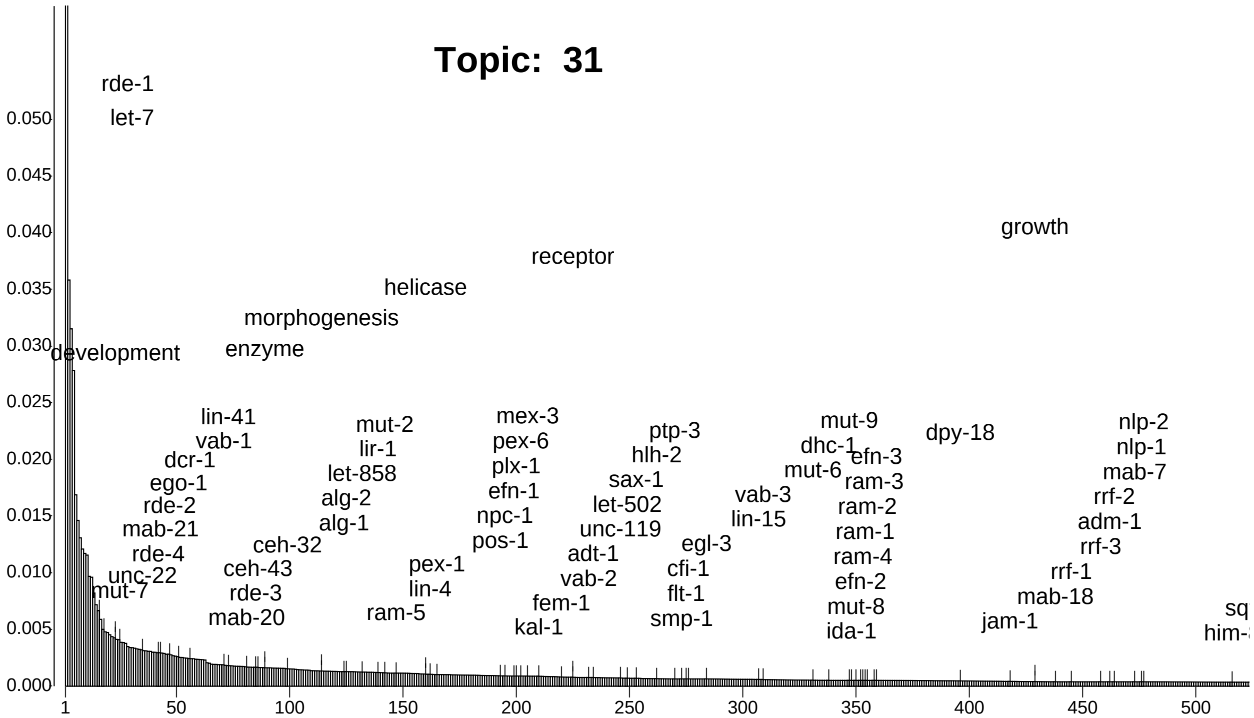

GO-based labels: *MF:* 3'-5' exodeoxyribonuclease, exodeoxyribonuclease; *CC:* mitochondrial derivative; *BP:* abscission, aging **CGC-based labels:** ray, silencing, dsrna, rde1, mut7, antisense, rde4, mab21, trigger, rde2, cosuppression, sirna, syndrome, dcr1, strna, mirna, vab1, rays, mab20, rde3, ceh43, rde, silenced, ceh32, semaphorin, triggered, ephrin, asm, alg1, alg2, efn, silence, let858, dicer, ram5, pex, kal, lgr, sequencespecific, eph, pex1, dcr, microrna, npc1, pex6, plx1, nep, ram, efn1, peroxin, humans, adamt, kal1, dopaminergic, interfering, systemic, pc2, nlp, ida, c11h1, sad, vab2, phogrin, adt1, remodeling

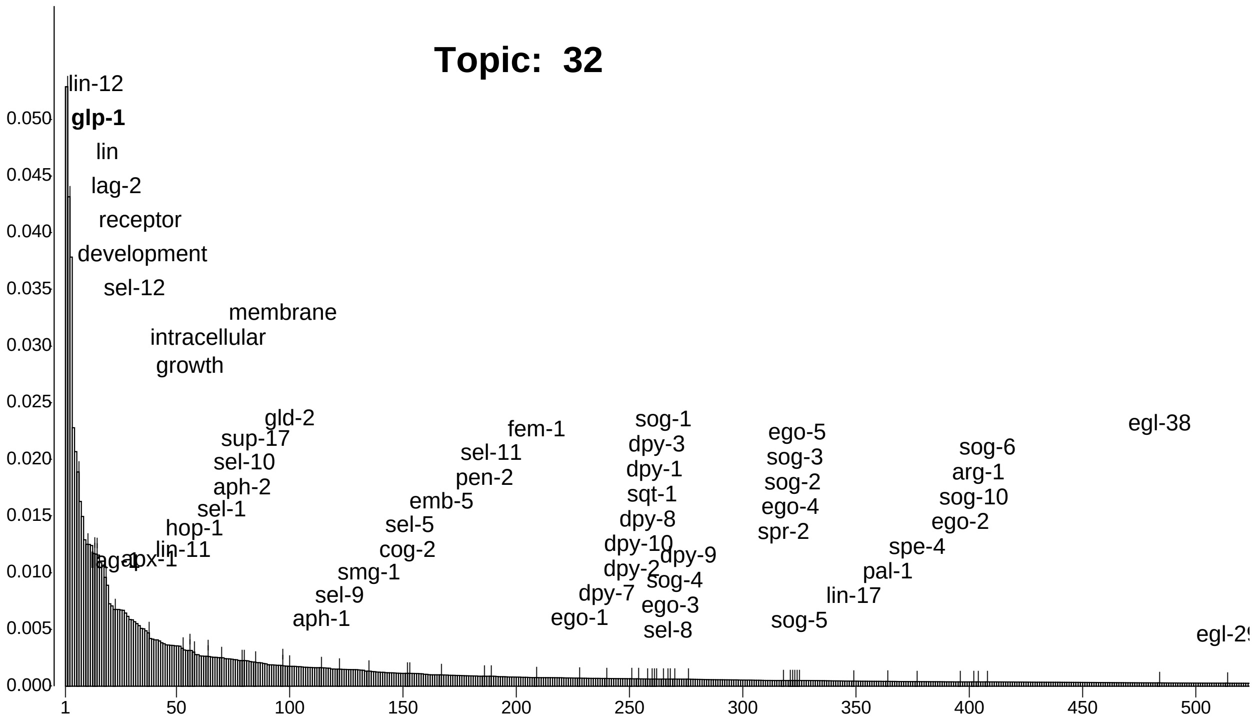

GO-based labels: *MF:* phosphatidylinositol 3-kinase class i; *CC:* mitochondrial outer membrane, apical part of cell, basal part of cell, mitochondrial membrane, peroxisomal membrane; *BP:* hexose mediated signaling, carbohydrate mediated signaling, sucrose mediated signaling, abscisic acid mediated signaling, hexokinase-dependent signaling, hexokinase-independent signaling, regulation of abscisic acid mediated signaling, abscission, aging, flocculation 'sensu saccharomyces' **CGC-based labels:** notch, presenilin, lag2, glp, sel12, sel, uterine, lag1, lag, ac, hop1, alzheimer, sel1, vu, sel10, aph2, sup17, wastewater, nicastrin, gld2, apx, dsl, aph1, sel9, decisions, intrinsic, ank, reciprocal, cdc10, serrate, sel5, uterinevulval, cog2, initially, factorlike, familial, receiving, utse, sel11, aph, fad, hop, ppp, pen2, presumptive, aaa, extrinsic, choose, wwtp, oz112gf, discharge, uv1, presenilins, topology, su, kimble

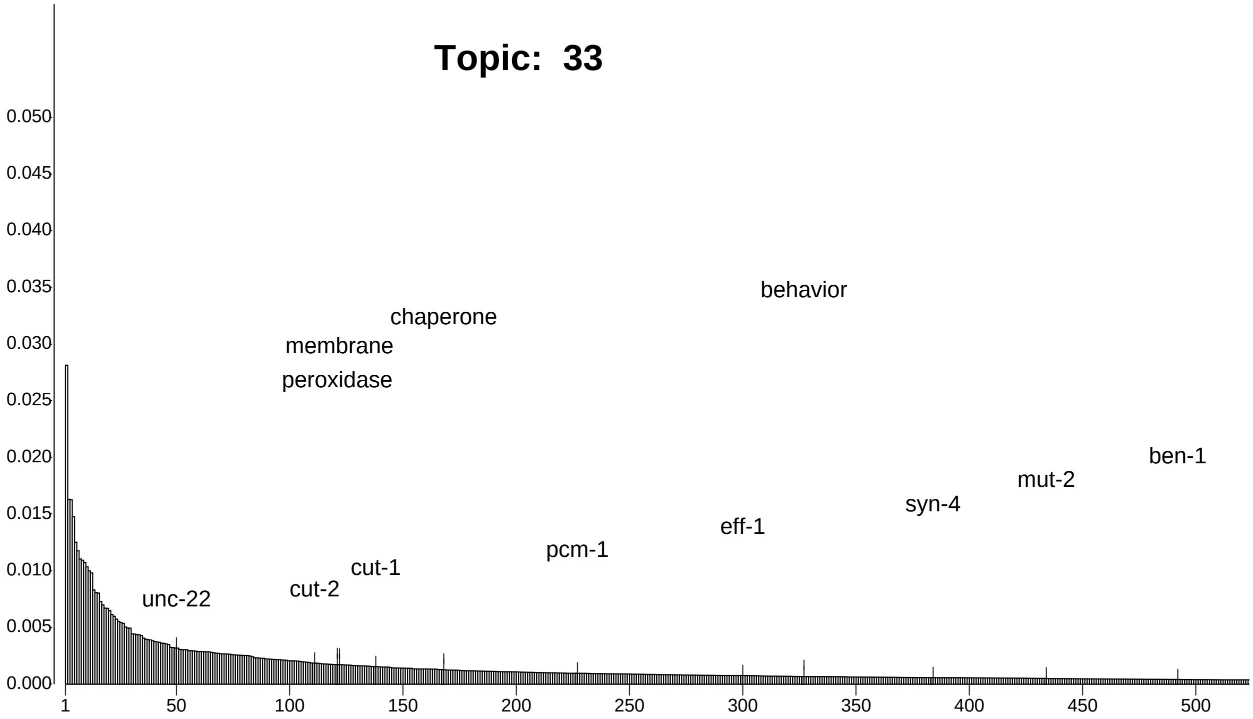

GO-based labels: *MF:* ; *CC:* mitochondrial outer membrane, mitochondrial membrane, peroxisomal membrane; *BP:* **CGC-based labels:** cut, crosslinking, mutagen, formaldehyde, efficiently, cuticlin, damaged, enu, methyltransferase, mutagenic, aggregate, turnover, mutagenized, isoaspartyl, complexes, balanced, spectrum, tmp, prerequisite, aggregation, smhsp, cut2, polyglutamine, deletions, probability, pulsation, tocotrienol, monomeric, possibly, rearrangements, sulfonate, ls, trimethylpsoralen, iron, alphacrystallin, velocity, employed, threefold, fluence, macromolecule, charged, processive, catalyzed, methanesulfonate, shsp, stock, crosslinked, true, oligomeric, radiationinduced, pcm1, dityrosine, frozen, retained, aberrant, accumulated, inviable, compete

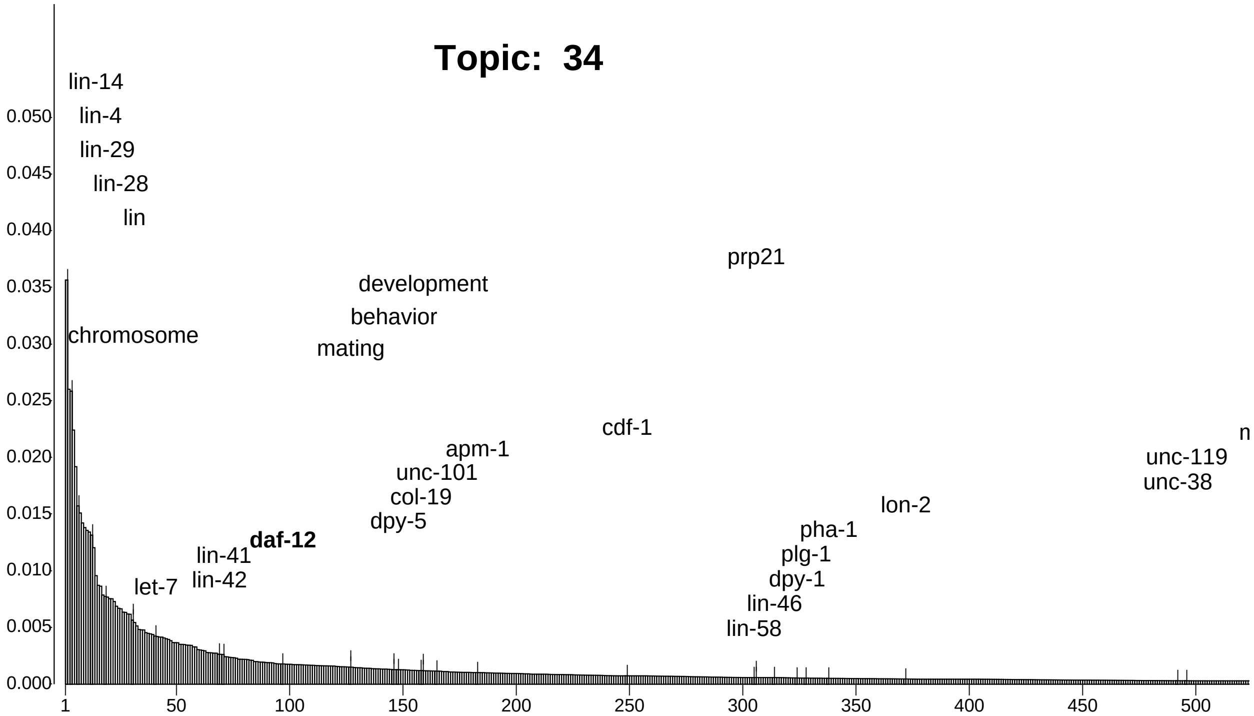

GO-based labels: *MF:* ; *CC:* mitochondrial derivative; *BP:* adult feeding behavior, adult behavior, abscission, aging **CGC-based labels:** lin14, lin29, heterochronic, lin28, cross, polymorphism, trait, de, qtl, french, inbred, polymorphic, lin42, chez, la, snp, precociou, parent, traits, libre, downregulation, du, recombinantinbred, variance, inbreeding, maupa, lifehistory, f2, lines, rflp, ovotesti, adultspecific, remodelling, apm1, heritability, renaturation, percent, interstrain, epistatic, les, rfld, crosses, genetique, heterosi, zn2, selfing, pleiotropy, threefactor, exceptional, dissected, hundred, nullo, highresolution, race, unrelated, clathrinassociated, ark, heterochrony, cdf1, dds

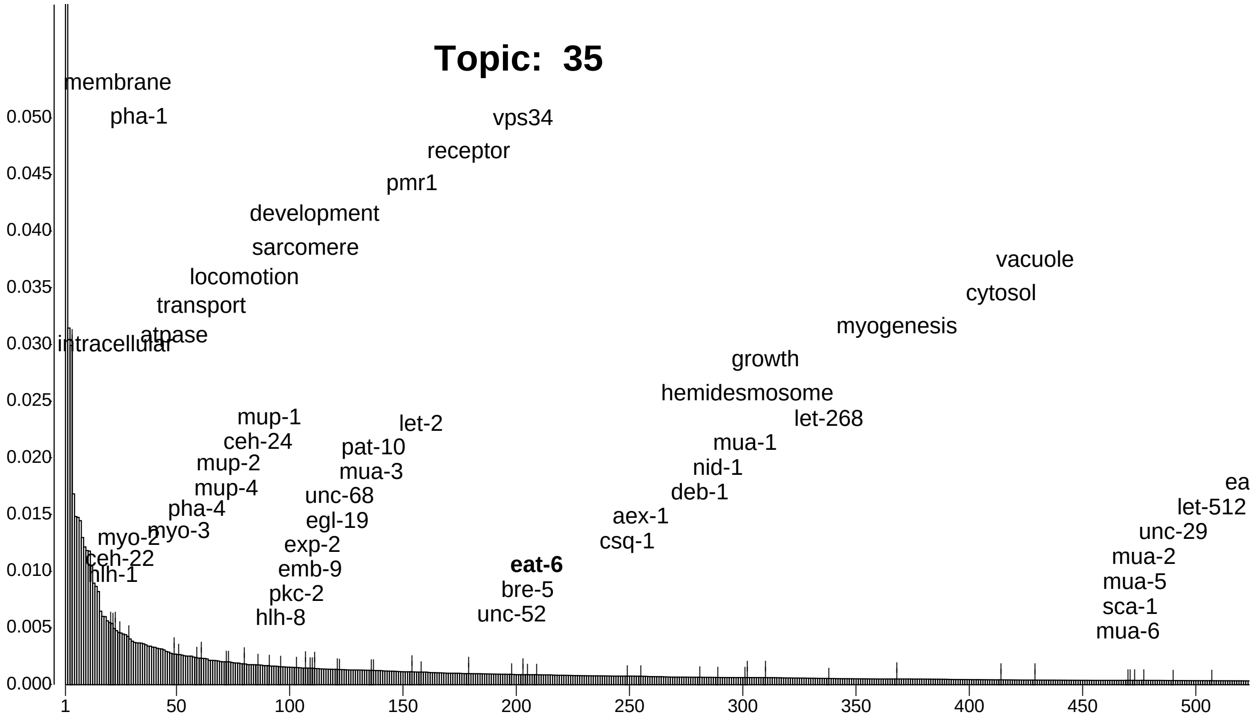

GO-based labels: *MF:* ; *CC:* mitochondrial outer membrane, mitochondrial membrane, peroxisomal membrane, apical part of cell, basal part of cell, striated muscle thin filament; *BP:* ethylene mediated signaling pathway, osmosensory signaling pathway via two-component system, regulation of locomotion, two-component signal transduction system 'phosphorelay', negative regulation of transcription by pheromones, positive regulation of transcription by pheromones, regulation of transcription from pol i promoter mitotic, negative regulation of locomotion, positive regulation of locomotion, ethylene mediated signaling pathway 'induced systemic resistance', ethylene mediated signaling pathway 'jasmonic acid/ethylene dependent systemic resistance', mapkkk cascade 'osmolarity sensing', negative regulation of transcription from pol i promoter mitotic, positive regulation of transcription from pol i promoter mitotic, abscission, aging, locomotion, negative regulation of transcription from pol ii promoter by pheromones, positive regulation of transcription from pol ii promoter by pheromones **CGC-based labels:** basement, pha1, ceh22, troponin, myod, cemyod, m3, musclespecific, bt, subelement, ldh, myogenic, ceh, mup4, mup2, myogenesi, ceh24, mup1, nonstriated, calciumbinding, hlh8, membranes, lumen, pkc2, sphincter, exp2, coupling, bm, exp, nk, bulb, mua3, pat10, obliquely, store, calsequestrin, pmr1, voltagegated, let2, betaspectrin, coordination, resting, mup, sarcoplasmic, serca, i5, mua, anal, excitable, cavity, organspecific, cardiac, elevation, tnt, rer, pump, nkx2, vps34, bre5, stable, hsp25, junctional, heart, marginal, csq1, ceslo2, csq, galbeta1, lysyl, isthmu

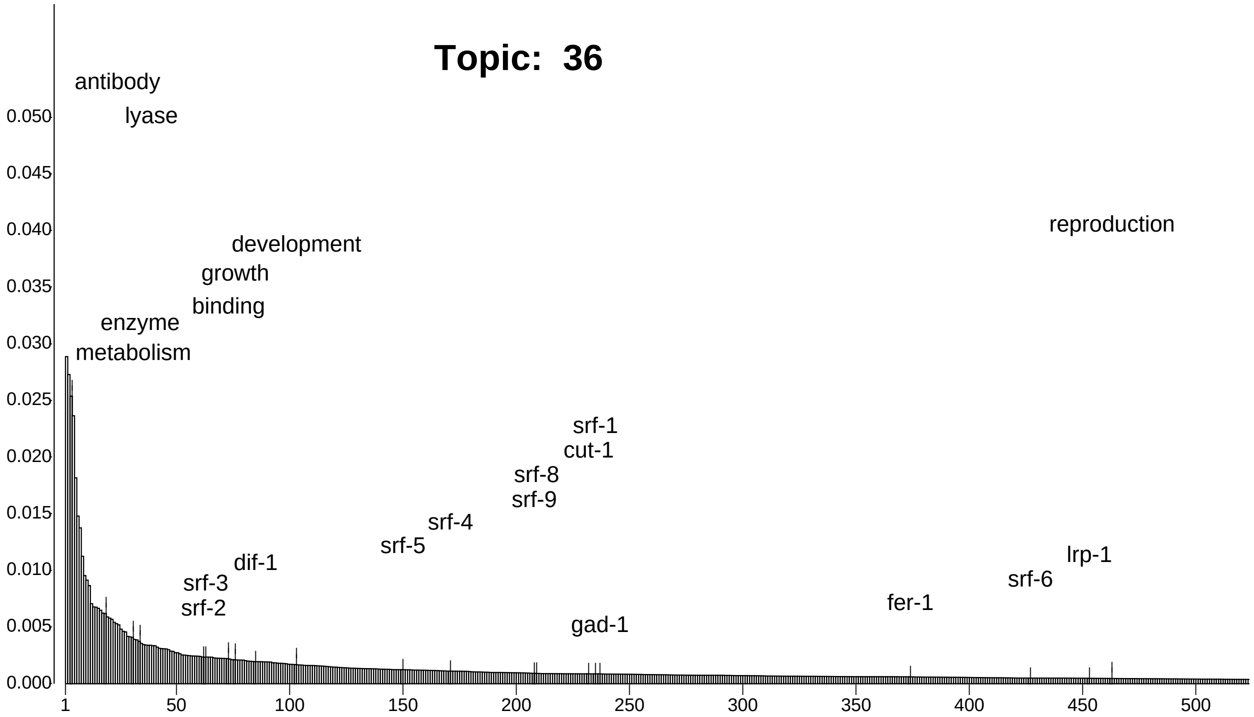

GO-based labels: *MF:* amino acid binding, calcium ion binding; *CC:* ; *BP:* drug metabolism, abscission, aging **CGC-based labels:** isocitrate, lyase, immunofluorescence, lumbricoide, glh, glyoxylate, fixation, srf2, srf3, malate, amine, antigenic, dif1, antisera, sitosterol, dehydrocholesterol, srf, visualized, dauerlarva, mount, fine, epicuticle, coat, srf5, western, take, peak, visualization, ferritin, dealkylation, srf4, densebody, cephalobu, propagated, campesterol, lrp, diminution, wga, itaconate, electrondense, thirdstage, lesser, recognized, labelling, srf8, srf9, homogenate, enol, srf1, configuration, gad1, blue, metabolite, ly

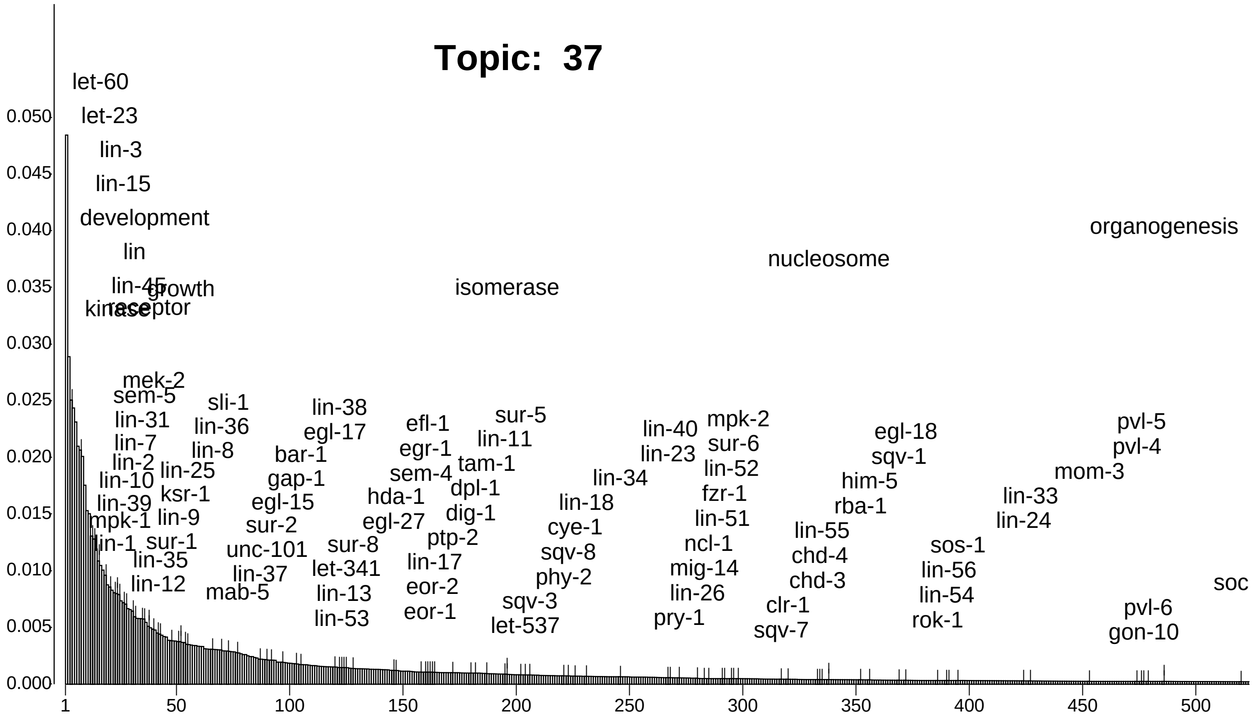

GO-based labels: *MF:* ; *CC:* mitochondrial derivative; *BP:* ethylene mediated signaling pathway, osmosensory signaling pathway via two-component system, hexose mediated signaling, two-component signal transduction system 'phosphorelay', carbohydrate mediated signaling, negative regulation of transcription by pheromones, positive regulation of transcription by pheromones, regulation of transcription from pol i promoter mitotic, ethylene mediated signaling pathway 'induced systemic resistance', ethylene mediated signaling pathway 'jasmonic acid/ethylene dependent systemic resistance', mapkkk cascade 'osmolarity sensing', negative regulation of transcription from pol i promoter mitotic, positive regulation of transcription from pol i promoter mitotic, sucrose mediated signaling, abscisic acid mediated signaling, hexokinase-dependent signaling, hexokinase-independent signaling, regulation of abscisic acid mediated signaling, negative regulation of transcription from pol ii promoter by pheromones, positive regulation of transcription from pol ii promoter by pheromones, regulation of fgf receptor signaling pathway, regulation of embryonic development rate, microgametogenesis, negative regulation of embryonic development rate, positive regulation of embryonic development rate, fgf receptor signaling pathway, abscission, aging, regulation of transcription by pheromones, regulation of transcription mitotic, drug metabolism, negative regulation of transcription mitotic, positive regulation of transcription mitotic, negative regulation of transcription from pol ii promoter mitotic, negative regulation of transcription from pol iii promoter mitotic, positive regulation of transcription from pol ii promoter mitotic, positive regulation of transcription from pol iii promoter mitotic, activation of mapk 'osmolarity sensing', activation of mapkk 'osmolarity sensing', activation of mapkkk 'osmolarity sensing' **CGC-based labels:** vulval, ras, let60, lin3, lin15, lin45, lin1, mpk1, vpc, lin10, lin2, lin7, lin31, mek2, lin35, sur1, lin9, ksr1, lin25, multivulva, rasmediated, lin8, lin36, vulvaless, lin37, competence, sur2, gap1, synmuv, p8, basolateral, lin38, lin53, lin13, let341, sur8, equivalence, nurd, pas, hda1, cellsignaling, e2f, sur, p12, egr1, efl1, eor1, eor2, redundantly, ptp2, dig1, muv, dpl1, mpk, tam1, invagination, graded, nonvulval, multipotent, antagonize, sur5, let537, sqv3, dp, phy2, competent, sqv8, cye1, lin18, antiallatal, lin34, ppi, hif

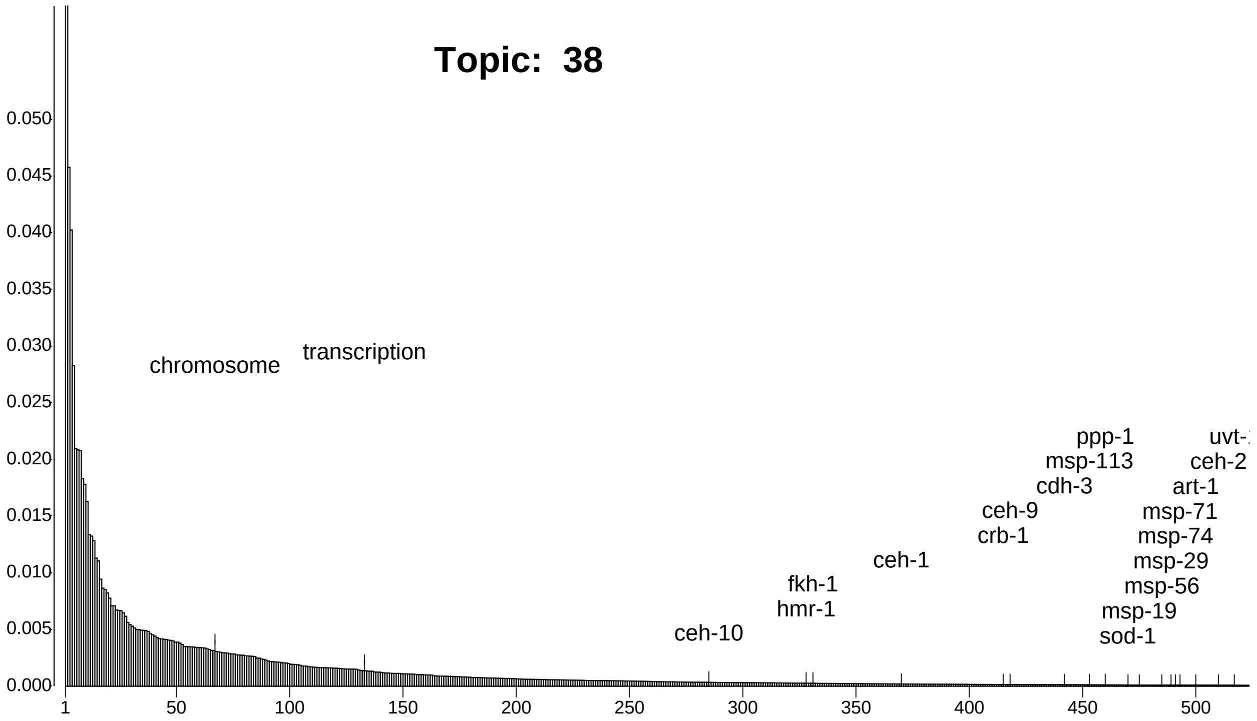

GO-based labels: *MF:* ; *CC:* ; *BP:* **CGC-based labels:** multigene, cadherin, pseudogene, diverged, distantly, duplicated, existence, irp, intergenic, dlg, families, dispersed, ancestral, pairs, raise, sea, basepair, urchin, clustered, imply, members, interspecy, raising, remarkably, innexin, synapsin, connexin, arranged, metazoans, strikingly

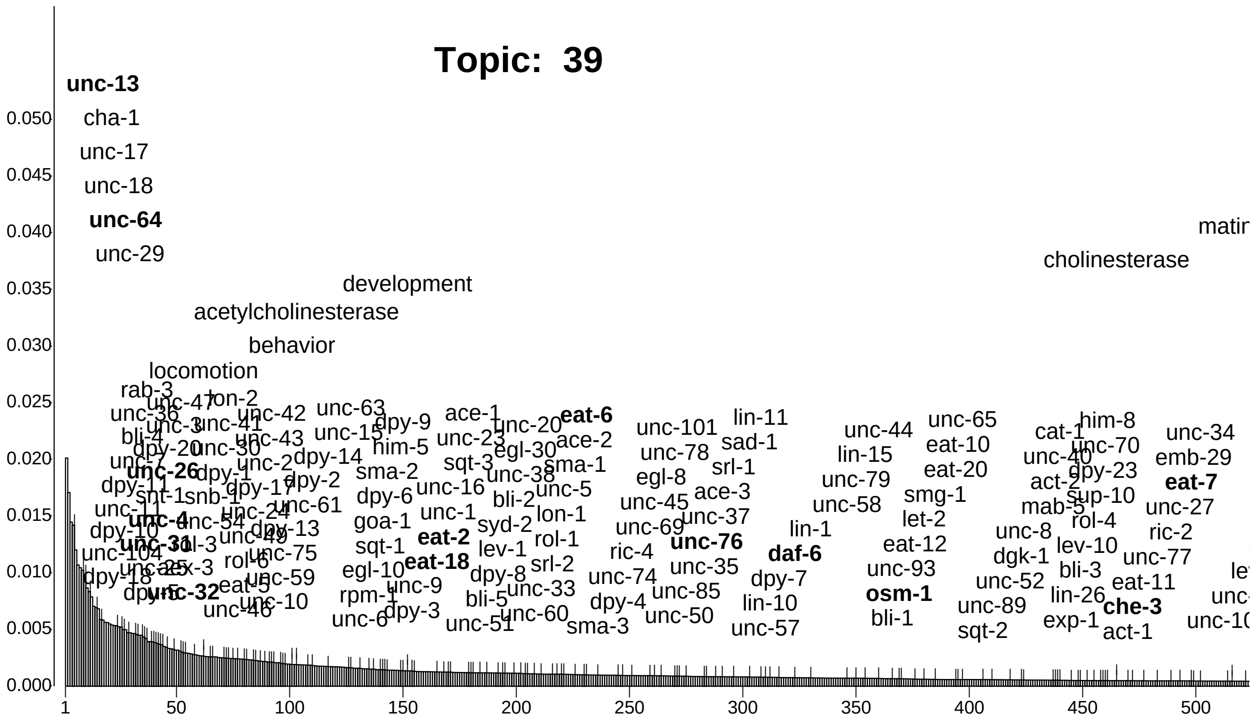

GO-based labels: *MF:* exodeoxyribonuclease; *CC:* mitochondrial derivative; *BP:* regulation of locomotion, ethylene mediated signaling pathway, osmosensory signaling pathway via two-component system, two-component signal transduction system 'phosphorelay', locomotion, negative regulation of locomotion, positive regulation of locomotion, negative regulation of transcription by pheromones, positive regulation of transcription by pheromones, regulation of transcription from pol i promoter mitotic, ethylene mediated signaling pathway 'induced systemic resistance', ethylene mediated signaling pathway 'jasmonic acid/ethylene dependent systemic resistance', mapkkk cascade 'osmolarity sensing', negative regulation of transcription from pol i promoter mitotic, positive regulation of transcription from pol i promoter mitotic, hexose mediated signaling, aspartate transport, glutamate transport, adult feeding behavior, negative regulation of transcription from pol ii promoter by pheromones, positive regulation of transcription from pol ii promoter by pheromones, carbohydrate mediated signaling, drug transport, eye pigment precursor transport, 2-keto-3-deoxygluconate transport, acidic amino acid transport, adult behavior, abscission, aging, thermoregulation, fat body metabolism 'sensu insecta' **CGC-based labels:** cha1, unc17, unc18, syntaxin, roller, dpy18, unc11, dpy11, unc36, rab3, snt1, unc26, aex3, rol3, snb1, ndf24, dpy1, unc41, lon2, unc46, eat5, dpy17, unc10, mndf30, unc59, unc75, ndf25, edf2, synaptobrevin, edf18, unc61, vesicles, snare, synaptotagmin, dpy14, mdf7, sdf35, mndf89, mndf1, exocytosi, rpm1, dpy6, edf3, mndf41, mndf88, dpy9, dpy3, ndf19, madf4, eat18, mndf68, ndf23, unc16, cover, unc23, edf6, bli5, rim, sdf28, aex, dpy8, syd2, sdf20, mndf4, bli2, aldicarb, unc20, ndf27, hdf9, ndf40, edf4, mc, srl2, e937, sdf6, rol1, synapses, priming, sma1, mndf61, qdf3, sdf26, noncomplementation, mndf111, dpy4, ardf1, superbu, ric4

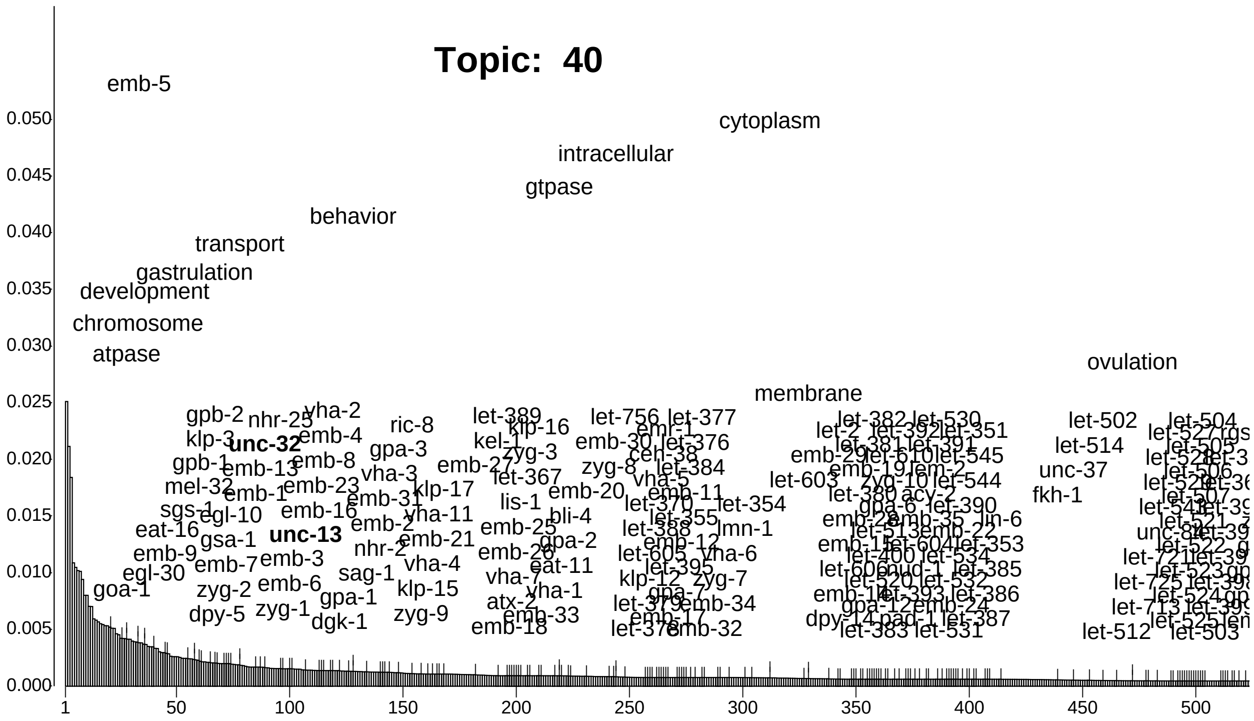

GO-based labels: *MF:* ; *CC:* dynein, apical part of cell, basal part of cell; *BP:* ethylene mediated signaling pathway, osmosensory signaling pathway via two-component system, hexose mediated signaling, two-component signal transduction system 'phosphorelay', carbohydrate mediated signaling, regulation of locomotion, negative regulation of transcription by pheromones, positive regulation of transcription by pheromones, regulation of transcription from pol i promoter mitotic, sucrose mediated signaling, abscisic acid mediated signaling, adult feeding behavior, ethylene mediated signaling pathway 'induced systemic resistance', ethylene mediated signaling pathway 'jasmonic acid/ethylene dependent systemic resistance', mapkkk cascade 'osmolarity sensing', negative regulation of transcription from pol i promoter mitotic, positive regulation of transcription from pol i promoter mitotic, hexokinase-dependent signaling, hexokinase-independent signaling, regulation of abscisic acid mediated signaling, locomotion, abscission, aging, adult behavior, negative regulation of transcription from pol ii promoter by pheromones, positive regulation of transcription from pol ii promoter by pheromones, microgametogenesis **CGC-based labels:** rgs, envelope, akap, emb, ran, heterotrimeric, lamin, eat16, klp, goa, sgs1, mel32, gpb1, celamin, gpb, klp3, gpb2, gsa1, zyg2, emb7, nckx, permissive, proteolipid, emerin, pde, shmt, emb1, emb13, hdf6, nhr25, rii, tsp, emb3, emb6, man1, emb4, emb8, emb16, emb23, ceemerin, vha2, nonpermissive, dgk1, gpa1, adenylyl, sag1, nhr2, vha3, ggl, emb2, emb31, gpa3, ric8, strict, klp15, klp17, ima, hdp13, vha4, emb21, ataxin2, vha11, runt, temperatureshift, ri, sdf4, emb27, kel1, lis1, celam1, klp16, vacuolartype, atx2, vha, let367, nxf, let389, zyg3, vha7, mex67p, ntf2, ftz, emb18, emb25, emb26, emb33, brf, import, ncd, vha1, eat11, emb20, tethering, zyg8, let756

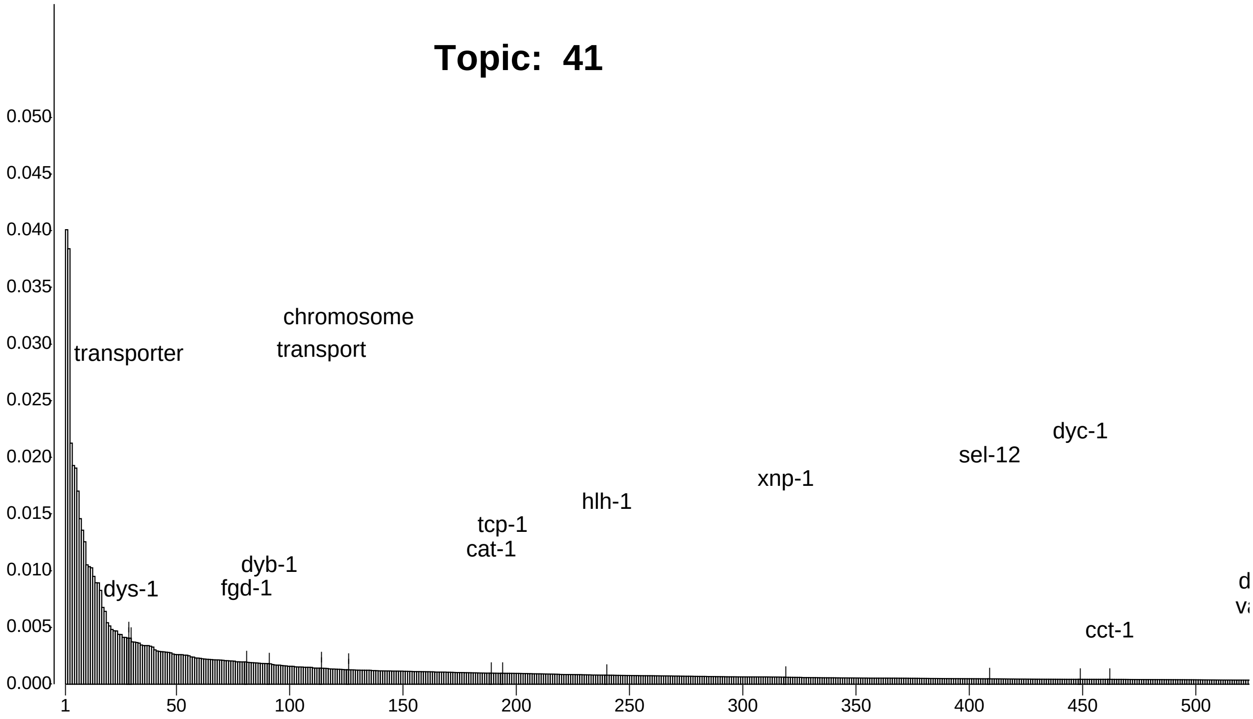

GO-based labels: *MF:* ; *CC:* ; *BP:* **CGC-based labels:** dys1, acedb, dystrophin, proteome, muscular, mb, dystrophy, dystrobrevin, methods, providing, fgd1, dyb1, construction, comprehensive, completed, world, annotated, list, assigned, improved, full, obtaining, highthroughput, progressive, traditional, access, roundworm, disorder, scientist, smn, tfg, began, table, databases, practical, starting, outgroup, genomics, amenable, diseases, annotation, cambridge, microarray, glycopeptide, collection, explore, tcp1, chaperonin, duchenne, vacht, catalogue, genomewide, science, make, greatly, thousand, wealth, people, public, tcp, cpb, working, decade, medical, aid, spinal, success, john, technology, knockout, successfully

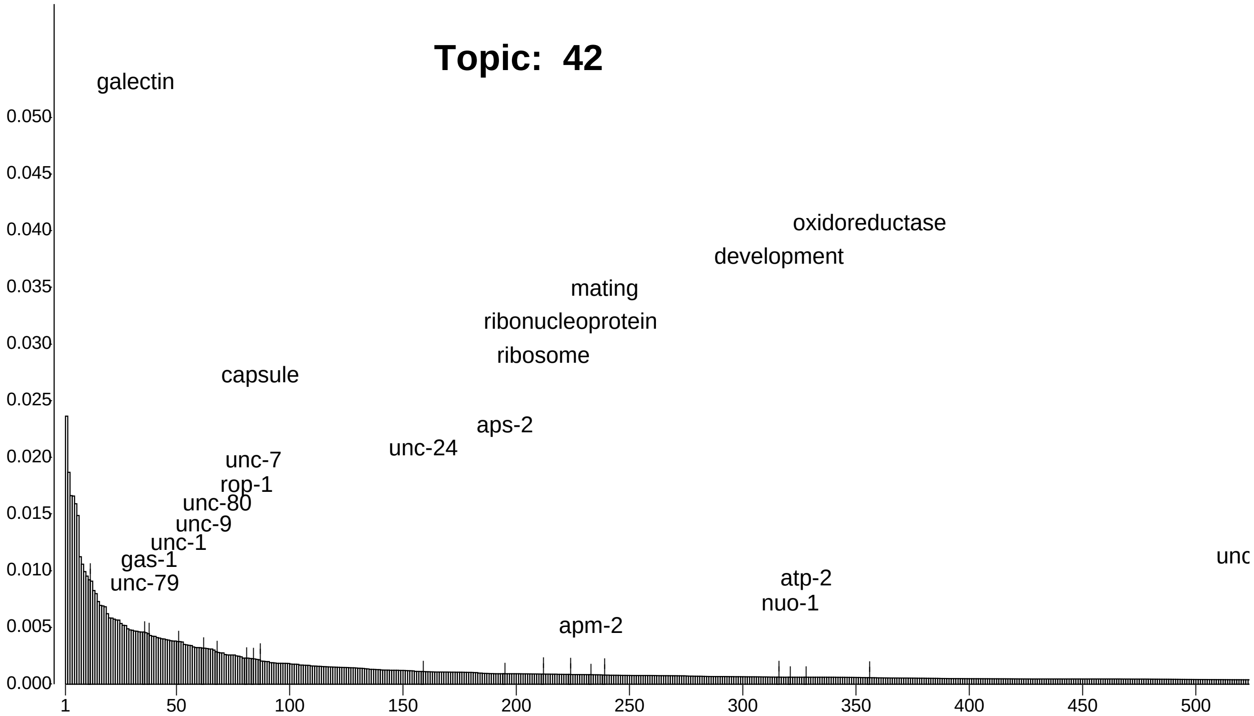

GO-based labels: *MF:* ; *CC:* mitochondrial derivative, cytosolic ribosome 'sensu bacteria', cytosolic ribosome 'sensu eukarya', cytosolic large ribosomal subunit 'sensu bacteria', cytosolic large ribosomal subunit 'sensu eukarya'; *BP:* **CGC-based labels:** anesthetic, halothane, lectin, galectin, rrna, pacificu, pristionchu, character, ro, unc79, gas1, isoflurane, remanei, anesthetics, mtdna, genu, buccal, rdna, unc80, oscheiu, author, ro60, rop1, rhabditida, capsule, punctata, stomatin, enflurane, stereoisomer, cew1, diplenteron, rnp, pacificus, phylogeny, taxa, cephalobina, caenorhabditis, vas, secernentea, anesthesia, vulgari, panagrolaimu, rhabditina, distance, potency, framework, dolichorhabditi, halicti, betagalactosidebinding, ncl, distinguished, immobility, clinical, diplogastridae, rornp, rop, mtsrrna, taxon, postcorpu, osche, mt1, aps2, oesophageal, mrc, distinguish, resolve, ribosome, inferred, pelodera, apm2, proved, consideration, carbohydratebinding

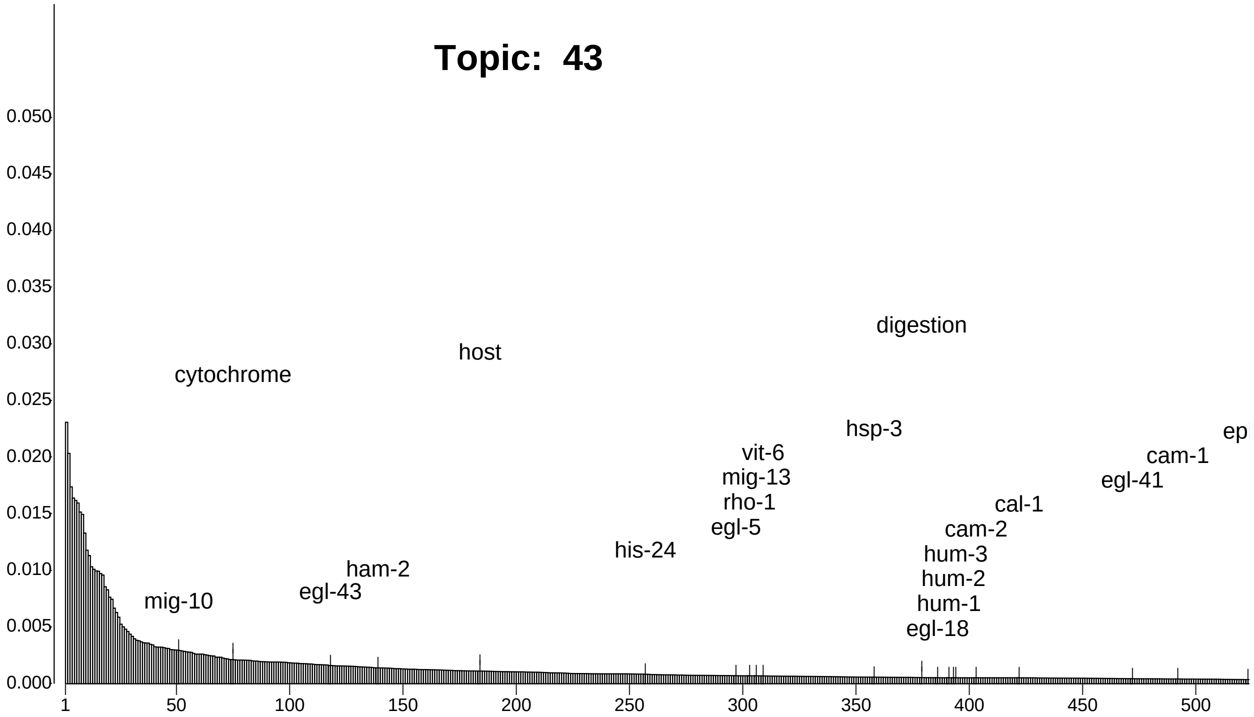

GO-based labels: *MF:* ; *CC:* parasitophorous vacuolar membrane, parasitophorous vacuole, host cell plasma membrane; *BP:* **CGC-based labels:** h1, usage, h2b, bia, h2a, sds, polyacrylamide, diet, h4, mig10, mig, electrophoresis, isoenzyme, calf, filtration, electrophoretic, synonymou, gonaddependent, nonhistone, synthetase, dodecyl, optimal, lysine, yp88, yp115, urea, sulphate, biase, alanine, fibulin1, tes, egl43, fractionation, extraction, dalton, main, resolved, ham2, thymu, silent, cb, doublet, covalent, biased, polypeptides, extracted, can, yp170a, estimated, centrifugation, cleaved, triton, unconventional, fluoroacetic, isotopic, lipoprotein, additionally, fractionated, solubilized, aminoacyltrna, subtype, contextdependent, yp170b, synthetases, hydroxyapatite, daltons, bonds, trend

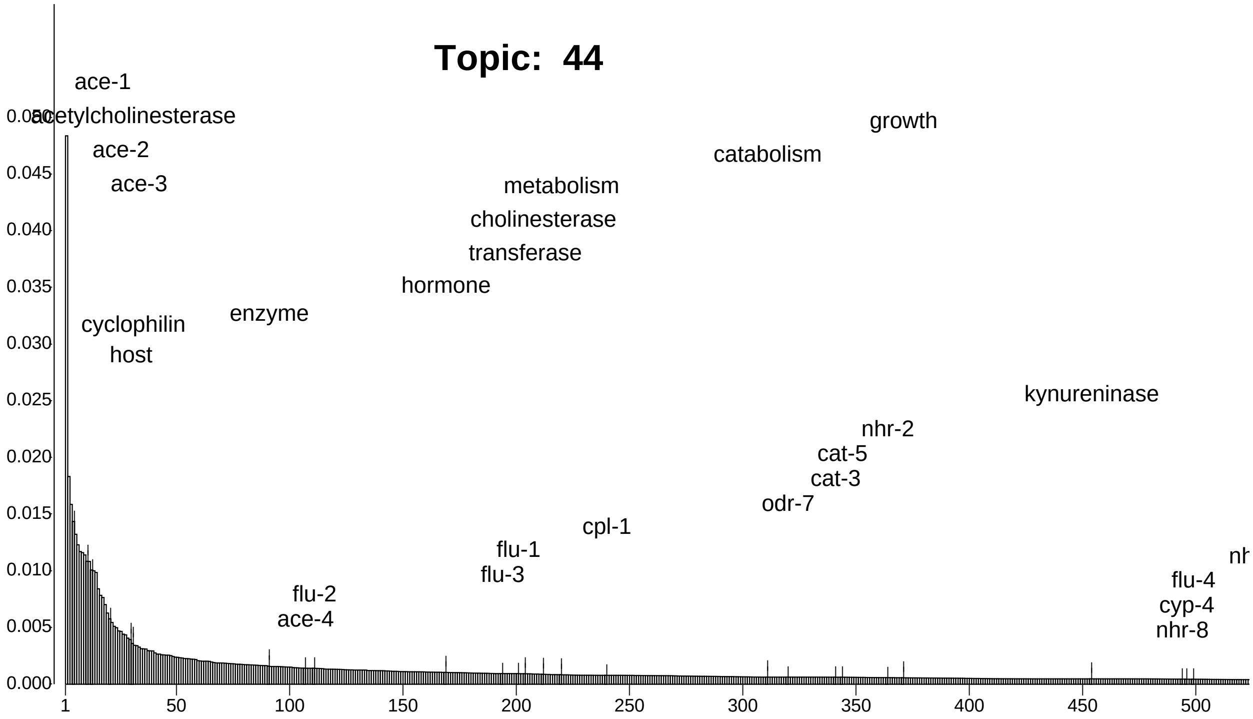

GO-based labels: *MF:* ; *CC:* parasitophorous vacuolar membrane, parasitophorous vacuole, host cell plasma membrane; *BP:* drug metabolism **CGC-based labels:** biomass, ache, ace3, web, helminth, cyclophilin, trophic, root, gst, ace, microbial, fungu, nr, plantparasitic, antibiotic, predator, mif, spp, tannin, capture, trap, microcosm, brugia, infective, incognita, parasites, caninum, habitat, arthrobotry, supply, ace4, microbivore, nematodetrapping, dependence, flu2, parasitism, helminths, top, condensed, fungivore, ml1, ellagitannin, filarial, greatest, hosts, represented, exploited, productivity, bacterivore, graph, ypd, aphelenchoide, fungal, forms, bacterialfeeding, antifungal, acrobeloide, cycloheximide, prey, lbp, predatory, cpl, wormpd, flu3, microbe, agriculture, flu1, qualitative, malayi, nematophagou, penetran, spore, trophiclevel, omnivore, cpl1, gallo, lph, xylophilu, veterinary, minititin, trapping, predaceou, bacteriophagou, ancylostoma

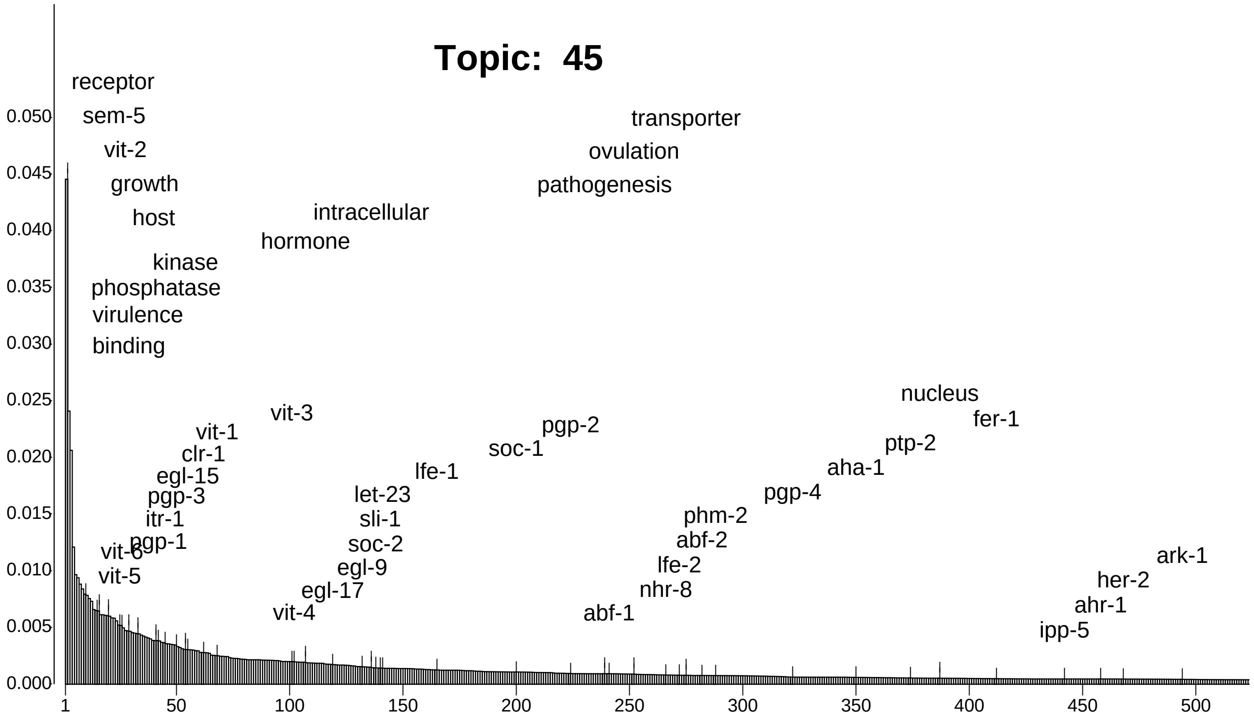

GO-based labels: *MF:* amino acid binding, calcium ion binding, phospholipase d; *CC:* parasitophorous vacuolar membrane, parasitophorous vacuole, host cell plasma membrane, mitochondrial derivative, apical part of cell, basal part of cell; *BP:* regulation of beta 2 integrin biosynthesis, beta 2 integrin biosynthesis, regulation of fgf receptor signaling pathway, integrin biosynthesis, fgf receptor signaling pathway **CGC-based labels:** vitellogenin, fgf, sh3, vit2, pathogen, sh2, aeruginosa, vit5, vit6, glycoprotein, virulence, fibroblast, ip3, src, pgp1, fgfr, itr1, pgp3, clr1, vpe1, vit1, vpe2, pa14, grb2, cbl, typhimurium, lar, glutamatergic, sem, trisphosphate, pseudomallei, vit3, vit4, sli, insp, soc, oncogene, similarly, vit, sos, ahr, rs, egfr, prolinerich, egl9, pathogenesi, soc2, ligandbinding, burkholderia, fkhr, pgp, clr, cyanide, lfe1, sf1, mucd, liprin, salmonella, fkhrl1, pa, estrogen, grampositive, hydrogen, steroid, drk, soc1, opportunistic, glucocorticoid, neurexin, coil, afx, tractable, leucinerich, broad, pgp2, triphosphate, hostpathogen, vcbl, faecali, igfbp, pathogenicity, pyogene, ip3r, pathogenesis, ccbl, abf1, hced, pathogenic, sms

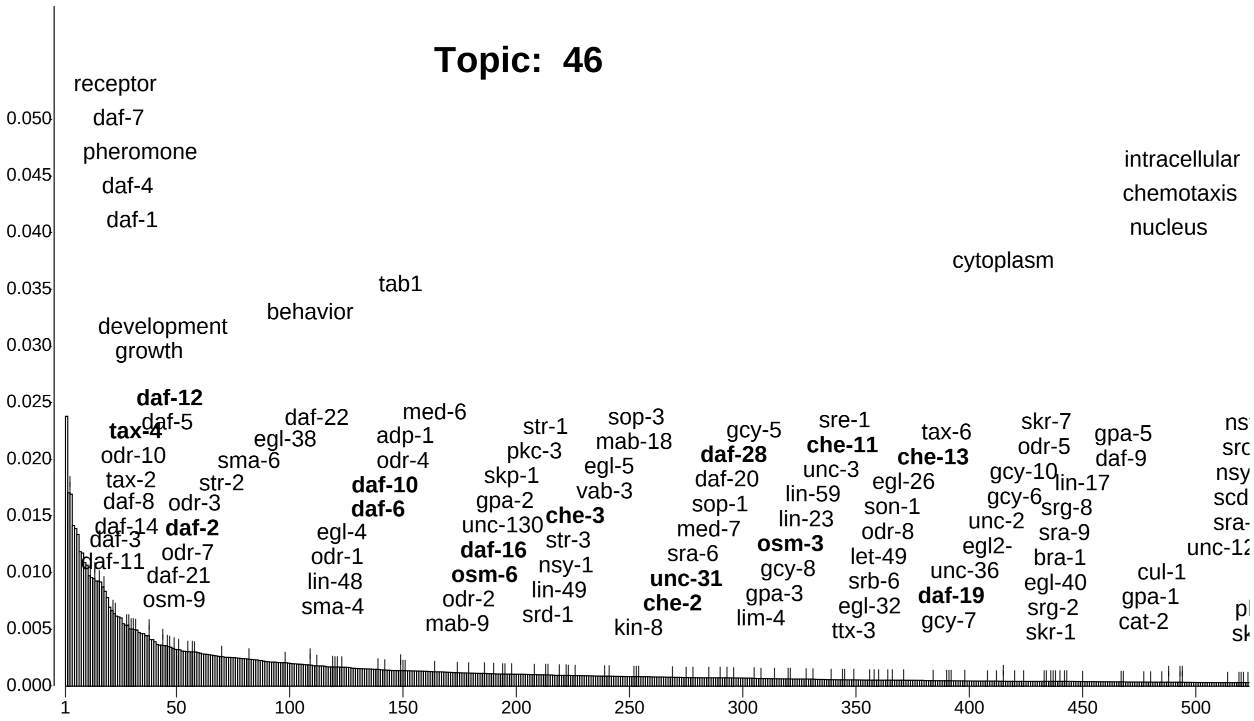

GO-based labels: *MF:* ; *CC:* microsome; *BP:* ethylene mediated signaling pathway, osmosensory signaling pathway via two-component system, two-component signal transduction system 'phosphorelay', negative regulation of transcription by pheromones, positive regulation of transcription by pheromones, regulation of transcription from pol i promoter mitotic, ethylene mediated signaling pathway 'induced systemic resistance', ethylene mediated signaling pathway 'jasmonic acid/ethylene dependent systemic resistance', mapkkk cascade 'osmolarity sensing', negative regulation of transcription from pol i promoter mitotic, positive regulation of transcription from pol i promoter mitotic, negative regulation of transcription from pol ii promoter by pheromones, positive regulation of transcription from pol ii promoter by pheromones, adult feeding behavior, abscission, aging, regulation of transcription by pheromones, regulation of transcription mitotic, adult behavior, negative regulation of transcription mitotic, positive regulation of transcription mitotic, negative regulation of transcription from pol ii promoter mitotic, negative regulation of transcription from pol iii promoter mitotic, positive regulation of transcription from pol ii promoter mitotic, positive regulation of transcription from pol iii promoter mitotic, activation of mapk 'osmolarity sensing', activation of mapkk 'osmolarity sensing', activation of mapkkk 'osmolarity sensing' **CGC-based labels:** olfactory, tgf, pheromone, odorant, daf1, daf11, daf3, daf8, daf14, tax2, odr10, tax4, twitchin, awc, odr, smad, awa, daf5, osm9, daf21, odr7, asi, odr3, cgmp, dafc, tax, mediator, olfaction, str2, cyclic, cka1, guanylyl, hindgut, sma6, egl38, diacetyl, chemosensation, dbl, betalike, nucleotidegated, daf22, discrimination, factorbeta, pax, skr, lin48, odr1, egl4, asg, pax6, dispersal, odorants, tab1, odr4, adp1, med6, odor, dafd, taste, dauerconstitutive, paired, odr2, thermosensation, skp1, dauerinducing, prp4k, synaptogyrin, elicit, overcrowding, str1, srd1, awe, nsy, lin49, tgfb, nsy1, str3, tak1, ig, vab3, sensed, galnactransferase

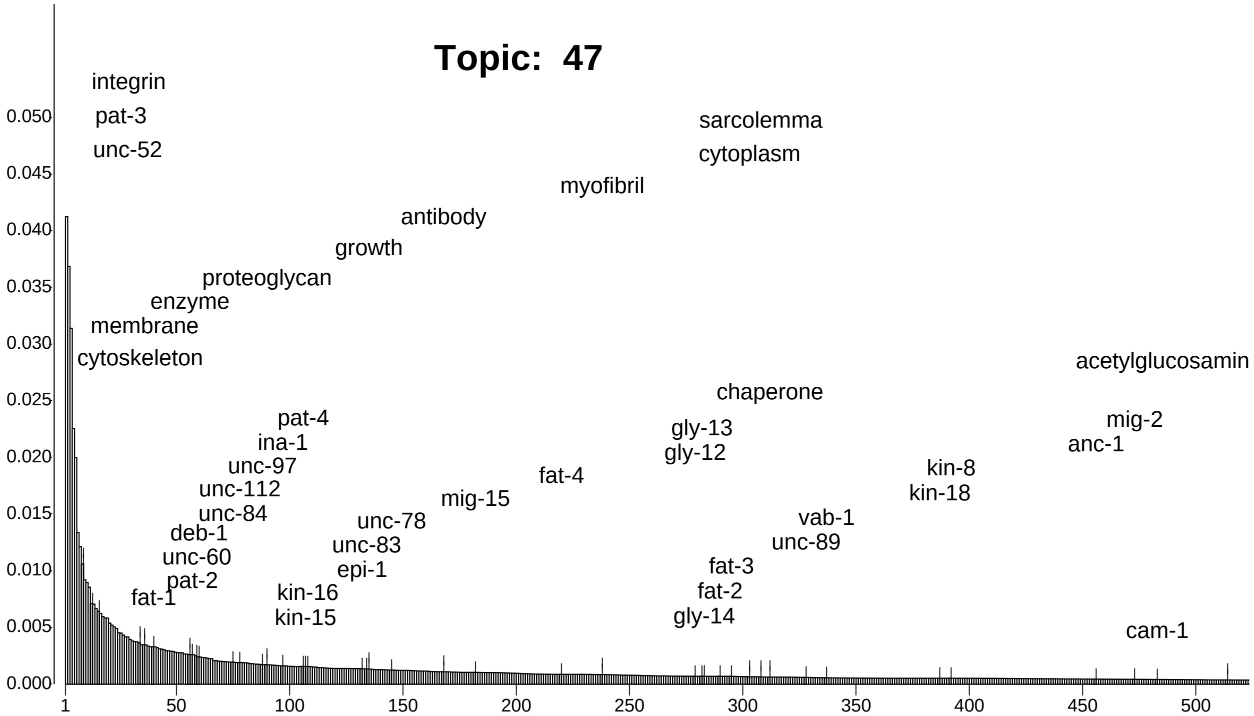

GO-based labels: *MF:* ; *CC:* mitochondrial outer membrane, intermediate filament cytoskeleton, mitochondrial membrane, peroxisomal membrane; *BP:* **CGC-based labels:** fatty, integrin, desaturase, pat3, pufa, n3, ecm, polyunsaturated, vinculin, fat1, perlecan, n6, kettin, cofilin, pat2, actinbinding, deb1, depolymerizing, lad, syk, talin, pat, unc84, cetm, unc112, pek, unc97, proteoglycan, phospholipid, glycan, heterologou, rcm, omega3, pmk, pat4, arachidonic, kin15, kin16, tetratricopeptide, ptdcho, cro1, traf, pmp22, gas3, epa, ilk, laminin, fat, epi1, unc83, polymerization, unc78, elongase, palmitic, eel, eif, glycosyltransferase, acylation, igsf, mig15, omega, betaintegrin, alphaactinin, eicosapentaenoic, severing, hereditary, molecules, glycoconjugate, repertoire, fucose, linoleic, tao1, c01c10, dynamics, ncam, fat4, desaturated, desaturation, shp2, unsaturated, she4p, wd, disorganized, myofibril, repeatcontaining

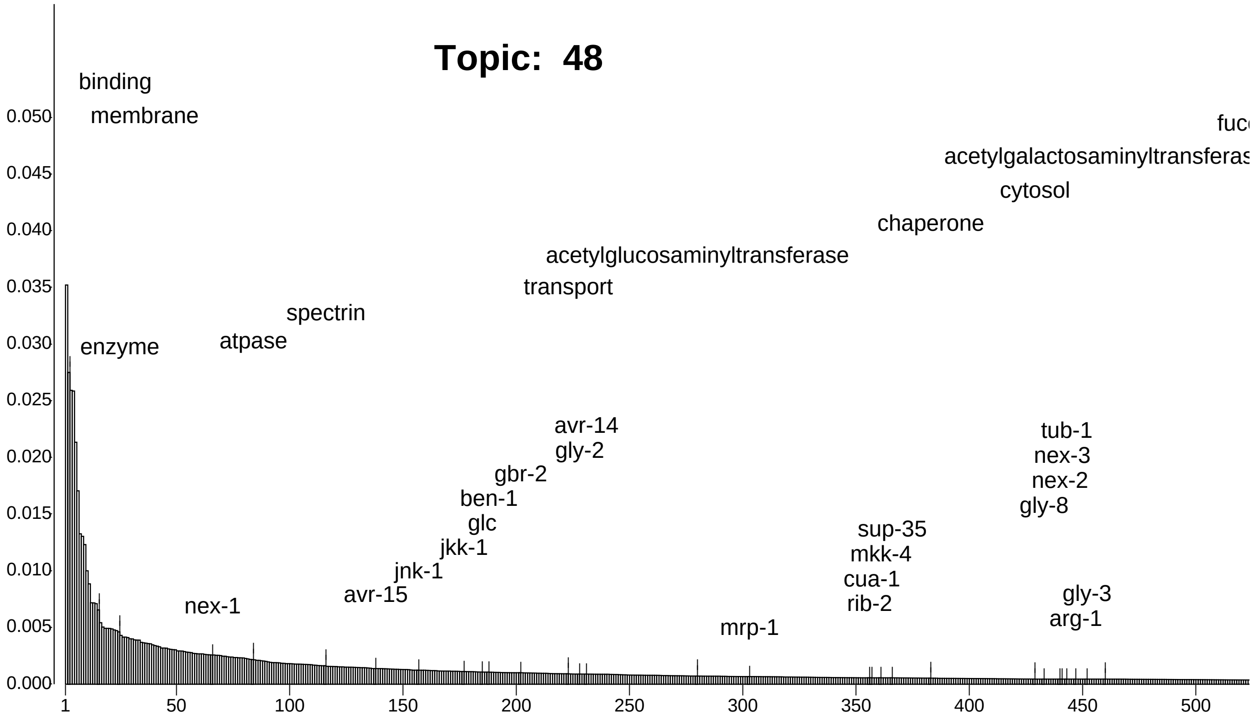

GO-based labels: *MF:* amino acid binding, calcium ion binding; *CC:* mitochondrial outer membrane, mitochondrial membrane, peroxisomal membrane; *BP:* **CGC-based labels:** ivermectin, avermectin, glucl, cys, glutamategated, prolyl, mt, nematocidal, pdi, nex1, annexin, bz, ester, mal, metallothionein, mk, ivm, benzimidazole, glu, dissociation, disulfide, analog, phytochelatin, picrotoxin, nh, galnac, cysteinerich, avr15, avermectinsensitive, jnk1, ckii, xaa, b1a, membranebound, valve, cemt, jkk1, contortus, glc, ben1, gammaaminobutyric, hydrolysi, gbr2, betasubunit, ch, detoxification, ionic, detergent, modified, substituted, paraherquamide, dioxapyrrolomycin, gly2, avr14, heparin, lec, published, immobilized, highaffinity

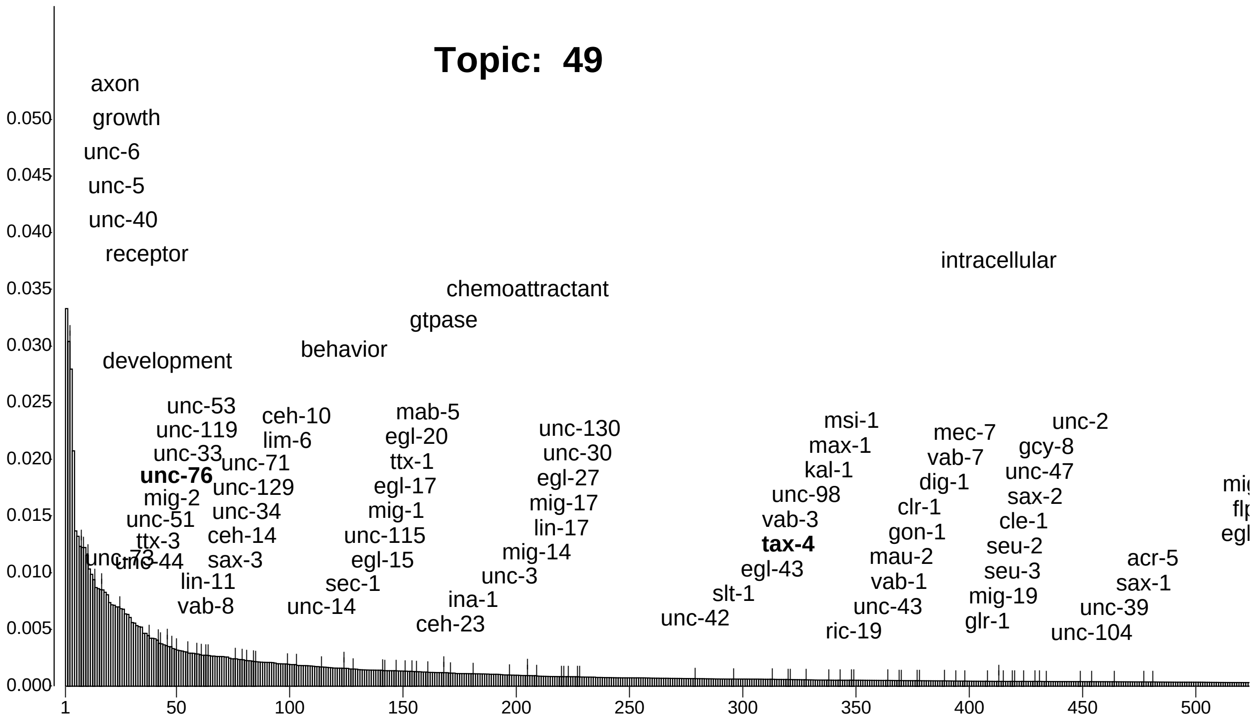

GO-based labels: *MF:* ; *CC:* dynein; *BP:* ethylene mediated signaling pathway, osmosensory signaling pathway via two-component system, abscission, aging, two-component signal transduction system 'phosphorelay', cell wall modification during abscission, determination of adult life span, adipocyte differentiation, endothelial cell differentiation, adult feeding behavior, negative regulation of transcription by pheromones, positive regulation of transcription by pheromones, regulation of transcription from pol i promoter mitotic, regulation of adipocyte differentiation, regulation of endothelial cell differentiation, ethylene mediated signaling pathway 'induced systemic resistance', ethylene mediated signaling pathway 'jasmonic acid/ethylene dependent systemic resistance', mapkkk cascade 'osmolarity sensing', negative regulation of transcription from pol i promoter mitotic, positive regulation of transcription from pol i promoter mitotic, adult behavior, negative regulation of transcription from pol ii promoter by pheromones, positive regulation of transcription from pol ii promoter by pheromones **CGC-based labels:** axon, guidance, unc40, cone, netrin, unc73, outgrowth, migrating, unc44, ttx3, mig2, unc76, longitudinal, unc53, dorsoventral, vab8, dcc, aiy, pioneer, sax3, circumferential, branching, ceh14, unc34, unc129, unc71, midline, migrations, lim6, ceh10, bundle, thermosensory, unc14, pathfinding, repulsion, posteriorly, sdqr, sec1, axons, afd, unc115, fasciculation, sax, mig1, anteriorly, repulsive, ceh23, munc18, ica69, placement, thermotaxi, motoneurone, ncs, pioneering, pitx2, guided, chemoattractant, mig14, db, dorsally, mig17, path, spike, longitudinally, ventrally, fascicle, vps41, robo, aiz
